# Supplementary material for: Fixed vs adjusted-dose benznidazole for adults with chronic Chagas disease without cardiomyopathy: A systematic review and meta-analysis
Source: PLoS Negl Trop Dis. 2020 Aug 17;14(8):e0008529. doi: 10.1371/journal.pntd.0008529 (PMC7451967; doi:10.1371/journal.pntd.0008529)
Supplement: S1 Text — (DOCX) [file pntd.0008529.s001.docx]

**Fixed vs adjusted-dose benznidazole for adults with chronic Chagas disease without cardiomyopathy: A systematic review and meta-analysis**

# S1: Search strategy

**PubMed (18/11/2018)**

| Search | Query |
| --- | --- |
| [#17](https://www.ncbi.nlm.nih.gov/pubmed) | Search **(#15 AND #16)** |
| [#16](https://www.ncbi.nlm.nih.gov/pubmed) | Search **(((Randomized Controlled Trial[pt] OR Controlled Clinical Trial[pt] OR Randomized Controlled Trials[Mesh] OR Random Allocation[Mesh] OR Double-Blind Method[Mesh] OR Single-Blind Method[Mesh] OR Clinical Trial[pt] OR Clinical Trials[Mesh]) OR (Clinical Trial[tw]) OR ((Singl*[tw] OR Doubl*[tw] OR Trebl*[tw] OR Tripl*[tw]) AND (Mask*[tw] OR Blind*[tw])) OR (Placebos[Mesh] OR Placebo*[tw] OR Random*[tw] OR Research Design [mh:noexp]) NOT (Animals [Mesh] NOT Human[Mesh])))** |
| [#15](https://www.ncbi.nlm.nih.gov/pubmed) | Search **(#7 AND #14)** |
| [#14](https://www.ncbi.nlm.nih.gov/pubmed) | Search **(#8 OR #9 OR #10 OR #11 OR #12 OR #13)** |
| [#13](https://www.ncbi.nlm.nih.gov/pubmed) | Search **T.Cruzi[tiab]** |
| [#12](https://www.ncbi.nlm.nih.gov/pubmed) | Search **Cruzi[tiab]** |
| [#11](https://www.ncbi.nlm.nih.gov/pubmed) | Search **Trypanosoma Cruzi[Mesh]** |
| [#10](https://www.ncbi.nlm.nih.gov/pubmed) | Search **Trypanosomiasis[tiab]** |
| [#9](https://www.ncbi.nlm.nih.gov/pubmed) | Search **Chagas[tiab]** |
| [#8](https://www.ncbi.nlm.nih.gov/pubmed) | Search **Chagas Disease[Mesh]** |
| [#7](https://www.ncbi.nlm.nih.gov/pubmed) | Search **(#1 OR #2 OR #3 OR #4 OR #5 OR #6)** |
| [#6](https://www.ncbi.nlm.nih.gov/pubmed) | Search **Ragonil[tiab]** |
| [#5](https://www.ncbi.nlm.nih.gov/pubmed) | Search **Abarax[tiab]** |
| [#4](https://www.ncbi.nlm.nih.gov/pubmed) | Search **Radanil[tiab]** |
| [#3](https://www.ncbi.nlm.nih.gov/pubmed) | Search **Benznidazol*[tiab]** |
| [#2](https://www.ncbi.nlm.nih.gov/pubmed) | Search **Benzonidazol*[tiab]** |
| [#1](https://www.ncbi.nlm.nih.gov/pubmed) | Search **Benzonidazole[Supplementary Concept]** |

**LILACS (BVS-EN) 18/12/2018**

| (Benzonidazol$ OR Benznidazol$ OR Radanil OR Abarax OR Ragonil) AND (MH Chagas Disease OR Chagas OR MH Trypanosomiasis OR Trypanosomiasis OR Tripanosomiasis OR MH Trypanosoma Cruzi OR Cruzi OR T.Cruzi) [Words] and (PT Ensayo Clínico Controlado Aleatorio OR PT Ensayo Clínico Controlado OR MH Ensayo Clínico Controlado Aleatorio OR MH Distribución Aleatoria OR MH Método Doble Ciego OR MH Método Simple-Ciego OR PT Ensayo Clínico OR MH Ensayo Clínico OR (clinical trial OR ensayo clinico OR Ensaio clínico OR ((singl$ OR simpl$ OR doubl$ OR trebl$ OR tripl$)))) [Words] |
| --- |

**Central (Cochrane Library-Wiley) 18/12/2018**

ID Search

#1 Benzonidazol*:ti,ab,kw

#2 Benznidazol*:ti,ab,kw

#3 Radanil:ti,ab,kw

#4 Abarax:ti,ab,kw

#5 Ragonil:ti,ab,kw

#6 #1 OR #2 OR #3 OR #4 OR #5 48

#7 MeSH descriptor: [Chagas Disease] explode all trees

#8 Chagas:ti,ab,kw

#9 Trypanosomiasis:ti,ab,kw

#10 MeSH descriptor: [Trypanosoma cruzi] explode all trees

#11 Cruzi:ti,ab,kw

#12 T.Cruzi:ti,ab,kw 41

#13 #7 OR #8 OR #9 OR #10 OR #11 OR #12

#14 #6 AND #13 in Trials

**Cochrane Reviews (Cochrane Library) 18/12/2018**

ID Search

#1 Benzonidazol*:ti,ab,kw

#2 Benznidazol*:ti,ab,kw

#3 Radanil:ti,ab,kw

#4 Abarax:ti,ab,kw

#5 Ragonil:ti,ab,kw

#6 #1 OR #2 OR #3 OR #4 OR #5

#7 MeSH descriptor: [Chagas Disease] explode all trees

#8 Chagas:ti,ab,kw #9 Trypanosomiasis:ti,ab,kw

#10 MeSH descriptor: [Trypanosoma cruzi] explode all trees

#11 Cruzi:ti,ab,kw

#12 T.Cruzi:ti,ab,kw

#13 #7 OR #8 OR #9 OR #10 OR #11 OR #12

#14 #6 AND #13 in Cochrane Reviews

**EMBase (Elsevier) 18/12/2018**

| **No.** | **Query** |
| --- | --- |
| #17 | #15 AND #16 |
| #16 | ('randomized-controlled-trial'/exp OR 'randomized-controlled-trial' OR 'randomization'/exp OR 'randomization' OR 'controlled-study'/exp OR 'controlled-study' OR 'multicenter study'/exp OR 'multicenter study' OR 'phase-3-clinical-trial'/exp OR 'phase-3-clinical-trial' OR 'phase-4-clinical-trial'/exp OR 'phase-4-clinical-trial' OR 'double-blind-procedure'/exp OR 'double-blind-procedure' OR 'single blind-procedure'/exp OR 'single blind-procedure' OR random*:ti,ab OR crossover*:ti,ab OR 'cross over*':ti,ab OR factorial*:ti,ab OR placebo*:ti,ab OR volunteer*:ti,ab OR ((sing*:ti,ab OR doubl*:ti,ab OR trebl*:ti,ab OR tripl*:ti,ab) AND (blind*:ti,ab OR mask*:ti,ab))) NOT (('animals'/exp OR 'animals') NOT (('humans'/exp OR 'humans') AND ('animals'/exp OR 'animals'))) |
| #15 | #7 AND #14 |
| #14 | #8 OR #9 OR #10 OR #11 OR #12 OR #13 |
| #13 | t.cruzi:ti,ab |
| #12 | cruzi:ti,ab |
| #11 | 'trypanosomiasis'/exp |
| #10 | trypanosomiasis:ti,ab |
| #9 | chagas:ti,ab |
| #8 | 'chagas disease'/exp |
| #7 | #1 OR #2 OR #3 OR #4 OR #5 OR #6 |
| #6 | ragonil:ti,ab |
| #5 | abarax:ti,ab |
| #4 | radanil:ti,ab |
| #3 | benznidazol*:ti,ab |
| #2 | benzonidazol*:ti,ab |
| #1 | 'benznidazole'/exp |

**ClinicalTrials.gov 18/12/2018**

**Benznidazol OR Benzonidazol | Chagas OR Cruzi**

**ICTR**

**Benznidazol* OR Benzonidazol* | Chagas OR Cruzi**
