# Supplementary material for: Fixed vs adjusted-dose benznidazole for adults with chronic Chagas disease without cardiomyopathy: A systematic review and meta-analysis
Source: PLoS Negl Trop Dis. 2020 Aug 17;14(8):e0008529. doi: 10.1371/journal.pntd.0008529 (PMC7451967; doi:10.1371/journal.pntd.0008529)
Supplement: S3 Text — (DOCX) [file pntd.0008529.s003.docx]

**Fixed vs adjusted-dose benznidazole for adults with chronic Chagas disease without cardiomyopathy: A systematic review and meta-analysis**

# S3. Detailed description of included and ongoing studies

| Study ID | Methods / Setting Year | Participants | Interventions | Outcomes |
| --- | --- | --- | --- | --- |
| Rodrigues Coura 1997[[1](#_ENREF_1)] | RCT / Brazil 1997 | Adults. All patients had immunofluorescence and complement fixation reaction positives for *T. cruzi* antibodies and at least two xenodiagnosis positives performed before treatment, and they were submitted to clinical examinations, ECG, and X-ray of the heart and esophagus. | All interventions administered for 30 days:  1) BZN (n = 26) 5 mg/kg/day (4 weeks)  2) Nifurtimox (n = 27) 5 mg/kg/day (4 weeks)  3) Placebo (n = 24) | Serological status recorded  Xenodiagnoses recorded |
| E1224[[2](#_ENREF_2)] | Double-blind, randomized phase 2 RCT Bolivia | Study participants eligible for randomization were aged 18–50 years and weighed at least 40 kg | Patients were enrolled randomly and equally into each of the five oral treatment groups:  1) BZN 5 mg/kg/day (60 days)  2) placebo  3) high-dose E1224 total dose 4000 mg (8 weeks),  4) low-dose E1224 total dose 2000 mg (8 weeks),  5) short-dose E1224 total dose 2400 mg (4 weeks). | Primary efficacy endpoint as parasitological response at the end of treatment, determined by serial negative qualitative standardized PCR: three negative PCR results, from three samples of 10 mL collected over 7 days at the end of treatment.  The secondary efficacy endpoints were sustainability of parasitological clearance (negative qualitative PCR results at the end of treatment, and at 4, 6, and 12 months of follow-up); parasite clearance and changes in parasite load (measured by qualitative PCR and quantitative PCR [qPCR] on days 8, 15, 36, end of treatment, and at 4, 6, and 12 months of follow-up); incidence of conversion to negative response in conventional and non-conventional (lytic anti-α-Gal antibodies measured by AT CL-ELISA) serological response (assessed at end of treatment, and at 4, 6, and between treatment arms and placebo. |
| CHAGASAZOL[[3](#_ENREF_3)] | Open label RCT (CHAGASAZOL trial) / Spain 2010-2011 | Patients were eligible for participation in the study if they met the following inclusion criteria: 18 years or older, detection of *T. cruzi* in two different serologic tests, and a positive result of a Realtime polymerase chain­ reaction assay for *T. cruzi* DNA. Exclusion criteria were pre­vious treatment for Chagas disease, current liver disease, plans for travel during the follow-up pe­riod to a country where the disease is endemic (because the patient could be at risk of reinfec­tion), pregnancy, immunosuppression, prolonged QT interval on electrocardiography, and receipt of drugs that could affect the QT interval or in­terfere with metabolism. | Patients who met the enrollment criteria were randomly assigned, in a 1:1:1 ratio:  1) BZN 150 mg twice daily (60 days)  2) Low-dose posaconazole 100 mg twice daily (60 days)  3) High-dose posaconazole 400 mg twice daily (60 days) | Open label RCT (CHAGASAZOL trial) |
| STOP-CHAGAS[[4](#_ENREF_4)] | Multicenter, international double blind RCT (STOP-CHAGAS Trial) / Latin America and Spain | Adult patients (18 to 50 years of age), weighing >60 kg, with evidence of *T. cruzi* infection given by a positive serology result (2 of 3 conventional tests) and duplicate positive RT-PCR for patients without evidence of cardiopathy.  The majority of the patients (93 [77.5%]) were recruited in Argentina, followed by Chile (11 [9.1%]), and Spain (10 [8.3%]), with Colombia, Guatemala, and Mexico recruiting the remaining 6 patients (5%). | 1) Posaconazole 400 mg (10 mL) oral suspension twice daily (60 days)  2) Posaconazole placebo (10 mL) oral suspension twice daily (60 days)  3) Posaconazole + BZN: Posaconazole 400 mg (10 mL) oral suspension twice daily (60 days) and BZN 100 mg oral tablet twice daily (60 days)  4) Benznidazole + Placebo: Posaconazole placebo (10 mL) oral suspension twice daily (60 days) and BZN 100 mg oral tablet twice daily (60 days) | The primary efficacy outcome is the proportion of subjects with persistent negative RT-PCR by day 180; the secondary outcome was negative RT-PCR at 360 days. |
| BENDITA[[5](#_ENREF_5)] | Phase 2 Multicenter, Safety and Efficacy RCT Bolivia 2015 | Adults (>18 to <50 years), weight >50 kg to <80 kg, diagnosis of *T. cruzi* infection by: Conventional serology (a minimum of two positive tests [Conventional ELISA, Recombinant Elisa and/or Indirect Immunofluorescence (IIF)]) | 1. BZN 300 mg/day (8 weeks)  2. BZN 300 mg/day (4 weeks)  3. BZN 300 mg/day – 2 weeks  4. BZN 150 mg/day (4 weeks)  5. BZN 150 mg/day (4 weeks) + E1224  6. BZN 300 mg/day (8 weeks) + E1224 7. Placebo | Parasitological response as determined by serial negative qualitative PCR results (3 negative PCR results, from 3 samples to be collected in the same day) at EOT and sustained parasitological clearance until 6 months follow-up. [Time Frame: From the end of the treatment period up to 6 months.] |
| TRAENA[[6](#_ENREF_6)] | RCT / Argentina 1999 to 2012. Unpublished data. Personal communication | Adults (20 to 55 years) mostly from Argentina and the rest from bordering countries. | 1) BZN (n = 352) 5mg/kg/day (60 days)  2) Placebo (n = 357, 60 days) | PCR  Serology  Cardiovascular mortality, Progression |
| BETTY[[7](#_ENREF_7)] | Double-blinded, non-inferiority RCT  Argentina 2019-2023. | Females of childbearing age (≥13 years), *T. cruzi* seropositivity confirmed by at least two positive tests, live birth.  Exclusion criteria:  • Women residing outside of the provinces of Chaco, Santiago del Estero, or Tucumán.  • Previous trypanocide treatment (BZN or nifurtimox).  • Female sterilization; no intention to use modern contraception methods during treatment.  • Positive pregnancy test.  • History of severe alcohol abuse within two years; renal insufficiency. | BZN 300mg/day (60 days)  BZN 150mg/day (30 days)  The BZN short course low dose scheme will be 150 mg per day for 30 days. The drug will be administered orally in two doses per day: the short course treatment will start with the active drug and then the oral placebo tablet; one 100 mg tablet and one placebo tablet in the morning and one 50 mg tablet and one placebo tablet in the evening for the first 30 days. The last 30 days will be two placebo tablets in the morning and the evening. | Frequency of positive PCR and the parasitic load measured by qPCR immediately after BZN 30d/150mg [Time Frame: 30 days for the 30d course arm, and 60 days for the 60d course arm]. The frequency of positive PCR and the parasitic load measured by qPCR immediately after BZN 30d/150mg will be non-inferior (Non-Inferiority [NI] margin for PCR: 10% absolute difference) to BZN 60d/300mg.  The frequency of positive PCR and the parasitic load measured by qPCR 10 months after BZN 30d/150mg [Time Frame: 10 months from the end of the 60d treatment]. The frequency of positive PCR and the parasitic load measured by qPCR immediately after BZN 30d/150mg will be non-inferior (Non-Inferiority [NI] margin for PCR: 10% absolute difference) to BZN 60d/300mg. |
| CHICAMOCHA 3[[8-10](#_ENREF_8)] | Blind, parallel-group RCT / Colombia | Adults (20 to 55 years), positive serology status to *T. cruzi*, no clinical signs of dilated cardiomyopathy | -Nifurtimox: 8 mg/kg/day, assuming an average weight of 60 kg: 240 mg B.I.D half-dose: 120 mg B.I.D alternative name: Lampit (Bayer)  -BZN: 5 mg/kg/day, assuming an average weight of 60 kg: 150 mg B.I.D half dose: 75 mg B.I.D alternative name: Radanil (Roche), Rochagan (Roche), Abarax (ELEA)  -Placebo two capsules of matching placebo (containing magnesium stearate and cellulose) B.I.D alternative name: Control group  Participants allocated to NFX or BZN will receive either a 60-day (full-dose) or a 120-day (half-dose) active treatment, whereas the control group will receive placebo for 120 days. | Primary outcome:  qPCR for *T. cruzi* [time frame: 12 - 18 months after starting therapy]  Proportion of participants with at least one out of three positive tests (performed at least one week apart from each other)  Secondary outcomes:  -*T. cruzi* positive serology status [time frame: 12 months after starting therapy] Proportion of participants with positive *T. cruzi* serology status  - Mean change in *T. cruzi* antibody titers [time frame: 12 months after starting therapy] Mean change (before-after) in antibody readings as measured with ELISA serology  - Reported adverse reactions [time frame: 60 days after starting therapy] Proportion of participants with at least one of the following a) Reporting hospitalization or inability to work b) stopping study treatment because of adverse reactions /intolerance c) having abnormal levels of at least two biochemical or blood markers |
| MULTIBENZ^[^[^11^](#_ENREF_11)^]^ | A phase 2 clinical double-blinded RCT / Spain, Brazil, Argentina and Colombia. | Adults (>18 years), diagnosis of Chagas disease by two different serological tests. Positive *T. cruzi* PCR in peripheral blood.  Exclusion Criteria:   - Previous treatment with benznidazole or nifurtimox. - Alcohol consumption. - Acute or chronic health problems that could interfere in the assessment of the efficacy or safety of the drug (acute infections, HIV infection, liver or renal impairment, etc.). - Nitroimidazole hypersensitivity. - Concomitant or previous treatment with allopurinol or antifungal drugs. - Pregnancy. | B300/60 BZN 300mg/day p.o. divided in two doses (60 days)  B150/60 BZN 150mg/day p.o. divided in two doses (60 days)  B400/15 BZN 400mg/day p.o. divided in two doses (15 days) | Primary Outcome:  Proportion of patients with negative parasitemia measured by PCR in the first 12 months after starting treatment [Time Frame: 12 months]  Treatment efficacy is assessed by the proportion of patients with negative parasitemia measured by PCR in the first 12 months after starting treatment |
| TESEO[[12](#_ENREF_12)] | Parallel RCT / Bolivia | - Adults, 18-50 years, 40-90 kg. diagnosed as being infected with *T. cruzi* by conventional serology (two positive tests with different antigens) with at least one positive qualitative RT-PCR assay out of three during the screening. - Patient classified as being in the indeterminate form (without clinical manifestations) or early cardiac form (Kushnir 1) of chronic Chagas disease. | BZN 150 mg twice a day (60 days); 150 mg once a day (30 days) and 150 mg once a day (90 days).  Nifurtimox 240 mg twice a day (60 days); 240 mg twice a day (30 days) and 240 mg once a day (90 days). | RT-PCR from end-of-treatment until 36 months of follow-up  Changes Over Time in the Parasitemia by RT-PCR, Conventional Serology "CHAGATEK ELISA", "Chagatest ELISA recombinante", and Non-Conventional Serology Biomarker "Lytic Anti-α-Gal Antibodies" "Anti-KMP11 Antibodies", "Anti-PFR2 Antibodies", "Anti-Peptide 3973 Antibodies", "Trypomastigote Excreted/Secreted Antigens (TESA)" |

**S3. Excluded studies and reason for exclusion**

| **Study ID** | **Reason for exclusion** |
| --- | --- |
| Alvarez 2016  [[13](#_ENREF_13)] | Wrong study design |
| \| Andrade 2004 [[14](#_ENREF_14)] \| \| --- \| | Wrong patient population |
| Apt 1986  [[15](#_ENREF_15)] | Wrong patient population |
| Molina 2013 [[16](#_ENREF_16)] | Wrong intervention |
| Müller Kratz 2018 [[17](#_ENREF_17)] | Wrong study design |
| Pérez-Molina 2009 [[18](#_ENREF_18)] | Wrong study design |
| Sguassero 2015 [[19](#_ENREF_19)] | Wrong study design |
| Sosa-Estani 2004 [[20](#_ENREF_20)] | Wrong intervention |
| Villar 2014 [[21](#_ENREF_21)] | Wrong study design |

# References

1. Rodrigues Coura J, de Abreu LL, Willcox HP, Petana W. [Comparative controlled study on the use of benznidazole, nifurtimox and placebo, in the chronic form of Chagas' disease, in a field area with interrupted transmission. I. Preliminary evaluation]. Rev Soc Bras Med Trop. 1997;30(2):139-44. PubMed PMID: 9148337.

2. Torrico F, Gascon J, Ortiz L, Alonso-Vega C, Pinazo MJ, Schijman A, et al. Treatment of adult chronic indeterminate Chagas disease with benznidazole and three E1224 dosing regimens: a proof-of-concept, randomised, placebo-controlled trial. Lancet Infect Dis. 2018;18(4):419-30. doi: 10.1016/S1473-3099(17)30538-8. PubMed PMID: 29352704.

3. Molina I, Gomez i Prat J, Salvador F, Trevino B, Sulleiro E, Serre N, et al. Randomized trial of posaconazole and benznidazole for chronic Chagas' disease. N Engl J Med. 2014;370(20):1899-908. doi: 10.1056/NEJMoa1313122. PubMed PMID: 24827034.

4. Morillo CA, Waskin H, Sosa-Estani S, Del Carmen Bangher M, Cuneo C, Milesi R, et al. Benznidazole and Posaconazole in Eliminating Parasites in Asymptomatic T. Cruzi Carriers: The STOP-CHAGAS Trial. J Am Coll Cardiol. 2017;69(8):939-47. doi: 10.1016/j.jacc.2016.12.023. PubMed PMID: 28231946.

5. (03378661) B. BENDITA BEnznidazole New Doses Improved Treatment and Associations. 2017.

6. (02386358) T. Etiologic Treatment With Benznidazole in Adult Patients With Chronic Chagas Disease. A Randomized Clinical Trial. 2015.

7. (03672487) B. Short-course Benznidazole Treatment to Reduce Trypanosoma Cruzi Parasitic Load in Women of Reproductive Age. <https://clinicaltrialsgov/ct2/show/NCT03672487>. 2018.

8. (02369978) C. CHICAMOCHA 3 - Equivalence of Usual Interventions for Trypanosomiasis (EQUITY). <https://clinicaltrialsgov/ct2/show/NCT02369978>. 2015.

9. Villar JC, Herrera VM, Perez Carreno JG, Vaquiro Herrera E, Castellanos Dominguez YZ, Vasquez SM, et al. Nifurtimox versus benznidazole or placebo for asymptomatic Trypanosoma cruzi infection (Equivalence of Usual Interventions for Trypanosomiasis - EQUITY): study protocol for a randomised controlled trial. Trials. 2019;20(1):431. Epub 2019/07/17. doi: 10.1186/s13063-019-3423-3. PubMed PMID: 31307503; PubMed Central PMCID: PMCPMC6631895.

10. Villar JC, Herrera VM, Carreno JGP, Herrera EV, Dominguez YZC, Vasquez SM, et al. Correction to: Nifurtimox versus benznidazole or placebo for asymptomatic Trypanosoma cruzi infection (Equivalence of Usual Interventions for Trypanosomiasis - EQUITY): study protocol for a randomised controlled trial. Trials. 2019;20(1):516. Epub 2019/08/21. doi: 10.1186/s13063-019-3630-y. PubMed PMID: 31429793; PubMed Central PMCID: PMCPMC6701118.

11. (03191162) M. Evaluation of Different Benznidazole Regimens for the Treatment of Chronic Chagas Disease. <https://clinicaltrialsgov/ct2/show/NCT03191162>. 2018.

12. (03981523) T. New Therapies and Biomarkers for Chagas Infection. <https://clinicaltrialsgov/show/NCT03981523>. 2019. PubMed PMID: CN-01945435.

13. Álvarez MG, Hernández Y, Bertocchi G, Fernández M, Lococo B, Ramírez JC, et al. New scheme of intermittent benznidazole administration in patients chronically infected with Trypanosoma cruzi: A pilot short-term follow-up study with adult patients. Antimicrobial Agents and Chemotherapy. 2016;60(2):833-7. doi: 10.1128/AAC.00745-15.

14. Andrade AL, Martelli CM, Oliveira RM, Silva SA, Aires AI, Soussumi LM, et al. Short report: benznidazole efficacy among Trypanosoma cruzi-infected adolescents after a six-year follow-up. American journal of tropical medicine and hygiene. 2004;71(5):594‐7. PubMed PMID: CN-00560425.

15. Apt W, Arribada A, Arab F, Ugarte JM, Luksic I, Solé C. Clinical trial of benznidazole and an immunopotentiator against Chagas disease in Chile. Transactions of the Royal Society of Tropical Medicine and Hygiene. 1986;80(6):1010.

16. Molina I, Prat JGI, Salvador F, Treviño B, Serre N, Sulleiro E, et al. Phase IIb clinical trial with posaconazol and benznidazol for chronic Chagas disease. Tropical Medicine and International Health. 2013;18:60. doi: 10.1111/tmi.12162.

17. Müller Kratz J, Garcia Bournissen F, Forsyth CJ, Sosa-Estani S. Clinical and pharmacological profile of benznidazole for treatment of Chagas disease. Expert Review of Clinical Pharmacology. 2018;11(10):943-57. doi: 10.1080/17512433.2018.1509704.

18. Pérez-Molina JA, Pérez-Ayala A, Moreno S, Fernández-González MCMC, Zamora J, López-Velez R. Use of benznidazole to treat chronic Chagas' disease: A systematic review with a meta-analysis. Journal of Antimicrobial Chemotherapy. 2009;64(6):1139-47. doi: 10.1093/jac/dkp357.

19. Sguassero Y, Cuesta CB, Roberts KN, Hicks E, Comandé D, Ciapponi A, et al. Course of chronic Trypanosoma cruzi infection after treatment based on parasitological and serological tests: A systematic review of follow-up studies. PLoS ONE. 2015;10(10):e0139363. doi: 10.1371/journal.pone.0139363.

20. Sosa-Estani S, Armenti A, Araujo G, Viotti R, Lococo B, Ruiz Vera B, et al. Treatment of Chagas disease with benznidazole and thioctic acid. Medicina. 2004;64(1):1‐6. PubMed PMID: CN-00480924.

21. Villar JC, Perez JG, Cortes OL, Riarte A, Pepper M, Marin‐Neto JA, et al. Trypanocidal drugs for chronic asymptomatic Trypanosoma cruzi infection. Cochrane Database of Systematic Reviews. 2014;(5). doi: 10.1002/14651858.CD003463.pub2. PubMed PMID: CD003463.
