# Supplementary material for: Fixed vs adjusted-dose benznidazole for adults with chronic Chagas disease without cardiomyopathy: A systematic review and meta-analysis
Source: PLoS Negl Trop Dis. 2020 Aug 17;14(8):e0008529. doi: 10.1371/journal.pntd.0008529 (PMC7451967; doi:10.1371/journal.pntd.0008529)
Supplement: S4 Text — (DOCX) [file pntd.0008529.s004.docx]

**Fixed vs adjusted-dose benznidazole for adults with chronic Chagas disease without cardiomyopathy: A systematic review and meta-analysis**

# S4: Support for judgement of included studies by risk of bias item

| Study ID | Random sequence generation | | Allocation concealment | | Blinding of participants & personnel | | Blinding of objective outcome assessment | | Blinding of subjective outcome assessment | | Incomplete outcome data | | Selective reporting | | Other bias | |  |
| --- | --- | --- | --- | --- | --- | --- | --- | --- | --- | --- | --- | --- | --- | --- | --- | --- | --- |
| Rodrigues Coura 1997[[1](#_ENREF_1)] | | “The drug was administered to each group randomly” Described to Villar 2014 | | No description of allocation concealment in the paper. Described to Villar 2014 | | No description of blinding. | | No description of blinding of outcome assessment, but the outcome measurement is not likely to be influenced by lack of blinding; | | No description of blinding of outcome assessment, hence it is no clear if the outcome measurement may be influenced by lack of blinding; | | No missing | | No protocol | | No industry support | |
| E1224[[2](#_ENREF_2)] | | A computer-generated randomization list was prepared by an external provider. The list was stratified by centre and used a block size of ten. | | After confirmation that the patient met all entry criteria, the next available randomization number in the corresponding centre (in chronological order) was assigned by the study pharmacist at each site, who then delivered the corresponding package for the masked arms of the study (E1224 or placebo) or prepared the package with an adequate number of tablets for the benznidazole arm and identified it with the patient's number. Treatment packages for the three E1224 regimen and placebo groups were prepared and labelled with numbers corresponding to the randomization list. Study participants, investigators, and the medical and nursing team remained masked to treatment allocation. | | Study participants, investigators, and the medical and nursing team remained masked to treatment allocation. | | Double-blinding was limited to the E1224 and placebo arms. | | Assessor-blind | | The intention-to-treat (ITT) population comprised all randomized patients by their assigned treatment arms (primary analysis set) | | NCT01489228 all outcomes planned were reported | | Sponsor Drugs for Neglected Diseases *initiative* | |
| CHAGASAZOL[[3](#_ENREF_3)] | | Protocol "Trial randomization will be computer generated and performed using blocks of variable size" | | Protocol "Trial randomization will be computer generated and performed using blocks of variable size." | | This is a prospective, multicenter, open-label, randomized, “proof-of-concept” study. The investigator will be unaware of the size of the randomization blocks and will not know each patient’s assignment until the time of inclusion. | | This is a prospective, multicenter, open-label, randomized, “proof-of-concept” study.· No blinding of outcome assessment, but the outcome measurement is not likely to be influenced by lack of blinding. | | This is a prospective, multicenter, open-label, randomized, “proof-of-concept” study. | | None missing | | NCT01162967 all outcomes planned were reported | | Funded by the Ministry of Health, Spain | |
| STOP-CHAGAS[[4](#_ENREF_4)] | | Subjects will be centrally randomized at a 1:1:1:1 ratio to receive one of the 4 treatment assignments according to a computer-generated randomization schedule using an interactive voice response system (IVRS). | | Subjects will be centrally randomized at a 1:1:1:1 ratio to receive one of the 4 treatment assignments according to a computer-generated randomization schedule using an interactive voice response system (IVRS). | | Posaconazole was given in a randomized single-blinded fashion, whereas BNZ was given as an open-label treatment | | Blood samples were blindly analyzed by the Translational Medicine Biomarker group at Merck. As the study design was blinded to the assignment of Posaconazole therapy, samples were collected on all subjects and analyzed for Posaconazole plasma concentration. | | Posaconazole was given in a randomized single-blinded fashion, whereas BNZ was given as an open-label treatment | | "all randomized subjects in their assigned treatment arms (intention to-treat)" | | All outcomes reported (ClinicalTrials.gov Identifier: NCT01377480) | | The study appears to be free of other sources of bias. Sponsor: Merck Sharp & Dohme Corp. Data Access MSD will provide researchers with access to anonymized patient-level data needed to address the specific research question consistent with the requirements noted here. If a request for a full CSR is approved, MSD will provide researchers the CSR in a redacted form that is consistent with the need to protect patient privacy and confidential commercial information. | |
| BENDITA[[5](#_ENREF_5)] | | Allocation: Randomized. Parallel Assignment  Intervention | | Masking: Quadruple (Participant, Care Provider, Investigator, Outcomes  Assessor)  Masking Description:  Double Dummy | | Masking: Quadruple (Participant, Care Provider, Investigator, Outcomes  Assessor)  Masking Description:  Double Dummy | | Masking: Quadruple (Participant, Care Provider, Investigator, Outcomes  Assessor)  Masking Description:  Double Dummy | | Masking: Quadruple (Participant, Care Provider, Investigator, Outcomes  Assessor)  Masking Description:  Double Dummy | | No important missing | | Unpublished data. | | The study appears to be free of other sources of bias | |
| TRAENA[[6](#_ENREF_6)] | | Computer generated list of random numbers including size variable blocks stratified for clinical stage of Chagas disease | | Sequentially numbered drug containers of identical appearance; | | Blinding of participants and key study personnel ensured, and unlikely that the blinding could have been broken | | Blinding of participants and key study personnel ensured, and unlikely that the blinding could have been broken | | Blinding of outcome assessment ensured, and unlikely that the blinding could have been broken | | There was a loss of 21% of recruited patients for clinical outcomes and of serology. | | NCT 02386358. We cannot assess until the publication of the study but all outcomes planned were communicated in the prespecified way. | | The study appears to be free of other sources of bias but a definite assessment cannot be made until the publication of the manuscript. | |

**S4. Chart of risks of bias presented as percentages across all included studies**


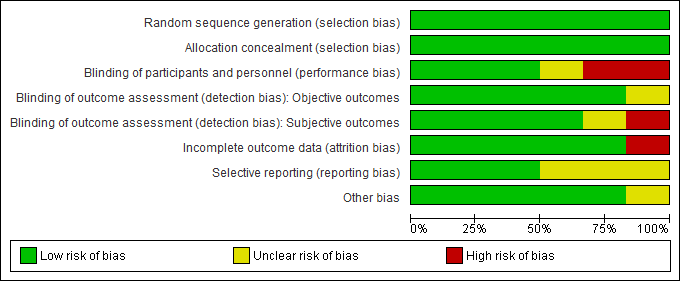


# References

1. Rodrigues Coura J, de Abreu LL, Willcox HP, Petana W. [Comparative controlled study on the use of benznidazole, nifurtimox and placebo, in the chronic form of Chagas' disease, in a field area with interrupted transmission. I. Preliminary evaluation]. Rev Soc Bras Med Trop. 1997;30(2):139-44. PubMed PMID: 9148337.

2. Torrico F, Gascon J, Ortiz L, Alonso-Vega C, Pinazo MJ, Schijman A, et al. Treatment of adult chronic indeterminate Chagas disease with benznidazole and three E1224 dosing regimens: a proof-of-concept, randomised, placebo-controlled trial. Lancet Infect Dis. 2018;18(4):419-30. doi: 10.1016/S1473-3099(17)30538-8. PubMed PMID: 29352704.

3. Molina I, Gomez i Prat J, Salvador F, Trevino B, Sulleiro E, Serre N, et al. Randomized trial of posaconazole and benznidazole for chronic Chagas' disease. N Engl J Med. 2014;370(20):1899-908. doi: 10.1056/NEJMoa1313122. PubMed PMID: 24827034.

4. Morillo CA, Waskin H, Sosa-Estani S, Del Carmen Bangher M, Cuneo C, Milesi R, et al. Benznidazole and Posaconazole in Eliminating Parasites in Asymptomatic T. Cruzi Carriers: The STOP-CHAGAS Trial. J Am Coll Cardiol. 2017;69(8):939-47. doi: 10.1016/j.jacc.2016.12.023. PubMed PMID: 28231946.

5. (03378661) B. BENDITA BEnznidazole New Doses Improved Treatment and Associations. 2017.

6. (02386358) T. Etiologic Treatment With Benznidazole in Adult Patients With Chronic Chagas Disease. A Randomized Clinical Trial. 2015.
