# Supplementary material for: Fixed vs adjusted-dose benznidazole for adults with chronic Chagas disease without cardiomyopathy: A systematic review and meta-analysis
Source: PLoS Negl Trop Dis. 2020 Aug 17;14(8):e0008529. doi: 10.1371/journal.pntd.0008529 (PMC7451967; doi:10.1371/journal.pntd.0008529)
Supplement: S5 Text — (HTM) [file pntd.0008529.s005.htm]

# Benznidazole fixed dose versus ajusted dose for adults with chronic asymptomatic Chagas disease

# Review information

## Authors

Agustín Ciapponi1, Ariel Bardach2, Lucas Perelli3,a, Yanina Sguassero4, Sergio Sosa-Estani3,b

1Argentine Cochrane Centre, Instituto de Efectividad Clínica y Sanitaria (IECS-CONICET), Buenos Aires, Argentina  
2Argentine Cochrane Centre IECS, Institute for Clinical Effectiveness and Health Policy, Southern American Branch of the Iberoamerican Cochrane Centre, Buenos Aires, Argentina  
3[Empty affiliation]  
4Centro Rosarino de Estudios Perinatales, Rosario, Argentina  
aArgentine Cochrane Centre, Instituto de Efectividad Clínica y Sanitaria (IECS-CONICET), Buenos Aires, Argentina  
bHead of Chagas Clinical Programme, DNDi

Citation example: Ciapponi A, Bardach A, Perelli L, Sguassero Y, Sosa-Estani S. Benznidazole fixed dose versus ajusted dose for adults with chronic asymptomatic Chagas disease. Cochrane Database of Systematic Reviews [Year], Issue [Issue].

## Contact person

### Agustín Ciapponi

Argentine Cochrane Centre  
Instituto de Efectividad Clínica y Sanitaria (IECS-CONICET)  
Dr. Emilio Ravignani 2024  
C1414CPV Buenos Aires  
Capital Federal  
Argentina  
  
E-mail: aciapponi@iecs.org.ar  
E-mail 2: aciapponi@gmail.com

## Dates

|  |  |
| --- | --- |
| Assessed as Up-to-date: |  |
| Date of Search: |  |
| Next Stage Expected: |  |
| Protocol First Published: | Not specified |
| Review First Published: | Not specified |
| Last Citation Issue: | Not specified |

## What's new

| Date / Event | Description |
| --- | --- |

## History

| Date / Event | Description |
| --- | --- |

# Abstract

## Background

## Objectives

## Search methods

## Selection criteria

## Data collection and analysis

## Main results

## Authors' conclusions

# Plain language summary

## [Summary title]

[Summary text]

# Background

Chagas disease, is a condition described more than one hundred years ago, that is the result of human infection by Trypanosoma cruzi.

Chagas disease is also known as human American trypanosomiasis, and is endemic in the American continent**1,2**. It is caused by the parasite Trypanosoma cruzi (T. cruzi)

Chagas disease remains a public health threat for 21 Latin American countries, where seven to 12 million people are estimated to be infected and 28 million people remain at high risk. The region carries most of the burden of Chagas disease (99.8%), accounting for at least 662,000 disability‐adjusted life years lost.**3**

The number of infected individuals living in the USA has been estimated as 300,000**4**, with 80,000 additional cases in Europe (about two‐thirds in Spain).**5** This population migratory dynamics, along with the risk of infection to receptors of blood or solid‐organ donations, or from infected pregnant women to their children, is increasingly making Chagas disease a global problem.**6**

## Description of the condition

Chagas disease, is a condition described more than one hundred years ago, that is the result of human infection by Trypanosoma cruzi.

Chagas disease is also known as human American trypanosomiasis, and is endemic in the American continent**1,2**. It is caused by the parasite Trypanosoma cruzi (T. cruzi)

Chagas disease remains a public health threat for 21 Latin American countries, where seven to 12 million people are estimated to be infected and 28 million people remain at high risk. The region carries most of the burden of Chagas disease (99.8%), accounting for at least 662,000 disability‐adjusted life years lost.**3**

The number of infected individuals living in the USA has been estimated as 300,000**4**, with 80,000 additional cases in Europe (about two‐thirds in Spain).**5** This population migratory dynamics, along with the risk of infection to receptors of blood or solid‐organ donations, or from infected pregnant women to their children, is increasingly making Chagas disease a global problem.**6**

Primary acute T. cruzi infection is seldom clinically evident. Serology, and increasingly polymerase chain reactions (PCR), confirm the diagnosis of chronic infection. For the most part, the burden of Chagas disease comes from the 10% to 30% of individuals who develop, after decades of silent infection, chronic chagasic cardiomyopathy (CCC). Once clinically evident, CCC conveys a much worse prognosis than other forms of dilated cardiomyopathy.**7**

Prevention of CCC among infected individuals (i.e. host‐based control) was for many years considered a difficult goal to achieve. Based on that perspective, efforts from public health authorities focused on preventing infection among individuals at risk (i.e. vector‐based control). This policy led to the interruption of the transmission of T. cruzi infection by Triatoma infestans, the most important vector in southern South America in the early 1990s.**8** However, sustaining and extending this achievement to the Andean and Central American countries was limited by logistical, political and biological barriers.**9,10** As a result, it has been recognised that dissemination, emergence and re‐emergence all hinder elimination of T. cruzi infection, now seen as a more elusive goal.

## Description of the intervention

The difficulties found for vector‐based control paralleled a renewed enthusiasm for improving the host‐based control. The first and more logical intervention of this control strategy is treating chronically infected individuals with trypanocidal therapy (TT). Since the 2000s, there has been a growing interest in TT. On the one hand, it was argued that focusing on vector‐based control left the risk of CCC in the population already infected uncovered**.11** On the other hand, scientific findings began to overcome the logistic or methodological barriers regarding the use of TT.

The recommended dose **of** benznidazole (BZN) is usually 5 to 7 mg/kg/day, divided in two or three times daily, for 30 to 90 days. However, no consensus has been achieved regarding TT for chronically infected people. The more frequently reported side effects are skin reactions and neuropathy, which occasionally forces interruption of treatment.

Investigators tested not only the conventional TT nitroderivative drugs available since the 1970s (i.e. nifurtimox (NFTMX) and benznidazole (BZN)) but also clinically approved drugs with possible trypanocidal activity such as allopurinol (ALLOP) or itraconazole (ITRA)

BZN, the most commonly used treatment approved, has a regimen course of 5–7 mg/kg orally, divided into two or three daily doses for 60 days for adults, and 5–10 mg/kg orally, divided into two or three daily doses for 60 days for children up to 12 years old.

## How the intervention might work

## Why it is important to do this review

Recently, some studies published the use of a fixed dose of BZN that should be equivalent to the registered one. A systematic review and meta-analysis it is the best way to determine the efficacy and safety of fixed dose of BZN as treatment for adult

# Objectives

To systematically search, appraise, identify and extract data from eligible studies comparing clinical outcome of adult seropositive individuals to T. cruzi exposed to fixed dose of BZN versus registered dose of BZN.

# Methods

## Criteria for considering studies for this review

### Types of studies

Randomized controlled trials (RCTs) allocating participants to fixed dose of BZN versus registered dose of BZN.

### Types of participants

People with chronic *T. cruzi* infection, as diagnosed with positive serology by at least two of the following techniques: ELISA, indirect haemaglutination (IHA) or indirect immunofluorescence (IIF) without clinically evident (i.e. symptomatic) CCC. For studies without clear distinction of the clinical status of the infected population included, we planned to interpret the information from the description of the population (outpatients not receiving any supportive treatment), or clarify this issue with the authors, aiming at including studies with at least 80% of the population free of symptomatic CCC.

### Types of interventions

Fixed dose of BZN (5-7 mg/k/d) vs registered dose of BZN.

### Types of outcome measures

We included studies when reporting data on outcomes of two types:

- **Parasite‐related outcomes**: they included positive serology (dichotomous); mean reduction of antibodies titres (continuous), positive xenodiagnosis (dichotomous), or positive PCR (dichotomous) after receiving treatment; sustainability of parasitological clearance at different time ponts.
- **Patient‐related outcomes**: (if available):

Eficacy outcomes, such as all‐cause mortality, or significant progression of CCC. We recorded data on sudden death; mortality or hospitalisation due to cardiovascular causes as long as the study source described a treatment‐blinded outcome adjudication process. We defined "significant progression of CCC" was defined as the emergence of substantial changes in the diagnostic tests or clinical status (requiring hospitalisation, implantation of cardiac devices or causing death), or both diagnostic tests and clinical status. For participants with normal electrocardiogram (ECG) at baseline, progression was defined as developing two new ECG abnormalities, or a single ECG abnormality that normally requires medical treatment (e.g. ventricular tachycardia, atrial fibrillation or flutter, complete A‐V block). For studies following Kuschnir's classification criteria16, we will record as progression a change of at least two stages during the follow‐up period. That is, infected individuals with a normal ECG at baseline (stage 0) who developed either cardiomegaly (as documented through any imaging method) or symptoms of heart failure (stage II), and infected asymptomatic individuals with abnormal ECGs at baseline (stage I) who develop symptoms of heart failure (stage III). All deaths during follow‐up will be also recorded as significant progression of CCC.

Safety‐related outcomes, defined as any symptom potentially related with BZN that authors decided to record in treated groups. These outcomes will be categorised as mild‐to‐moderate (e.g. headache, nausea, dyspepsia, pruritus) or severe, including conditions that authors included in this category, described as requiring discontinuation of therapy or hospitalisation (e.g. seizures, fever, Stevens‐Johnson syndrome or severe dermatitis, leukopenia or toxic hepatitis).

We will also assess concentrations of BZN during steady state (days 3–59), at the end of treatment

#### Primary outcomes

1. Any patient‐related outcomes regarding efficacy.
2. Patient‐related outcomes regarding safety.

#### Secondary outcomes

Concentrations of BZN during steady state (days 3–59), at the end of treatment

## Search methods for identification of studies

**Electronic searches**

The following databases were searched: the Cochrane Database of Systematic Reviews (CDSR), Cochrane Central Register of Controlled Trials (CENTRAL), Database of Abstracts of Reviews of Effects (DARE), MEDLINE, EMBASE, LILACS, Clinicaltrials.gov and the WHO International Clinical Trials Registry Platform (ICTRP).

Where necessary, the search terms were modified to suit the requirements of particular databases. No language limitations or publication date restrictions were applied.

For studies with multiple publications, we decided to use the data on a case by case basis through discussion with the principal investigators.

The basic search strategy included the following terms:

(Chagas Disease[Mesh] OR Chagas[tiab] OR Trypanosom\*[tiab] OR Cruzi[tiab] OR T.Cruzi[tiab]) AND (Benzonidazole[Supplementary Concept] OR benznidazol\*[tiab] OR Radanil[tiab] OR Rochagan[tiab] OR N-bencil-2-acetamide[tiab])

The search strategy was adapted to the other databases.

### Electronic searches

### Searching other resources

Additional searches for relevant material were done included a Google search (the first 100 hits, in order of relevance, when typing Chagas benznidazol); handsearching of references lists of systematic reviews and eligible studies retrieved with the electronic search, and questions to experts in the field.

## Data collection and analysis

Selection, data extraction and risk of bias assessment were performed independently by pairs of reviewers of research team. Discrepancies were solved by consensus of the whole team. All phases of the study selection were completed using COVIDENCE®, a web-based platform designed for the process of systematic reviews. Authors of articles were contacted when necessary to obtain missing or supplementary information.

A pre-designed general data extraction form was used after pilot testing. Disagreements were resolved by consensus and, when necessary, a third reviewer was consulted.

We included the following general information:

-Source of study report: publication type, year of publication, journal, authors’ names, and language.

-Study location: geographical region, country, province, city, setting (urban vs. rural).

-Study population: sample size, age at enrollment, living in endemic area under surveillance or not under surveillance, visit to endemic area (even for a brief period), and dates of initiation and ending of data collection.

-Disease: definition of chronic T. cruzi infection, diagnostic tests (number and type of laboratory tests used), quality control measures.

-Benznidazole schedule.

-Follow-up: duration (months), diagnostic tests (number and type of laboratory tests used), quality control, clinical assessment, comparison group.

Collection of individual participant data (IPD)

We contacted primary investigators to invite them to participate in the IPD Meta-analysis by sending an email including:

a) a summary of our systematic review with a link to its protocol in PROSPERO,

b) the study(ies) of interest,

c) a brief online survey to track participation,

e) an agreement of data ownership and co-authorship,

d) a statement about confidentiality of individual participant data,

e) a request for their commitment to help with data management.

If researchers agree to collaborate with our research, then we contacted them to clarify any doubts or queries regarding our project and to provide additional information about how to send their study dataset. We asked the authors of primary studies to supply a unique identification number for each participant. Furthermore, data were sent in electronic format by encrypted e-mail, wherever possible.

### Selection of studies

### Data extraction and management

### Assessment of risk of bias in included studies

### Measures of treatment effect

### Unit of analysis issues

### Dealing with missing data

### Assessment of heterogeneity

### Assessment of reporting biases

In the case that at least 10 studies can be included we will draw funnel-plots. Funnel plot asymmetry could be due to publication bias, but it could also be due to a real relationship between trial size and effect size, such as when larger trials have lower compliance and compliance is positively related to effect size. In general, asymmetry may be due to selection biases (publication bias, delayed publication bias, location biases, selective outcome reporting), poor methodological quality leading to spuriously inflated effects in smaller studies (poor methodological design, inadequate analysis, fraud), true heterogeneity or chance.

### Data synthesis

First, we will conduct a traditional aggregate meta-analysis is sufficient. Second, if we are interested in both the occurrence of an event and the time to event, then the best approach is a meta-analysis of individual participant data (IPD).

1) Meta-analysis using aggregate data

We will use Review Manager 5 software package to generate pooled effect estimates, using a random‐effects model (RevMan 2012). All those estimates will include a general statistics for the main effects as well as for the heterogeneity of the data across studies. Summary measures consisted of generating, when appropriate, pooled effect estimates and their 95% confidence intervals (CI) of the outcomes of interest. For dichotomous outcomes (e.g. positive serology after treatment), we computed Mantel‐Haenszel odds ratios (OR).

We will describe statistical heterogeneity of intervention effects by calculating the I2statistic. Thresholds for the interpretation of I2 can be misleading, since the importance of inconsistency depends on several factors but we will interpret as follows:

• 0% to 30%: might not be important;

• 30% to 60%: may represent moderate heterogeneity;

• more than 60%: may represent substantial or considerable heterogeneity.

We will use both a fixed-effect model and a random-effects model and compare them in order to assess the impact of statistical heterogeneity. Because we assume that clinical heterogeneity is very likely to impact on our review results given the nature of the interventions included, we will primarily report the random-effects model results, regardless of statistical evidence for heterogeneity effect sizes. We will calculate all overall effects using inverse variance methods. We will convert continuous data to mean difference (MD) and, if different scales are used, we will firstly compute an standardised mean difference (SMD), and second, an overall MD and overall SMD. For continuous data reported as change scores in some studies and final values in other studies we will analyse these data separately and also combined using the generic inverse variance method.

Where necessary, we will contact the corresponding authors of included studies up to three times to supply any unreported data.

2) Meta-analysis using IPD obtained from original study data sets

As high heterogeneity among included studies is expected, we will consider two strategies to conduct time to event analysis:

To apply a two-stage approach where we will create summary statistics out of the IPD in each study separately (stage 1) and combine the summary data obtained using a standard meta-analysis method (stage 2). We plan to use a standard Stats Package to obtain estimates of treatment effect and standard error (e.g. SAS or STATA) for stage 1, and then we will input data using Generic Inverse Variance Method in Rev Man for stage 2.

### Subgroup analysis and investigation of heterogeneity

If it is possible to secure the necessary data, we will conduct subgroup analyses classifying the trials as follows.

-Age of participants at time of treatment: young adults vs. older population.

-Type of serological test: conventional serology vs. non-conventional serology.

-Time of treatment and testing: early chronic phase (less than ten years) vs. late chronic phase (equal or more than ten years).

-Region where the patient was infected: Central vs. South America. It is expected to find an earlier and higher rate of seroconversion in Central America due to the presence of different parasite lineages, i.e., T. cruzi type I predominating in Central America and T. cruzi type Non I (II, V and VI) in South America.

We will calculate a pooled effect size for each subgroup.

### Sensitivity analysis

We will use sensitivity analyses to assess the impact of risk of bias on the results of primary analyses. For this review, we will undertake sensitivity analyses that determine the effect of restricting the analysis to: (a) only studies with low risk of selection bias (associated with sequence generation or allocation concealment); (b) only studies with low risk of performance bias (associated with issues of blinding); (c) only studies with low risk of attrition bias (associated with completeness of data). In addition, we will assess the sensitivity of findings to any imputed data.

We will investigate the consistency of results applying both fixed-effect and random-effects models and also using OR and RD.

We will analyse ORs and RDs as sensitivity analysis but we will report RR by default because it is much simpler for most readers.

We will also explore the impact of including studies with high levels of missing data in the overall assessment of treatment effect by using sensitivity analyses.

In case of high levels of heterogeneity, we will perform a meta‐regression analysis to explore the relationship between trypanocidal effect and clinical efficacy (in terms of parasite‐related and patient‐related outcomes, respectively). We computed the slope along with their 95% CI using the Stata software package (version 14.1).

Finally, we will use the GRADE profiler software package17, in order to assign a level of evidence around the data extracted and to generate pooled estimates and Cis.

# Results

## Description of studies

### Results of the search

We identified 709 records trough the databases search and one additional record by contacting authors. After removing duplicates 582 records were screened by title and abstract and 22 by full-text. Finally, 10 studies (five of them ongoing studies) were included (Figure 6).

### Included studies

We included five RCTs (Molina 2014; Morillo 2017; Prado 2008 (TRAENA); Rodriges Coura 1997; Torrico 2018) and five ongoing RCTs (BENDITA 2017; BETTY 2018; CHICAMOCHA-3 2015; MULTIBENZ (Berenice) 2018; TRAENA 2015) that will provide data for the IPD meta-analysis. See Characteristics of included studies

### Excluded studies

We excluded 12 studies, three of them were duplicated records. The reason of exclusion of the other nine (Alvarez 2016; Andrade 2004; Apt 1986; Molina 2013; Müller Kratz 2018; Pérez-Molina 2009; Sguassero 2015; Sosa-Estani 2004; Villar 2014) are described in Characteristics of excluded studies

## Risk of bias in included studies

Only one study was study was considered of unclear risk of bias for the randomization domains (Morillo 2017) and Prado 2008 (Prado 2008 (TRAENA)) was considered as unclear allocation concealment.

Two studies were considered of high risk for blinding assessment (Molina 2014; Morillo 2017) and one study for blinding of participants and personnel (Molina 2014). Two studies presented unclear risk for selective reporting (Prado 2008 (TRAENA); Rodriges Coura 1997). See the risk of bias graphs: **Figure 2** and **Figure 3**.

### Allocation (selection bias)

### Blinding (performance bias and detection bias)

### Incomplete outcome data (attrition bias)

### Selective reporting (reporting bias)

### Other potential sources of bias

## Effects of interventions

### 1 Benznidazole fixed vs adjusted dose

There was no study identified under this comparison.

### 2 Benznidazole at different fixed doses

There was two ongoing studies under this comparison (BENDITA 2017a; MULTIBENZ (Berenice) 2018a).

### 3 Benznidazole versus Placebo: efficacy

Rodriges Coura 1997 (no differences between group)

One ongoing trial TRAENA 2015a

3.2.1 Morillo 2017(RR 0.11, 95% CI 0.04 to 0.33; participants = 60; studies = 1; I2 = 0%)

There was one ongoing study CHICAMOCHA-3 2015a

**Positive xenodiagnoses**

Only one study provided data about adjusted dose19: (RR 0.12, 95% CI 0.04 to 0.36; participants = 60; studies = 1).

**Mean reduction PCR load (GMT at 365days)**

Only one study provided data about adjusted dose21: (MD -0.84, 95% CI -1.24 to -0.44; participants = 92; studies = 1)

3.5(MD 0.01, 95% CI -0.17 to 0.19; participants = 92; studies = 1)

3.6 (MD 0.01, 95% CI -0.07 to 0.09; participants = 92; studies = 1)

3.7 (MD -0.57, 95% CI -1.08 to -0.06; participants = 92; studies = 1)

(RR 0.24, 95% CI 0.06 to 0.93; participants = 112; studies = 3; I2 = 81%)

3.9 (RR 0.89, 95% CI 0.62 to 1.26; participants = 713; studies = 1)

3.10 (RR 1.18, 95% CI 0.40 to 3.49; participants = 713; studies = 1)

3.11 (RR 0.38, 95% CI 0.10 to 1.42; participants = 713; studies = 1)

### 4 Benznidazole versus Placebo: safety

### 5 Benznidazole versus Posaconazole

# Discussion

## Summary of main results

## Overall completeness and applicability of evidence

## Quality of the evidence

## Potential biases in the review process

## Agreements and disagreements with other studies or reviews

# Authors' conclusions

## Implications for practice

## Implications for research

# Acknowledgements

# Contributions of authors

# Declarations of interest

# Differences between protocol and review

# Published notes

# Characteristics of studies

## Characteristics of included studies

### BENDITA 2017a

|  |  |
| --- | --- |
| Methods | Phase 2 Multicenter, Safety and Efficacy RCT |
| Participants | Adults (>18 to <50 years), Weight >50 kg to <80 kg, Diagnosis of T. cruzi infection by: Conventional serology (a minimum of two positive tests [Conventional ELISA, Recombinant Elisa and/or Indirect Immunofluorescence (IIF)]) |
| Interventions | 1. Benznidazole 300 mg / day – 8 weeks  2. Benznidazole 300 mg / day – 4 weeks  3. Benznidazole 300 mg / day – 2 weeks  4. Benznidazole 150 mg / day – 4 weeks  5. Benznidazole 150 mg / day – 4 weeks + E1224  6. Benznidazole 300 mg / day – 8 weeks + E1224 |
| Outcomes | Parasitological response as determined by serial negative qualitative PCR results (3 negative PCR results, from 3 samples to be collected in the same day) at EOT and sustained parasitological clearance until 6 months follow-up. [ Time Frame: From the end of the treatment period up to 6 months.] |
| Notes | Bolivia 2015. PI: Faustino Torrico.  Active, not recruiting Actual Enrollment 210. Estimated Study Completion Date July 27, 2018 |

#### Risk of bias table

| Bias | Authors' judgement | Support for judgement |
| --- | --- | --- |
| Random sequence generation (selection bias) | Low risk |  |
| Allocation concealment (selection bias) | Low risk |  |
| Blinding of participants and personnel (performance bias) | Low risk |  |
| Blinding of outcome assessment (detection bias) Objective outcomes | Low risk |  |
| Blinding of outcome assessment (detection bias) Subjective outcomes | Low risk |  |
| Incomplete outcome data (attrition bias) | Low risk |  |
| Selective reporting (reporting bias) | Unclear risk | Unpublished data. |
| Other bias | Low risk |  |

### Molina 2014

|  |  |
| --- | --- |
| Methods | Open label RCT (CHAGASAZOL trial) |
| Participants | Patients Patients were eligible for participation in the study if they met the following inclusion criteria: an age of 18 years or older, detection of T. cruzi on two different serologic tests, and a positive result of a real­time polymerase­chain­reaction (rt­PCR) assay for T. cruzi DNA. Exclusion criteria were pre­ vious treatment for Chagas' disease, current liver disease, plans for travel during the follow­up pe­ riod to a country where the disease is endemic (because the patient could be at risk for reinfec­ tion), pregnancy, immunosuppression, prolonged QT interval on electrocardiography, and receipt of drugs that could affect the QT interval or in­ terfere with metabolism |
| Interventions | Patients who met the en­ rollment criteria were randomly assigned, in a 1:1:1 ratio:  1) Benznidazole at a dose of 150 mg twice daily for 60 days  2) Posaconazole at a dose of 100 mg twice daily for 60 days (low-dose posaconazole)  3) Posaconazole at a dose of 400 mg twice daily for 60 days (high-dose posaconazole) |
| Outcomes | Authors assessed antiparasitic activity by testing for the presence of T. cruzi DNA, using real­time polymerase­ chain­reaction (rt­PCR) assays, during the treatment period and 10 months after the end of treatment.  T. cruzi serologic testing was performed at baseline, 6 months after the com­ pletion of treatment, and at the end of the fol­ low­up period  Safety, and side­effect profile were also assessed |
| Notes | Bolivia 2010-2011 |

#### Risk of bias table

| Bias | Authors' judgement | Support for judgement |
| --- | --- | --- |
| Random sequence generation (selection bias) | Low risk | Protocol "Trial randomization will be computergenerated and performed using blocks of variable size" |
| Allocation concealment (selection bias) | Low risk | Protocol "Trial randomization will be computergenerated and performed using blocks of variable size." |
| Blinding of participants and personnel (performance bias) | High risk | This is a prospective, multicenter, **open-label**, randomized, “proof-ofconcept” study. The investigator will be unaware of the size of the randomization blocks and will not know each patient’s assignment **until** the time of inclusion. |
| Blinding of outcome assessment (detection bias) Objective outcomes | Low risk | This is a prospective, multicenter, **open-label**, randomized, “proof-ofconcept” study.· No blinding of outcome assessment, but the the outcome measurement is not likely to be influenced by lack of blinding; |
| Blinding of outcome assessment (detection bias) Subjective outcomes | High risk | This is a prospective, multicenter, **open-label**, randomized, “proof-ofconcept” study. |
| Incomplete outcome data (attrition bias) | Low risk | No missing |
| Selective reporting (reporting bias) | Low risk | NCT01162967 all outcomes planned were reported |
| Other bias | Low risk | Funded by the Ministry of Health, Spain |

### Morillo 2017

|  |  |
| --- | --- |
| Methods | Multicenter, international double blind RCT (STOP-CHAGAS Trial) |
| Participants | Adult patients (18 to 50 years of age), weighing >60 kg, with evidence of T. cruzi infection given by a positive serology result (2 of 3 conventional tests) and duplicate positive RT-PCR for T and without evidence of catiphaty.  The majority of the patients (93 [77.5%]) were recruited in Argentina, followed by Chile (11 [9.1%]), and Spain (10 [8.3%]), with Colombia, Guatemala, and Mexico recruiting the remaining 6 patients (5%). |
| Interventions | 1) Posaconazole (POS) 400 mg (10 mL) oral suspension twice daily for 60 days 2) Posaconazole placebo (10 mL) oral suspension twice daily for 60 days 3) Posaconazole + Benznidazole: Posaconazole 400 mg (10 mL) oral suspension twice daily for 60 days and benznidazole (BNZ) 100 mg oral tablet twice daily (200-mg daily dose) for 60 days  4) Benznidazole + Placebo: Posaconazole placebo (10 mL) oral suspension twice daily for 60 days and benznidazole 100 mg oral tablet twice daily (200-mg daily dose) for 60 days |
| Outcomes | The primary efficacy outcome is the proportion of subjects with persistent negative RT-PCR by day 180; the secondary outcome was negative RT-PCR at 360 days. |
| Notes | Latin America and Spain |

#### Risk of bias table

| Bias | Authors' judgement | Support for judgement |
| --- | --- | --- |
| Random sequence generation (selection bias) | Low risk | Subjects will be centrally randomized at a 1:1:1:1 ratio to receive one of the 4 treatment assignments according to a computer-generated randomization schedule using an interactive voice response system (IVRS). |
| Allocation concealment (selection bias) | Low risk | Subjects will be centrally randomized at a 1:1:1:1 ratio to receive one of the 4 treatment assignments according to a computer-generated randomization schedule using an interactive voice response system (IVRS). |
| Blinding of participants and personnel (performance bias) | High risk | We conducted the STOP-CHAGAS trial, in which POS was given in a randomized single-blinded fashion, whereas BNZ was given as an open-label treatment |
| Blinding of outcome assessment (detection bias) Objective outcomes | Low risk | Blood samples were blindly analyzed by the Translational Medicine Biomarker group at Merck. As the study design was blinded to the assignment of POS therapy, samples were collected on all subjects and analyzed for POS plasma concentration. |
| Blinding of outcome assessment (detection bias) Subjective outcomes | Low risk | POS was given in a randomized single-blinded fashion, whereas BNZ was given as an open-label treatment |
| Incomplete outcome data (attrition bias) | Low risk | "all randomized subjects in their assigned treatment arms (intention to-treat)" |
| Selective reporting (reporting bias) | Low risk | All outcomes reported (ClinicalTrials.gov Identifier: NCT01377480) |
| Other bias | Low risk | The study appears to be free of other sources of bias. Sponsor:Merck Sharp & Dohme Corp. Data Access MSD will provide researchers with access to anonymized patient-level data needed to address the specific research question consistent with the requirements noted here. If a request for a full CSR is approved, MSD will provide researchers the CSR in a redacted form that is consistent with the need to protect patient privacy and confidential commercial information. |

### Rodriges Coura 1997

|  |  |
| --- | --- |
| Methods | RCT |
| Participants | Adults. All patients had immunofluorescence and complement fixation reactions positives for T. cruzi antibodies and at least two xenodiagnoses positives in three performed before treatment, and they were submitted to clinical examinations, ECG and X-ray of the heart and esophagus. |
| Interventions | All interventions administered for 30 days:  1) BZD (n = 26) 5 mg/kg/day (4 weeks)  2) NFTMX (n = 27) 5 mg/kg/day (4 weeks)  3) Placebo (n = 24) |
| Outcomes | Serological status recorded  Xenodiagnosis recorded |
| Notes | Brazil 1997 |

#### Risk of bias table

| Bias | Authors' judgement | Support for judgement |
| --- | --- | --- |
| Random sequence generation (selection bias) | Low risk | Described to Villar 2014 |
| Allocation concealment (selection bias) | Low risk | Described to Villar 2014 |
| Blinding of participants and personnel (performance bias) | Unclear risk | No description of blinding. |
| Blinding of outcome assessment (detection bias) Objective outcomes | Low risk | No description of blinding of outcome assessment, but the the outcome measurement is not likely to be influenced by lack of blinding; |
| Blinding of outcome assessment (detection bias) Subjective outcomes | Unclear risk | No description of blinding of outcome assessment, hence it is no clear if the the outcome measurement imay be influenced by lack of blinding; |
| Incomplete outcome data (attrition bias) | Low risk | No missing |
| Selective reporting (reporting bias) | Unclear risk | No protocol |
| Other bias | Low risk | No support of industry |

### Torrico 2018

|  |  |
| --- | --- |
| Methods | Double-blind, randomised phase 2 RCT |
| Participants | Study participants Participants eligible for randomisation were aged 18–50 years and weighed at least 40 kg |
| Interventions | Patients were enrolled randomly and equally into each of the five oral treatment groups:  e1) benznidazole (60 days, 5 mg/kg per day),  2) placebo  3) high-dose E1224 (duration 8 weeks, total dose 4000 mg),  4) low-dose E1224 (8 weeks, 2000 mg),  5) short-dose E1224 (4 weeks, 2400 mg). |
| Outcomes | Primary efficacy endpoint as parasitological response at the end of treatment, determined by serial negative qualitative standardised PCR: three negative PCR results, from three samples of 10 mL collected over 7 days at the end of treatment.  The secondary efficacy endpoints were sustainability of parasitological clearance (negative qualitative PCR results at the end of treatment, and at 4, 6, and 12 months of follow-up); parasite clearance and changes in parasite load (measured by qualitative PCR and quantitative PCR [qPCR] on days 8, 15, 36, end of treatment, and at 4, 6, and 12 months of follow-up); incidence of conversion to negative response in conventional and non-conventional (lytic anti-α-Gal antibodies measured by AT CL-ELISA) serological response (assessed at end of treatment, and at 4, 6, andbetween treatment arms and placebo. |
| Notes | Bolivia |

#### Risk of bias table

| Bias | Authors' judgement | Support for judgement |
| --- | --- | --- |
| Random sequence generation (selection bias) | Low risk | A computer-generated randomisation list was prepared by an external provider. The list was stratified by centre and used a block size of ten. |
| Allocation concealment (selection bias) | Low risk | After confirmation that the patient met all entry criteria, the next available randomisation number in the corresponding centre (in chronological order) was assigned by the study pharmacist at each site, who then delivered the corresponding package for the masked arms of the study (E1224 or placebo) or prepared the package with an adequate number of tablets for the benznidazole arm and identified it with the patient's number. Treatment packages for the three E1224 regimen and placebo groups were prepared and labelled with numbers corresponding to the randomisation list. Study participants, investigators, and the medical and nursing team remained masked to treatment allocation. |
| Blinding of participants and personnel (performance bias) | Low risk | Study participants, investigators, and the medical and nursing team remained masked to treatment allocation. |
| Blinding of outcome assessment (detection bias) Objective outcomes | Unclear risk | Double-blinding was limited to the E1224 and placebo arms. |
| Blinding of outcome assessment (detection bias) Subjective outcomes | Low risk | assessor-blind |
| Incomplete outcome data (attrition bias) | Low risk | The intention-to-treat (ITT) population comprised all randomised patients by their assigned treatment arms (primary analysis set) |
| Selective reporting (reporting bias) | Low risk | NCT01489228 all outcomes planned were reported |
| Other bias | Low risk | Sponsor Drugs for Neglected Diseases |

### TRAENA 2015a

|  |  |
| --- | --- |
| Methods | Double Blind RCT |
| Participants | Adults (20 to 55 years) living in urban areas and reactive to at least 2 for serological test performed in Fatala Chaben Institute (ELISA and IFI) , |
| Interventions | Benznidazole at a dose 5 mg/Kg/day until 60 days have been completed or development of non-acceptable toxicity-Other Name: Radanil (Roche Laboratory)  Placebo at a dose 5 m/Kg/day until 60 daysOther Name: Placebo pills  Exclusion Criteria:  · Patients with chronic Chagas disease who have received prior treatment with benznidazole  · Other cardiomyopathies : idiopathic , alcoholic , peripartum myocarditis, secondary to coronary artery disease, valve disease, hypertension, restrictive, hypertrophic or congenital  · Chronic renal disease  · Bleeding disorders  · History of liver disease or current liver disease ,  · Any other severe clinical disease that decreases their life expectancy  · History of severe allergies  · Pregnant patients  · Patients who have not signed the informed consent. |
| Outcomes | Primary Outcome  · Cardiovascular Mortality [ Time Frame: Time to event: from date of randomization until the date of first documented progression or date of death from any cause up to 10 years of follow-up ]Sudden death, unexpectedly in time and in its presentation,preceded by the abrupt loss of consciousness within a maximum of one hour of the onset of symptoms,when it happened during sleep or unexpectedly in a patient was stable until then. Related Death, when presented in a patient with signs of progressive heart failure.Ischemic or Hemorrhagic Stroke  · Development of heart failure [ Time Frame: Time to event: from date of randomization until the date of first documented progression of heart failure up to 10 years of follow-up ]Dyspnea is evaluated according to the classification of the New York Heart Association (NYHA),gallop rhythm, jugular venous distension, crackles in the lungs, edema or pleural effusion,hepatomegaly  · Severe arrhythmias with hemodynamic compromise or pacemaker implant or Implantable cardiac defibrillator [ Time Frame: Time to event: from date of randomization until the date of first documented progression up to 10 years of follow-up ]Sustained ventricular tachycardia, atrioventricular block, trifascicular block, Atrial fibrillation  Secondary outcome  · Electrocardiographic endpoints. New development of permanent changes in the electrocardiographic [ Time Frame: Time to event: from date of randomization until the date of first documented as defined in the secondary outcome up to 10 years of follow-up ]Changes in clinical stage in chronic Chagas disease [ Time Frame: Time to event: from date of randomization until the date of first documented as defined in the secondary outcome up to 10 years of follow-up ]Clinical progression  · Enlargement of the left ventricle (LV) detected by echocardiography. [ Time Frame: Time to event: from date of randomization until the date of first documented as defined in the secondary outcome up to 10 years of follow-up ]Clinical Progression  · New Heart Failure [ Time Frame: Time to event: from date of randomization until the date of first documented as defined in the secondary outcome up to 10 years of follow-up ]Clinical Progression  · Stroke [ Time Frame: Time to event: from date of randomization until the date of first documented as defined in the secondary outcome up to 10 years of follow-up ]Clinical progression  · Serological negativization [ Time Frame: time to event: from the date of randomization to the date of the first documented serological negativization that persists until 10 years of follow-up ]by ELISA F29 vs. conventional serology as a late indicator of efficacy or therapeutic failure.  · Development and validation of RT-PCR [ Time Frame: time to event: from the date of randomization to the date of the first documented no detectable RT-PCR that persists until 10 years of follow-up ]Demonstration of RT-PCR as an early indicator of efficacy or therapeutic failure.  · Changes of the secondary objectives during RCT development. New single endpoints [ Time Frame: Since October 2011 during 18 months ]  · Combined clinical endpoints: [ Time Frame: Since October 2011 during 18 months ]Right bundle branch block associated with left anterior hemi-block.Parietal motility disorders of left ventricle by echocardiography (akinesia, hypokinesia, and dyskinesia) associated with impaired left ventricular systolic function.Parietal motility disorders of left ventricle by echocardiogram (Akinesia, Hypokinesia, dyskinesia) and /or impaired LV systolic function associated with new electrocardiographic changes (complete left branch block, right bundle branch block, left anterior hemi-block, ventricular extrasystoles.Occurrence of left ventricle aneurysm point by echocardiogram associated to ventricular arrhythmia (section 2.4.1.4.).Occurrence of left ventricle aneurysm point by echocardiogram associated with depression of LV systolic function by echocardiography. |
| Notes | Argentina. pi: Adellina R Riarte. Primary Completion December 2012 |

#### Risk of bias table

| Bias | Authors' judgement | Support for judgement |
| --- | --- | --- |
| Random sequence generation (selection bias) | Low risk | Used a computer random number generator in blocks, stritified by stage. |
| Allocation concealment (selection bias) | Low risk | Sequentially numbered drug containers of identical appearance. |
| Blinding of participants and personnel (performance bias) | Low risk | Blinding of participants and key study personnel ensured, and unlikely that the blinding could have been broken |
| Blinding of outcome assessment (detection bias) Objective outcomes | Low risk | Blinding of participants and key study personnel ensured, and unlikely that the blinding could have been broken |
| Blinding of outcome assessment (detection bias) Subjective outcomes | Low risk | Blinding of outcome assessment ensured, and unlikely that the blinding could have been broken |
| Incomplete outcome data (attrition bias) | High risk | Loss |
| Selective reporting (reporting bias) | Unclear risk | We cannot assess until the publication of the study |
| Other bias | Unclear risk | The study appears to be free of other sources of bias. |

###### Footnotes

## Characteristics of excluded studies

### Alvarez 2016

|  |  |
| --- | --- |
| Reason for exclusion | Wrong study design |

### Andrade 2004

|  |  |
| --- | --- |
| Reason for exclusion | Wrong patient population |

### Apt 1986

|  |  |
| --- | --- |
| Reason for exclusion | Wrong patient population |

### Molina 2013

|  |  |
| --- | --- |
| Reason for exclusion | Wrong intervention |

### Müller Kratz 2018

|  |  |
| --- | --- |
| Reason for exclusion | Wrong study design |

### Pérez-Molina 2009

|  |  |
| --- | --- |
| Reason for exclusion | Wrong study design |

### Sguassero 2015

|  |  |
| --- | --- |
| Reason for exclusion | Wrong study design |

### Sosa-Estani 2004

|  |  |
| --- | --- |
| Reason for exclusion | Wrong intervention |

### Villar 2014

|  |  |
| --- | --- |
| Reason for exclusion | Wrong study design |

###### Footnotes

## Characteristics of studies awaiting classification

###### Footnotes

## Characteristics of ongoing studies

### BENDITA 2017

|  |  |
| --- | --- |
| Study name | BENDITA |
| Methods | Phase 2 Multicenter, Safety and Efficacy RCT |
| Participants | Adults (>18 to <50 years), Weight >50 kg to <80 kg, Diagnosis of T. cruzi infection by: Conventional serology (a minimum of two positive tests [Conventional ELISA, Recombinant Elisa and/or Indirect Immunofluorescence (IIF)]) |
| Interventions | 1. Benznidazole 300 mg / day – 8 weeks  2. Benznidazole 300 mg / day – 4 weeks  3. Benznidazole 300 mg / day – 2 weeks  4. Benznidazole 150 mg / day – 4 weeks  5. Benznidazole 150 mg / day – 4 weeks + E1224  6. Benznidazole 300 mg / day – 8 weeks + E1224 |
| Outcomes | Parasitological response as determined by serial negative qualitative PCR results (3 negative PCR results, from 3 samples to be collected in the same day) at EOT and sustained parasitological clearance until 6 months follow-up. [ Time Frame: From the end of the treatment period up to 6 months.] |
| Starting date | Bolivia 2015.  Active, not recruiting Actual Enrollment 210. Estimated Study Completion Date July 27, 2018 |
| Contact information | PI: Faustino Torrico. |
| Notes |  |

### BETTY 2018

|  |  |
| --- | --- |
| Study name | BETTY |
| Methods | Double-blinded, non-inferiority RCT |
| Participants | Female of childbearing age (≥13 years), T. cruzi seropositivity confirmed by at least two positive tests, Live birth.  Exclusion Criteria:  · Women residing outside of the provinces of Chaco, Santiago del Estero, or Tucumán.  · Previous trypanocide treatment (BZN or nifurtimox).  · Female sterilization; no intention to use modern contraception methods during treatment.  · Positive pregnancy test.  · History of severe alcohol abuse within two years; renal insufficiency. |
| Interventions | Benznidazole 60/300mg benznidazole (BZN) 300 mg per day for 60 days  Benznidazole 30/150mg benznidazole (BZN) 150 mg per day for 30 days  The BZN short course low dose scheme will be 150 mg per day for 30 days. The drug will be administered orally in two doses per day: the short course treatment will start with the active drug and then placebo oral tablet; one 100 mg tablet and one placebo tablet in the morning and one 50 mg tablet and one placebo tablet in the evening for the first 30 days. The last 30 days will be two placebo tablets in the morning and the evening. |
| Outcomes | Frequency of positive PCR and the parasitic load measured by qPCR immediately after BZN 30d/150mg [ Time Frame: 30 days for the 30d course arm, and 60 days for the 60d course arm ]The frequency of positive PCR and the parasitic load measured by qPCR immediately after BZN 30d/150mg will be non-inferior (Non Inferiority [NI] margin for PCR: 10% absolute difference) to BZN 60d/300mg.  The frequency of positive PCR and the parasitic load measured by qPCR 10 months after BZN 30d/150mg [ Time Frame: 10 months from the end of the 60d treatment ]The frequency of positive PCR and the parasitic load measured by qPCR immediately after BZN 30d/150mg will be non-inferior (Non Inferiority [NI] margin for PCR: 10% absolute difference) to BZN 60d/300mg. |
| Starting date | Argentina 2019-2023. Not yet recruiting. Estimated Enrollment 600. stimated Study Completion May 31, 2023 |
| Contact information | PI: Pierre Buekens. |
| Notes |  |

### CHICAMOCHA-3 2015

|  |  |
| --- | --- |
| Study name | CHICAMOCHA-3 |
| Methods | Blind, parallel-group RCT |
| Participants | Adults (20 to 55 years), Positive serology status to Trypanosoma cruzi, No clinical signs of dilated cardiomyopathy |
| Interventions | Nifurtimox: 8 mg/Kg/day, assuming an average weight of 60 Kg: 240 mg B.I.D Half-dose: 120 mg B.I.D Other Name: Lampit (Bayer)  Benznidazole: 5 mg/Kg/day, assuming an average weight of 60 Kg: 150 mg B.I.D Half dose: 75 mg B.I.D Other Name: Radanil (Roche), Rochagan (Roche), Abarax (ELEA)  PlaceboTwo capsules of matching placebo (contaning Magnesium stearate and cellulose) B.I.D Other Name: Control group |
| Outcomes | Primary outcome:  Quantitative Polymerase Chain Reaction (qPCR) for Trypanosoma cruzi [ Time Frame: 12 - 18 months after starting therapy ]  Proportion of participants with at least one out of three positive tests (performed at least one week apart from each other)  Secondary outcomes:  -T. cruzi positive serology status [ Time Frame: 12 months after starting therapy ] Proportion of participants with positive T. cruyzi serology status  - Mean change in T. cruzi antibody titers [ Time Frame: 12 months after starting therapy ] Mean change (before-after) in antibody readings as measured with ELISA serology  - Reported adverse reactions [ Time Frame: 60 days after starting therapy ] Proportion of participants with at least one of the following a) Reporting hospitalization or inability to work b) Stopping study treatment because of adverse reactions /intolerance c) having abnormal levels of at least two biochemical or blood markers |
| Starting date | Colombia and Argentina Recruiting. N= 500. Estimated Study Completion February 2019 |
| Contact information | PI: Juan C Villar. |
| Notes |  |

### MULTIBENZ (Berenice) 2018

|  |  |
| --- | --- |
| Study name | MULTIBENZ (Berenice) |
| Methods | A phase 2 clinical RCT |
| Participants | Adults (>18 years), diagnosis of Chagas disease through two different serological tests. Positive T. cruzi PCR in peripheral blood.  Exclusion Criteria:  · Previous treatment with Benznidazole or Nifurtimox.  · Alcohol consumption.  · Acute or chronic health problems that could interfere in the assessment of the efficacy or safety of the drug (acute infections, HIV infection, liver or renal impairment, etc).  · Nitroimidazole hipersensitivity.  · Concomitant or previous treatment with allopurinol or antifungal drugs.  · Pregnancy. |
| Interventions | B300/60 Benznidazole 300mg/day p.o. divided in two doses for 60 days  B150/60 Benznidazole 150mg/day p.o. divided in two doses for 60 days  B400/15 Benznidazole 400mg/day p.o. divided in two doses for 15 days |
| Outcomes | Primary Outcome:  Proportion of patients with negative parasitaemia measured by PCR during the first 12 months after starting treatment [ Time Frame: 12 months ]  The treatment efficacy is assessed through the proportion of patients with negative parasitaemia measured by PCR during the first 12 months after starting treatment |
| Starting date | Spain, Brazil, Argentina and Colombia. Estimated Primary Completion. April 2018 |
| Contact information | PI: Israel Molina. |
| Notes |  |

### TRAENA 2015

|  |  |
| --- | --- |
| Study name | TRAENA |
| Methods | Double Blind RCT |
| Participants | Adults (20 to 55 years) living in urban areas and reactive to at least 2 for serological test performed in Fatala Chaben Institute (ELISA and IFI) , |
| Interventions | Benznidazole at a dose 5 mg/Kg/day until 60 days have been completed or development of non-acceptable toxicity-Other Name: Radanil (Roche Laboratory)  Placebo at a dose 5 m/Kg/day until 60 daysOther Name: Placebo pills  Exclusion Criteria:  · Patients with chronic Chagas disease who have received prior treatment with benznidazole  · Other cardiomyopathies : idiopathic , alcoholic , peripartum myocarditis, secondary to coronary artery disease, valve disease, hypertension, restrictive, hypertrophic or congenital  · Chronic renal disease  · Bleeding disorders  · History of liver disease or current liver disease ,  · Any other severe clinical disease that decreases their life expectancy  · History of severe allergies  · Pregnant patients  · Patients who have not signed the informed consent. |
| Outcomes | Primary Outcome  · Cardiovascular Mortality [ Time Frame: Time to event: from date of randomization until the date of first documented progression or date of death from any cause up to 10 years of follow-up ]Sudden death, unexpectedly in time and in its presentation,preceded by the abrupt loss of consciousness within a maximum of one hour of the onset of symptoms,when it happened during sleep or unexpectedly in a patient was stable until then. Related Death, when presented in a patient with signs of progressive heart failure.Ischemic or Hemorrhagic Stroke  · Development of heart failure [ Time Frame: Time to event: from date of randomization until the date of first documented progression of heart failure up to 10 years of follow-up ]Dyspnea is evaluated according to the classification of the New York Heart Association (NYHA),gallop rhythm, jugular venous distension, crackles in the lungs, edema or pleural effusion,hepatomegaly  · Severe arrhythmias with hemodynamic compromise or pacemaker implant or Implantable cardiac defibrillator [ Time Frame: Time to event: from date of randomization until the date of first documented progression up to 10 years of follow-up ]Sustained ventricular tachycardia, atrioventricular block, trifascicular block, Atrial fibrillation  Secondary outcome  · Electrocardiographic endpoints. New development of permanent changes in the electrocardiographic [ Time Frame: Time to event: from date of randomization until the date of first documented as defined in the secondary outcome up to 10 years of follow-up ]Changes in clinical stage in chronic Chagas disease [ Time Frame: Time to event: from date of randomization until the date of first documented as defined in the secondary outcome up to 10 years of follow-up ]Clinical progression  · Enlargement of the left ventricle (LV) detected by echocardiography. [ Time Frame: Time to event: from date of randomization until the date of first documented as defined in the secondary outcome up to 10 years of follow-up ]Clinical Progression  · New Heart Failure [ Time Frame: Time to event: from date of randomization until the date of first documented as defined in the secondary outcome up to 10 years of follow-up ]Clinical Progression  · Stroke [ Time Frame: Time to event: from date of randomization until the date of first documented as defined in the secondary outcome up to 10 years of follow-up ]Clinical progression  · Serological negativization [ Time Frame: time to event: from the date of randomization to the date of the first documented serological negativization that persists until 10 years of follow-up ]by ELISA F29 vs. conventional serology as a late indicator of efficacy or therapeutic failure.  · Development and validation of RT-PCR [ Time Frame: time to event: from the date of randomization to the date of the first documented no detectable RT-PCR that persists until 10 years of follow-up ]Demonstration of RT-PCR as an early indicator of efficacy or therapeutic failure.  · Changes of the secondary objectives during RCT development. New single endpoints [ Time Frame: Since October 2011 during 18 months ]  · Combined clinical endpoints: [ Time Frame: Since October 2011 during 18 months ]Right bundle branch block associated with left anterior hemi-block.Parietal motility disorders of left ventricle by echocardiography (akinesia, hypokinesia, and dyskinesia) associated with impaired left ventricular systolic function.Parietal motility disorders of left ventricle by echocardiogram (Akinesia, Hypokinesia, dyskinesia) and /or impaired LV systolic function associated with new electrocardiographic changes (complete left branch block, right bundle branch block, left anterior hemi-block, ventricular extrasystoles.Occurrence of left ventricle aneurysm point by echocardiogram associated to ventricular arrhythmia (section 2.4.1.4.).Occurrence of left ventricle aneurysm point by echocardiogram associated with depression of LV systolic function by echocardiography. |
| Starting date | Argentina. PI: Adellina R Riarte. Primary Completion December 2012 |
| Contact information | PI: Adellina R Riarte. |
| Notes | Alternative contacts: Nilda Prado ngracielap@yahoo.com.ar Andres Ruiz andresmarianoruiz@gmail.com |

###### Footnotes

# Summary of findings tables

# Additional tables

# References to studies

## Included studies

### BENDITA 2017a

BENDITA. BENDITA BEnznidazole New Doses Improved Treatment and Associations. https://ClinicalTrials.gov/show/NCT03378661 2017.

### Molina 2014

Molina I, Gomez i Prat J, Salvador F, Trevino B, Sulleiro E, Serre N, et al. Randomized trial of posaconazole and benznidazole for chronic Chagas' disease. N Engl J Med 2014;370(20):1899-908.

### Morillo 2017

Morillo C A, Waskin H, Sosa-Estani S, Del Carmen Bangher M, Cuneo C, Milesi R, et al. Benznidazole and Posaconazole in Eliminating Parasites in Asymptomatic T. Cruzi Carriers: The STOP-CHAGAS Trial. J Am Coll Cardiol 2017;69(8):939-47.

### Rodriges Coura 1997

\* Rodrigue Coura, J.de Abreu, L. L.Willcox, H. P.Petana, W.. Comparative controlled study on the use of benznidazole, nifurtimox and placebo, in the chronic form of Chagas' disease, in a field area with interrupted transmission. I. Preliminary evaluation [Estudo comparativo controloado com emprego de benznidazole, nifurtimox e placebo, na forma cronica da doenca de Chagas; em uma area de campo com transmissao interrompida. I. Avalicao preliminar]. Rev Soc Bras Med Trop 1997;30(2):139-44.

### Torrico 2018

Torrico F, Gascon J, Ortiz L, Alonso-Vega C, Pinazo M J, Schijman A, et al. Treatment of adult chronic indeterminate Chagas disease with benznidazole and three E1224 dosing regimens: a proof-of-concept, randomised, placebo-controlled trial. Lancet Infect Dis 2018;18(4):419-30.

### TRAENA 2015a

TRAENA. Etiologic Treatment With Benznidazole in Adult Patients With Chronic Chagas Disease. A Randomized Clinical Trial. https://ClinicalTrials.gov/show/NCT02386358 2015.

## Excluded studies

### Alvarez 2016

Alvarez M G, Hernández Y, Bertocchi G, Fernández M, Lococo B, Ramírez J C, et al. New scheme of intermittent benznidazole administration in patients chronically infected with Trypanosoma cruzi: A pilot short-term follow-up study with adult patients. Antimicrobial Agents and Chemotherapy 2016;60(2):833-7.

### Andrade 2004

Andrade A L, Martelli C M, Oliveira R M, Silva S A, Aires A I, Soussumi L M, et al. Short report: benznidazole efficacy among Trypanosoma cruzi-infected adolescents after a six-year follow-up. American journal of tropical medicine and hygiene 2004;71(5):594â€�597-.

### Apt 1986

Apt W, Arribada A, Arab F, Ugarte J M, Luksic I, SolÃ© C. Clinical trial of benznidazole and an immunopotentiator against Chagas disease in Chile. Transactions of the Royal Society of Tropical Medicine and Hygiene 1986;80(6):1010-.

### Molina 2013

Molina I, Prat J G I, Salvador F, TreviÃ±o B, Serre N, Sulleiro E, et al. Phase IIb clinical trial with posaconazol and benznidazol for chronic Chagas disease. Tropical Medicine and International Health 2013;18:60-.

### Müller Kratz 2018

Müller Kratz J, Garcia Bournissen F, Forsyth C J, Sosa-Estani S. Clinical and pharmacological profile of benznidazole for treatment of Chagas disease. Expert Review of Clinical Pharmacology 2018;11(10):943-57.

### Pérez-Molina 2009

Pérez-MolinaJ A, PÃ©rez-Ayala A, Moreno S, Fernández-González M Carmen M C, Zamora J, López-Velez R. Use of benznidazole to treat chronic Chagas' disease: A systematic review with a meta-analysis. Journal of Antimicrobial Chemotherapy 2009;64(6):1139-47.

### Sguassero 2015

Sguassero Y, Cuesta C B, Roberts K N, Hicks E, Comandé D, Ciapponi A, et al. Course of chronic Trypanosoma cruzi infection after treatment based on parasitological and serological tests: A systematic review of follow-up studies. PLoS ONE 2015;10(10):e0139363.

### Sosa-Estani 2004

Sosa-Estani Sergio, Armenti Alejandro, Araujo Graciela, Viotti Rodolfo, Lococo Bruno, Vera Basilio Ruiz, et al. Tratamiento de la enfermedad de Chagas con benznidazol y acido tioctico^ies. Medicina 2004;64(1):1-6.

### Villar 2014

Villar J C, Perez J G, Cortes O L, Riarte A, Pepper M, Marinâ€�Neto J A, et al. Trypanocidal drugs for chronic asymptomatic Trypanosoma cruzi infection. Cochrane Database of Systematic Reviews 2014, Issue 5. Art. No.: CD003463. DOI: 10.1002/14651858.CD003463.pub2.

## Studies awaiting classification

## Ongoing studies

### BENDITA 2017

###### Unpublished data only

BENDITA. BENDITA BEnznidazole New Doses Improved Treatment and Associations. https://ClinicalTrials.gov/show/NCT03378661 2017.

### BETTY 2018

(03672487) BETTY. Short-course Benznidazole Treatment to Reduce Trypanosoma Cruzi Parasitic Load in Women of Reproductive Age. https://ClinicalTrials.gov/show/NCT03672487 2018.

### CHICAMOCHA-3 2015

CHICAMOCHA 3. CHICAMOCHA 3 - Equivalence of Usual Interventions for Trypanosomiasis (EQUITY). https://ClinicalTrials.gov/show/NCT02369978 2015;CHICAMOCHA 3.

### MULTIBENZ (Berenice) 2018

\* MULTIBENZ. Evaluation of Different Benznidazole Regimens for the Treatment of Chronic Chagas Disease. https://ClinicalTrials.gov/show/NCT03191162 2018.

### TRAENA 2015

TRAENA. Etiologic Treatment With Benznidazole in Adult Patients With Chronic Chagas Disease. A Randomized Clinical Trial. https://ClinicalTrials.gov/show/NCT02386358 2015.

# Other references

## Additional references

## Other published versions of this review

## Classification pending references

# Data and analyses

## 1 Benznidazole fixed vs adjusted dose

| Outcome or Subgroup | Studies | Participants | Statistical Method | Effect Estimate |
| --- | --- | --- | --- | --- |

## 2 Benznidazole at different fixed doses

| Outcome or Subgroup | Studies | Participants | Statistical Method | Effect Estimate |
| --- | --- | --- | --- | --- |

|  |  |  |  |  |
| --- | --- | --- | --- | --- |
| 2.1 Positive PCR (monotherapy) | 1 |  | Risk Ratio (M-H, Random, 95% CI) | Subtotals only |
| 2.1.1 Fixed 150 vs 300 8 weeks (follow-up 360 days) | 1 | 40 | Risk Ratio (M-H, Random, 95% CI) | 1.00 [0.24, 4.18] |
| 2.1.2 Fixed 150 vs 300 4 weeks (follow-up 360 days) | 1 | 40 | Risk Ratio (M-H, Random, 95% CI) | 1.20 [0.27, 5.25] |
| 2.1.3 Fixed 150 vs 300 2 weeks (follow-up 360 days) | 1 | 40 | Risk Ratio (M-H, Random, 95% CI) | 0.86 [0.21, 3.47] |

|  |  |  |  |  |
| --- | --- | --- | --- | --- |
| 2.2 Positive PCR (combined with E1224 3000mg total ) | 1 | 60 | Risk Ratio (M-H, Random, 95% CI) | 0.96 [0.73, 1.25] |
| 2.2.2 150 mg/d for 4 weeks vs 300 for 8 weeks (follow-up 360 days) | 1 | 60 | Risk Ratio (M-H, Random, 95% CI) | 0.96 [0.73, 1.25] |

|  |  |  |  |  |
| --- | --- | --- | --- | --- |
| 2.3 Drug discontinuation due to AEs | 1 | 150 | Risk Ratio (M-H, Random, 95% CI) | 4.36 [0.26, 73.42] |

|  |  |  |  |  |
| --- | --- | --- | --- | --- |
| 2.4 Any Adverse events | 1 | 120 | Risk Ratio (M-H, Random, 95% CI) | 0.93 [0.70, 1.23] |
| 2.4.1 Fixed 150 vs 300 8 weeks (follow-up 360 days) | 1 | 40 | Risk Ratio (M-H, Random, 95% CI) | 0.88 [0.56, 1.36] |
| 2.4.2 Fixed 150 vs 300 4 weeks (follow-up 360 days) | 1 | 40 | Risk Ratio (M-H, Random, 95% CI) | 1.05 [0.65, 1.69] |
| 2.4.3 Fixed 150 vs 300 2 weeks (follow-up 360 days) | 1 | 40 | Risk Ratio (M-H, Random, 95% CI) | 0.86 [0.49, 1.50] |

|  |  |  |  |  |
| --- | --- | --- | --- | --- |
| 2.5 Any Serious Adverse events (SAE) | 1 | 120 | Peto Odds Ratio (Peto, Fixed, 95% CI) | 0.25 [0.01, 6.51] |
| 2.5.2 Fixed 150 vs 300 8 weeks (follow-up 360 days) | 1 | 40 | Peto Odds Ratio (Peto, Fixed, 95% CI) | 0.25 [0.01, 6.51] |
| 2.5.3 Fixed 150 vs 300 4 weeks (follow-up 360 days) | 1 | 40 | Peto Odds Ratio (Peto, Fixed, 95% CI) | Not estimable |
| 2.5.4 Fixed 150 vs 300 2 weeks (follow-up 360 days) | 1 | 40 | Peto Odds Ratio (Peto, Fixed, 95% CI) | Not estimable |

## 3 Benznidazole versus Placebo: efficacy

| Outcome or Subgroup | Studies | Participants | Statistical Method | Effect Estimate |
| --- | --- | --- | --- | --- |

|  |  |  |  |  |
| --- | --- | --- | --- | --- |
| 3.1 Positive serology at one year | 2 | 696 | Risk Ratio (M-H, Random, 95% CI) | 0.94 [0.82, 1.08] |
| 3.1.1 Fixed dose | 0 | 0 | Risk Ratio (M-H, Random, 95% CI) | Not estimable |
| 3.1.3 Adjusted dose | 2 | 696 | Risk Ratio (M-H, Random, 95% CI) | 0.94 [0.82, 1.08] |

|  |  |  |  |  |
| --- | --- | --- | --- | --- |
| 3.2 Positive PCR | 4 | 720 | Risk Ratio (M-H, Random, 95% CI) | 0.20 [0.16, 0.25] |
| 3.2.1 Fixed dose 200 mg/d for 60 days (follow-up 180 days) | 0 | 0 | Risk Ratio (M-H, Random, 95% CI) | Not estimable |
| 3.2.2 Fixed dose 200 mg/d for 60 days (follow-up 360 days) | 1 | 60 | Risk Ratio (M-H, Random, 95% CI) | 0.12 [0.04, 0.36] |
| 3.2.3 Fixed dose 150 mg/d for 4 weeks (follow-up 360 days) | 1 | 60 | Risk Ratio (M-H, Random, 95% CI) | 0.21 [0.10, 0.42] |
| 3.2.4 Fixed dose 300 mg/d any duration of treatment (follow-up 360 days) | 1 | 120 | Risk Ratio (M-H, Random, 95% CI) | 0.21 [0.14, 0.31] |
| 3.2.5 Adjusted dose ( 365 days) | 2 | 480 | Risk Ratio (M-H, Random, 95% CI) | 0.20 [0.14, 0.28] |

|  |  |  |  |  |
| --- | --- | --- | --- | --- |
| 3.3 Positive xenodiagnosis | 1 | 177 | Risk Ratio (M-H, Random, 95% CI) | 0.05 [0.01, 0.22] |
| 3.3.1 Fixed dose | 0 | 0 | Risk Ratio (M-H, Random, 95% CI) | Not estimable |
| 3.3.3 Adjusted dose | 1 | 177 | Risk Ratio (M-H, Random, 95% CI) | 0.05 [0.01, 0.22] |

|  |  |  |  |  |
| --- | --- | --- | --- | --- |
| 3.4 Mean reduction PCR load (GMT at 365days) | 2 | 480 | Std. Mean Difference (IV, Random, 95% CI) | -0.48 [-1.19, 0.23] |
| 3.4.1 Fixed Dose | 0 | 0 | Std. Mean Difference (IV, Random, 95% CI) | Not estimable |
| 3.4.2 Adjusted dose | 2 | 480 | Std. Mean Difference (IV, Random, 95% CI) | -0.48 [-1.19, 0.23] |

|  |  |  |  |  |
| --- | --- | --- | --- | --- |
| 3.5 Mean reduction of antibodies titres (conventional ELISA at 365days) | 1 | 92 | Mean Difference (IV, Random, 95% CI) | 0.01 [-0.17, 0.19] |
| 3.5.1 Fixed Dose | 0 | 0 | Mean Difference (IV, Random, 95% CI) | Not estimable |
| 3.5.2 Adjusted dose | 1 | 92 | Mean Difference (IV, Random, 95% CI) | 0.01 [-0.17, 0.19] |

|  |  |  |  |  |
| --- | --- | --- | --- | --- |
| 3.6 Mean reduction of antibodies titres (AT CL−ELISA at 365days) | 1 | 92 | Mean Difference (IV, Random, 95% CI) | 0.01 [-0.07, 0.09] |
| 3.6.1 Fixed Dose | 0 | 0 | Mean Difference (IV, Random, 95% CI) | Not estimable |
| 3.6.2 Adjusted dose | 1 | 92 | Mean Difference (IV, Random, 95% CI) | 0.01 [-0.07, 0.09] |

|  |  |  |  |  |
| --- | --- | --- | --- | --- |
| 3.7 New ECG abnormalities | 1 | 713 | Risk Ratio (M-H, Random, 95% CI) | 0.95 [0.58, 1.55] |
| 3.7.1 Adjusted dose | 1 | 713 | Risk Ratio (M-H, Random, 95% CI) | 0.95 [0.58, 1.55] |

|  |  |  |  |  |
| --- | --- | --- | --- | --- |
| 3.8 geometric mean (AT CL−ELISA at 365 days) | 1 | 92 | Mean Difference (IV, Random, 95% CI) | -0.57 [-1.08, -0.06] |
| 3.8.1 Fixed Dose | 0 | 0 | Mean Difference (IV, Random, 95% CI) | Not estimable |
| 3.8.2 Adjusted dose | 1 | 92 | Mean Difference (IV, Random, 95% CI) | -0.57 [-1.08, -0.06] |

|  |  |  |  |  |
| --- | --- | --- | --- | --- |
| 3.9 Progression of cardiomyopathy | 1 | 713 | Risk Ratio (M-H, Random, 95% CI) | 0.89 [0.62, 1.26] |
| 3.9.1 Adjusted dose | 1 | 713 | Risk Ratio (M-H, Random, 95% CI) | 0.89 [0.62, 1.26] |

|  |  |  |  |  |
| --- | --- | --- | --- | --- |
| 3.10 Cardiovascular mortality | 1 | 713 | Risk Ratio (M-H, Random, 95% CI) | 1.18 [0.40, 3.49] |
| 3.10.1 Adjusted dose | 1 | 713 | Risk Ratio (M-H, Random, 95% CI) | 1.18 [0.40, 3.49] |

|  |  |  |  |  |
| --- | --- | --- | --- | --- |
| 3.11 Severe arrhythmia/Pacemarker/Heart Failure | 1 | 713 | Risk Ratio (M-H, Random, 95% CI) | 0.38 [0.10, 1.42] |
| 3.11.1 Adjusted dose | 1 | 713 | Risk Ratio (M-H, Random, 95% CI) | 0.38 [0.10, 1.42] |

## 4 Benznidazole versus Placebo: safety

| Outcome or Subgroup | Studies | Participants | Statistical Method | Effect Estimate |
| --- | --- | --- | --- | --- |

|  |  |  |  |  |
| --- | --- | --- | --- | --- |
| 4.1 Drug discontinuation | 3 | 846 | Risk Ratio (M-H, Random, 95% CI) | 7.42 [2.51, 21.91] |
| 4.1.1 Fixed dose | 1 | 60 | Risk Ratio (M-H, Random, 95% CI) | 10.00 [1.36, 73.33] |
| 4.1.2 Adjusted dose | 2 | 786 | Risk Ratio (M-H, Random, 95% CI) | 6.35 [1.27, 31.86] |

|  |  |  |  |  |
| --- | --- | --- | --- | --- |
| 4.2 Any Adverse events | 2 | 210 | Risk Ratio (M-H, Random, 95% CI) | 1.37 [0.89, 2.10] |
| 4.2.1 Fixed dose | 2 | 210 | Risk Ratio (M-H, Random, 95% CI) | 1.37 [0.89, 2.10] |
| 4.2.2 Adjusted dose | 0 | 0 | Risk Ratio (M-H, Random, 95% CI) | Not estimable |

|  |  |  |  |  |
| --- | --- | --- | --- | --- |
| 4.3 Any Serious Adverse events (SAE) | 2 | 210 | Risk Ratio (M-H, Random, 95% CI) | 2.23 [0.38, 13.21] |
| 4.3.1 Fixed dose | 2 | 210 | Risk Ratio (M-H, Random, 95% CI) | 2.23 [0.38, 13.21] |
| 4.3.2 Adjusted dose | 0 | 0 | Risk Ratio (M-H, Random, 95% CI) | Not estimable |

|  |  |  |  |  |
| --- | --- | --- | --- | --- |
| 4.4 SAE: toxic hepatitis | 3 | 919 | Risk Ratio (M-H, Random, 95% CI) | 2.45 [0.65, 9.22] |
| 4.4.1 Fixed dose | 2 | 210 | Risk Ratio (M-H, Random, 95% CI) | 1.75 [0.42, 7.29] |
| 4.4.2 Adjusted dose | 1 | 709 | Risk Ratio (M-H, Random, 95% CI) | 9.13 [0.49, 168.90] |

|  |  |  |  |  |
| --- | --- | --- | --- | --- |
| 4.5 SAE: Arthritis | 1 | 709 | Risk Ratio (M-H, Random, 95% CI) | 10.14 [1.31, 78.81] |
| 4.5.1 Fixed dose | 0 | 0 | Risk Ratio (M-H, Random, 95% CI) | Not estimable |
| 4.5.2 Adjusted dose | 1 | 709 | Risk Ratio (M-H, Random, 95% CI) | 10.14 [1.31, 78.81] |

|  |  |  |  |  |
| --- | --- | --- | --- | --- |
| 4.6 SAE: Peripheral neuropathy | 3 | 919 | Risk Ratio (M-H, Random, 95% CI) | 4.27 [0.94, 19.40] |
| 4.6.1 Fixed dose | 2 | 210 | Risk Ratio (M-H, Random, 95% CI) | 1.52 [0.16, 14.32] |
| 4.6.2 Adjusted dose | 1 | 709 | Risk Ratio (M-H, Random, 95% CI) | 10.14 [1.31, 78.81] |

|  |  |  |  |  |
| --- | --- | --- | --- | --- |
| 4.7 SAE: Lymphadenopathy | 2 | 859 | Peto Odds Ratio (Peto, Fixed, 95% CI) | 7.27 [2.04, 25.89] |
| 4.7.1 Fixed dose | 1 | 150 | Peto Odds Ratio (Peto, Fixed, 95% CI) | 3.49 [0.03, 468.68] |
| 4.7.2 Adjusted dose | 1 | 709 | Peto Odds Ratio (Peto, Fixed, 95% CI) | 7.67 [2.06, 28.54] |

|  |  |  |  |  |
| --- | --- | --- | --- | --- |
| 4.8 SAE: Oedema | 1 | 709 | Risk Ratio (M-H, Random, 95% CI) | 3.85 [1.46, 10.21] |
| 4.8.1 Fixed dose | 0 | 0 | Risk Ratio (M-H, Random, 95% CI) | Not estimable |
| 4.8.2 Adjusted dose | 1 | 709 | Risk Ratio (M-H, Random, 95% CI) | 3.85 [1.46, 10.21] |

|  |  |  |  |  |
| --- | --- | --- | --- | --- |
| 4.9 SAE: Fever | 2 | 859 | Risk Ratio (M-H, Random, 95% CI) | 8.09 [1.26, 51.90] |
| 4.9.1 Fixed dose | 1 | 150 | Risk Ratio (M-H, Random, 95% CI) | 2.31 [0.13, 41.70] |
| 4.9.2 Adjusted dose | 1 | 709 | Risk Ratio (M-H, Random, 95% CI) | 16.23 [2.16, 121.71] |

|  |  |  |  |  |
| --- | --- | --- | --- | --- |
| 4.10 SAE: Severe skin reactions | 1 | 60 | Risk Ratio (M-H, Random, 95% CI) | 3.00 [0.13, 70.83] |
| 4.10.1 Fixed dose | 1 | 60 | Risk Ratio (M-H, Random, 95% CI) | 3.00 [0.13, 70.83] |
| 4.10.2 Adjusted dose | 0 | 0 | Risk Ratio (M-H, Random, 95% CI) | Not estimable |

|  |  |  |  |  |
| --- | --- | --- | --- | --- |
| 4.11 SAE: gastrointestinal | 0 | 0 | Risk Ratio (M-H, Random, 95% CI) | Not estimable |
| 4.11.1 Fixed dose | 0 | 0 | Risk Ratio (M-H, Random, 95% CI) | Not estimable |
| 4.11.2 Adjusted dose | 0 | 0 | Risk Ratio (M-H, Random, 95% CI) | Not estimable |

|  |  |  |  |  |
| --- | --- | --- | --- | --- |
| 4.12 SAE: Abortion spontaneous | 1 | 60 | Risk Ratio (M-H, Random, 95% CI) | 3.00 [0.13, 70.83] |
| 4.12.1 Fixed dose | 1 | 60 | Risk Ratio (M-H, Random, 95% CI) | 3.00 [0.13, 70.83] |
| 4.12.2 Adjusted dose | 0 | 0 | Risk Ratio (M-H, Random, 95% CI) | Not estimable |

|  |  |  |  |  |
| --- | --- | --- | --- | --- |
| 4.13 Non-SAE: Pruritus | 2 | 859 | Risk Ratio (M-H, Random, 95% CI) | 3.22 [2.18, 4.76] |
| 4.13.1 Fixed dose | 1 | 150 | Risk Ratio (M-H, Random, 95% CI) | 4.00 [1.02, 15.76] |
| 4.13.2 Adjusted dose | 1 | 709 | Risk Ratio (M-H, Random, 95% CI) | 3.16 [2.10, 4.74] |

|  |  |  |  |  |
| --- | --- | --- | --- | --- |
| 4.14 Non-SAE: Mild rash | 3 | 919 | Risk Ratio (M-H, Random, 95% CI) | 5.32 [3.66, 7.74] |
| 4.14.1 Fixed dose | 2 | 210 | Risk Ratio (M-H, Random, 95% CI) | 6.24 [2.22, 17.52] |
| 4.14.2 Adjusted dose | 1 | 709 | Risk Ratio (M-H, Random, 95% CI) | 5.19 [3.47, 7.77] |

|  |  |  |  |  |
| --- | --- | --- | --- | --- |
| 4.15 Non-SAE: Moderate rash | 2 | 859 | Risk Ratio (M-H, Random, 95% CI) | 6.42 [3.60, 11.44] |
| 4.15.1 Fixed dose | 1 | 150 | Risk Ratio (M-H, Random, 95% CI) | 8.45 [0.52, 137.06] |
| 4.15.2 Adjusted dose | 1 | 709 | Risk Ratio (M-H, Random, 95% CI) | 6.34 [3.51, 11.45] |

|  |  |  |  |  |
| --- | --- | --- | --- | --- |
| 4.16 Non-SAE: Headache | 2 | 859 | Risk Ratio (M-H, Random, 95% CI) | 1.16 [0.90, 1.50] |
| 4.16.1 Fixed dose | 1 | 150 | Risk Ratio (M-H, Random, 95% CI) | 1.09 [0.57, 2.11] |
| 4.16.2 Adjusted dose | 1 | 709 | Risk Ratio (M-H, Random, 95% CI) | 1.17 [0.88, 1.55] |

|  |  |  |  |  |
| --- | --- | --- | --- | --- |
| 4.17 Non-SAE: Insomnia | 2 | 859 | Peto Odds Ratio (Peto, Fixed, 95% CI) | 2.05 [0.92, 4.58] |
| 4.17.1 Fixed dose | 1 | 150 | Peto Odds Ratio (Peto, Fixed, 95% CI) | 3.49 [0.03, 468.68] |
| 4.17.2 Adjusted dose | 1 | 709 | Peto Odds Ratio (Peto, Fixed, 95% CI) | 2.02 [0.90, 4.56] |

|  |  |  |  |  |
| --- | --- | --- | --- | --- |
| 4.18 Non-SAE: Gastrointestinal | 2 | 210 | Risk Ratio (M-H, Random, 95% CI) | 1.32 [0.71, 2.44] |
| 4.18.1 Fixed dose | 2 | 210 | Risk Ratio (M-H, Random, 95% CI) | 1.32 [0.71, 2.44] |
| 4.18.2 Adjusted dose | 0 | 0 | Risk Ratio (M-H, Random, 95% CI) | Not estimable |

|  |  |  |  |  |
| --- | --- | --- | --- | --- |
| 4.19 Non-SAE: Nervous system disorders | 2 | 210 | Risk Ratio (M-H, Random, 95% CI) | 1.71 [0.58, 5.03] |
| 4.19.1 Fixed dose | 2 | 210 | Risk Ratio (M-H, Random, 95% CI) | 1.71 [0.58, 5.03] |
| 4.19.2 Adjusted dose | 0 | 0 | Risk Ratio (M-H, Random, 95% CI) | Not estimable |

|  |  |  |  |  |
| --- | --- | --- | --- | --- |
| 4.20 Non-SAE: Mild hepatitis (ALAT x 3+) | 1 | 60 | Risk Ratio (M-H, Random, 95% CI) | 2.00 [0.19, 20.90] |
| 4.20.1 Fixed dose | 1 | 60 | Risk Ratio (M-H, Random, 95% CI) | 2.00 [0.19, 20.90] |
| 4.20.2 Adjusted dose | 0 | 0 | Risk Ratio (M-H, Random, 95% CI) | Not estimable |

## 5 Benznidazole versus Posaconazole

| Outcome or Subgroup | Studies | Participants | Statistical Method | Effect Estimate |
| --- | --- | --- | --- | --- |

|  |  |  |  |  |
| --- | --- | --- | --- | --- |
| 5.2 Positive PCR | 2 | 112 | Risk Ratio (M-H, Random, 95% CI) | 0.24 [0.06, 0.93] |
| 5.2.1 Fixed low dose of Posaconazol, follow-up 168 days | 0 | 0 | Risk Ratio (M-H, Random, 95% CI) | Not estimable |
| 5.2.2 Fixed low dose of Posaconazol, follow-up 280 days | 1 | 52 | Risk Ratio (M-H, Random, 95% CI) | 0.42 [0.25, 0.69] |
| 5.2.3 Fixed dose (follow-up 180 days) | 0 | 0 | Risk Ratio (M-H, Random, 95% CI) | Not estimable |
| 5.2.4 Fixed dose (follow-up 360 days) | 1 | 60 | Risk Ratio (M-H, Random, 95% CI) | 0.12 [0.04, 0.36] |

# Figures

## Figure 1 (Analysis 4.1)

Forest plot of comparison: 4 Benznidazole versus Placebo: safety, outcome: 4.1 Drug discontinuation.

## Figure 2

Risk of bias summary: review authors' judgements about each risk of bias item for each included study.

## Figure 3

Risk of bias graph: review authors' judgements about each risk of bias item presented as percentages across all included studies.

## Figure 4 (Analysis 3.1)

Forest plot of comparison: 3 Benznidazole versus Placebo: efficacy, outcome: 3.1 Positive serology at one year.

## Figure 5 (Analysis 3.2)

Forest plot of comparison: 3 Benznidazole versus Placebo: efficacy, outcome: 3.2 Positive PCR.

## Figure 6

Study flow diagram

## Figure 7 (Analysis 4.6)

Forest plot of comparison: 4 Benznidazole versus Placebo: safety, outcome: 4.6 SAE: Peripheral neuropathy.

## Figure 8 (Analysis 4.14)

Forest plot of comparison: 4 Benznidazole versus Placebo: safety, outcome: 4.14 Non-SAE: Mild rash.

# Sources of support

## Internal sources

- No sources of support provided

## External sources

- No sources of support provided

# Feedback

# Appendices

## 1 Search strategy

**PubMed (18/11/2018)**

|  |  |  |  |
| --- | --- | --- | --- |
| Search | Add to builder | Query | Items found |
| #17 | Add | Search **(#15 AND #16)** | 91 |
| #16 | Add | Search **(((Randomized Controlled Trial[pt] OR Controlled Clinical Trial[pt] OR Randomized Controlled Trials[Mesh] OR Random Allocation[Mesh] OR Double-Blind Method[Mesh] OR Single-Blind Method[Mesh] OR Clinical Trial[pt] OR Clinical Trials[Mesh]) OR (Clinical Trial[tw]) OR ((Singl\*[tw] OR Doubl\*[tw] OR Trebl\*[tw] OR Tripl\*[tw]) AND (Mask\*[tw] OR Blind\*[tw])) OR (Placebos[Mesh] OR Placebo\*[tw] OR Random\*[tw] OR Research Design [mh:noexp]) NOT (Animals [Mesh] NOT Human[Mesh])))** | 1668118 |
| #15 | Add | Search **(#7 AND #14)** | 1161 |
| #14 | Add | Search **(#8 OR #9 OR #10 OR #11 OR #12 OR #13)** | 26282 |
| #13 | Add | Search **T.Cruzi[tiab]** | 7961 |
| #12 | Add | Search **Cruzi[tiab]** | 14301 |
| #11 | Add | Search **Trypanosoma Cruzi[Mesh]** | 11122 |
| #10 | Add | Search **Trypanosomiasis[tiab]** | 6579 |
| #9 | Add | Search **Chagas[tiab]** | 11849 |
| #8 | Add | Search **Chagas Disease[Mesh]** | 12388 |
| #7 | Add | Search **(#1 OR #2 OR #3 OR #4 OR #5 OR #6)** | 1261 |
| #6 | Add | Search **Ragonil[tiab]** | 0 |
| #5 | Add | Search **Abarax[tiab]** | 5 |
| #4 | Add | Search **Radanil[tiab]** | 4 |
| #3 | Add | Search **Benznidazol\*[tiab]** | 1111 |
| #2 | Add | Search **Benzonidazol\*[tiab]** | 42 |
| #1 | Add | Search **Benzonidazole[Supplementary Concept]** | 749 |

**LILACS (BVS-EN) 18/12/2018**

|  |
| --- |
| **(Benzonidazol$ OR Benznidazol$ OR Radanil OR Abarax OR Ragonil) AND (MH Chagas Disease OR Chagas OR MH Trypanosomiasis OR Trypanosomiasis OR Tripanosomiasis OR MH Trypanosoma Cruzi OR Cruzi OR T.Cruzi) [Words] and (PT Ensayo Clínico Controlado Aleatorio OR PT Ensayo Clínico Controlado OR MH Ensayo Clínico Controlado Aleatorio OR MH Distribución Aleatoria OR MH Método Doble Ciego OR MH Método Simple-Ciego OR PT Ensayo Clínico OR MH Ensayo Clínico OR (clinical trial OR ensayo clinico OR Ensaio clínico OR ((singl$ OR simpl$ OR doubl$ OR trebl$ OR tripl$)))) [Words]** |
| References found \: **30** [refine] |

**Central (Cochrane Library-Wiley) 18/12/2018**

ID Search Hits

#1 Benzonidazol\*:ti,ab,kw 5

#2 Benznidazol\*:ti,ab,kw 47

#3 Radanil:ti,ab,kw 0

#4 Abarax:ti,ab,kw 1

#5 Ragonil:ti,ab,kw 0

#6 #1 OR #2 OR #3 OR #4 OR #5 48

#7 MeSH descriptor: [Chagas Disease] explode all trees 108

#8 Chagas:ti,ab,kw 192

#9 Trypanosomiasis:ti,ab,kw 51

#10 MeSH descriptor: [Trypanosoma cruzi] explode all trees 25

#11 Cruzi:ti,ab,kw 77

#12 T.Cruzi:ti,ab,kw 41

#13 #7 OR #8 OR #9 OR #10 OR #11 OR #12 239

#14 #6 AND #13 in Trials 42

**Cochrane Reviews (Cochrane Library) 18/12/2018**

ID Search Hits

#1 Benzonidazol\*:ti,ab,kw 5

#2 Benznidazol\*:ti,ab,kw 47

#3 Radanil:ti,ab,kw 0

#4 Abarax:ti,ab,kw 1

#5 Ragonil:ti,ab,kw 0

#6 #1 OR #2 OR #3 OR #4 OR #5 48

#7 MeSH descriptor: [Chagas Disease] explode all trees 108

#8 Chagas:ti,ab,kw 192

#9 Trypanosomiasis:ti,ab,kw 51

#10 MeSH descriptor: [Trypanosoma cruzi] explode all trees 25

#11 Cruzi:ti,ab,kw 77

#12 T.Cruzi:ti,ab,kw 41

#13 #7 OR #8 OR #9 OR #10 OR #11 OR #12 239

#14 #6 AND #13 in Cochrane Reviews 1

**EMBase (Elsevier) 18/12/2018**

|  |  |  |
| --- | --- | --- |
| No. | Query | Results |
| #17 | #15 AND #16 | **525** |
| #16 | ('randomized-controlled-trial'/exp OR 'randomized-controlled-trial' OR 'randomization'/exp OR 'randomization' OR 'controlled-study'/exp OR 'controlled-study' OR 'multicenter study'/exp OR 'multicenter study' OR 'phase-3-clinical-trial'/exp OR 'phase-3-clinical-trial' OR 'phase-4-clinical-trial'/exp OR 'phase-4-clinical-trial' OR 'double-blind-procedure'/exp OR 'double-blind-procedure' OR 'single blind-procedure'/exp OR 'single blind-procedure' OR random\*:ti,ab OR crossover\*:ti,ab OR 'cross over\*':ti,ab OR factorial\*:ti,ab OR placebo\*:ti,ab OR volunteer\*:ti,ab OR ((sing\*:ti,ab OR doubl\*:ti,ab OR trebl\*:ti,ab OR tripl\*:ti,ab) AND (blind\*:ti,ab OR mask\*:ti,ab))) NOT (('animals'/exp OR 'animals') NOT (('humans'/exp OR 'humans') AND ('animals'/exp OR 'animals'))) | **5817155** |
| #15 | #7 AND #14 | **2316** |
| #14 | #8 OR #9 OR #10 OR #11 OR #12 OR #13 | **35193** |
| #13 | t.cruzi:ti,ab | **8932** |
| #12 | cruzi:ti,ab | **15907** |
| #11 | 'trypanosomiasis'/exp | **25525** |
| #10 | trypanosomiasis:ti,ab | **5622** |
| #9 | chagas:ti,ab | **13859** |
| #8 | 'chagas disease'/exp | **15451** |
| #7 | #1 OR #2 OR #3 OR #4 OR #5 OR #6 | **2588** |
| #6 | ragonil:ti,ab | **0** |
| #5 | abarax:ti,ab | **6** |
| #4 | radanil:ti,ab | **3** |
| #3 | benznidazol\*:ti,ab | **1358** |
| #2 | benzonidazol\*:ti,ab | **38** |
| #1 | 'benznidazole'/exp | **2387** |

**ClinicalTrials.gov 18/12/2018**

16 Studies found for: **Benznidazol OR Benzonidazol | Chagas OR Cruzi**

**ICTR**

4 Studies found for: **Benznidazol\* OR Benzonidazol\* | Chagas OR Cruzi**

## 2 Risk of bias Assessment

**Criteria for judging risk of bias in the ‘Risk of bias’ assessment tool of RCTs**

**SEQUENCE GENERATION**

**Was the allocation sequence adequately generated? [Short form: *Adequate sequence generation*?]**

|  |  |
| --- | --- |
| Criteria for a judgement of ‘Low ROB’ (i.e. low risk of bias). | The investigators describe a random component in the sequence generation process such as:  · Referring to a random number table;  · Using a computer random number generator;  · Coin tossing;  · Shuffling cards or envelopes;  · Throwing dice;  · Drawing of lots;  · Minimization\*.    \*Minimization may be implemented without a random element, and this is considered to be equivalent to being random. |
| Criteria for the judgement of ‘High ROB’ (i.e. high risk of bias). | The investigators describe a non-random component in the sequence generation process. Usually, the description would involve some systematic, non-random approach, for example:  · Sequence generated by odd or even date of birth;  · Sequence generated by some rule based on date (or day) of admission;  · Sequence generated by some rule based on hospital or clinic record number.    Other non-random approaches happen much less frequently than the systematic approaches mentioned above and tend to be obvious. They usually involve judgement or some method of non-random categorization of participants, for example:  · Allocation by judgement of the clinician;  · Allocation by preference of the participant;  · Allocation based on the results of a laboratory test or a series of tests;  · Allocation by availability of the intervention. |
| Criteria for the judgement of ‘UNCLEAR’ (uncertain risk of bias). | Insufficient information about the sequence generation process to permit judgement of ‘Low ROB’ or ‘High ROB’. |
| **ALLOCATION CONCEALMENT**  **Was allocation adequately concealed? [Short form: *Allocation concealment*?]** | |
| Criteria for a judgement of ‘Low ROB’ (i.e. low risk of bias). | Participants and investigators enrolling participants could not foresee assignment because one of the following, or an equivalent method, was used to conceal allocation:  · Central allocation (including telephone, web-based and pharmacy-controlled randomization);  · Sequentially numbered drug containers of identical appearance;  · Sequentially numbered, opaque, sealed envelopes. |
| Criteria for the judgement of ‘High ROB’ (i.e. high risk of bias). | Participants or investigators enrolling participants could possibly foresee assignments and thus introduce selection bias, such as allocation based on:  · Using an open random allocation schedule (e.g. a list of random numbers);  · Assignment envelopes were used without appropriate safeguards (e.g. if envelopes were unsealed or non­opaque or not sequentially numbered);  · Alternation or rotation;  · Date of birth;  · Case record number;  · Any other explicitly unconcealed procedure. |
| Criteria for the judgement of ‘UNCLEAR’ (uncertain risk of bias). | Insufficient information to permit judgement of ‘Low ROB’ or ‘High ROB’. This is usually the case if the method of concealment is not described or not described in sufficient detail to allow a definite judgement – for example if the use of assignment envelopes is described, but it remains unclear whether envelopes were sequentially numbered, opaque and sealed. |
| **BLINDING OF PARTICIPANTS, PERSONNEL AND OUTCOME ASSESSORS**  **Was knowledge of the allocated interventions adequately prevented during the study? [Short form: *Blinding*?]** | |
| Criteria for a judgement of ‘Low ROB’ (i.e. low risk of bias). | Any one of the following:  · No blinding, but the review authors judge that the outcome and the outcome measurement are not likely to be influenced by lack of blinding;  · Blinding of participants and key study personnel ensured, and unlikely that the blinding could have been broken;  · Either participants or some key study personnel were not blinded, but outcome assessment was blinded and the non-blinding of others unlikely to introduce bias. |
| Criteria for the judgement of ‘High ROB’ (i.e. high risk of bias). | Any one of the following:  · No blinding or incomplete blinding, and the outcome or outcome measurement is likely to be influenced by lack of blinding;  · Blinding of key study participants and personnel attempted, but likely that the blinding could have been broken;  · Either participants or some key study personnel were not blinded, and the non-blinding of others likely to introduce bias. |
| Criteria for the judgement of ‘UNCLEAR’ (uncertain risk of bias). | Any one of the following:  · Insufficient information to permit judgement of ‘Low ROB’ or ‘High ROB’;  · The study did not address this outcome. |
| **INCOMPLETE OUTCOME DATA**  **Were incomplete outcome data adequately addressed? [Short form: *Incomplete outcome data addressed*?]** | |
| Criteria for a judgement of ‘Low ROB’ (i.e. low risk of bias). | Any one of the following:  · No missing outcome data;  · Reasons for missing outcome data unlikely to be related to true outcome (for survival data, censoring unlikely to be introducing bias);  · Missing outcome data balanced in numbers across intervention groups, with similar reasons for missing data across groups;  · For dichotomous outcome data, the proportion of missing outcomes compared with observed event risk not enough to have a clinically relevant impact on the intervention effect estimate;  · For continuous outcome data, plausible effect size (difference in means or standardized difference in means) among missing outcomes not enough to have a clinically relevant impact on observed effect size;  · Missing data have been imputed using appropriate methods. |
| Criteria for the judgement of ‘High ROB’ (i.e. high risk of bias). | Any one of the following:  · Reason for missing outcome data likely to be related to true outcome, with either imbalance in numbers or reasons for missing data across intervention groups;  · For dichotomous outcome data, the proportion of missing outcomes compared with observed event risk enough to induce clinically relevant bias in intervention effect estimate;  · For continuous outcome data, plausible effect size (difference in means or standardized difference in means) among missing outcomes enough to induce clinically relevant bias in observed effect size;  · ‘As-treated’ analysis done with substantial departure of the intervention received from that assigned at randomization;  · Potentially inappropriate application of simple imputation. |
| Criteria for the judgement of ‘UNCLEAR’ (uncertain risk of bias). | Any one of the following:  · Insufficient reporting of attrition/exclusions to permit judgement of ‘Low ROB’ or ‘High ROB’ (e.g. number randomized not stated, no reasons for missing data provided);  · The study did not address this outcome. |
| **SELECTIVE OUTCOME REPORTING**  **Are reports of the study free of suggestion of selective outcome reporting? [Short form: *Free of selective reporting*?]** | |
| Criteria for a judgement of ‘Low ROB’ (i.e. low risk of bias). | Any of the following:  · The study protocol is available and all of the study’s pre-specified (primary and secondary) outcomes that are of interest in the review have been reported in the pre-specified way;  · The study protocol is not available but it is clear that the published reports include all expected outcomes, including those that were pre-specified (convincing text of this nature may be uncommon). |
| Criteria for the judgement of ‘High ROB’ (i.e. high risk of bias). | Any one of the following:  · Not all of the study’s pre-specified primary outcomes have been reported;  · One or more primary outcomes is reported using measurements, analysis methods or subsets of the data (e.g. subscales) that were not pre-specified;  · One or more reported primary outcomes were not pre-specified (unless clear justification for their reporting is provided, such as an unexpected adverse effect);  · One or more outcomes of interest in the review are reported incompletely so that they cannot be entered in a meta-analysis;  · The study report fails to include results for a key outcome that would be expected to have been reported for such a study. |
| Criteria for the judgement of ‘UNCLEAR’ (uncertain risk of bias). | Insufficient information to permit judgement of ‘Low ROB’ or ‘High ROB’. It is likely that the majority of studies will fall into this category. |
| **OTHER POTENTIAL THREATS TO VALIDITY**  **Was the study apparently free of other problems that could put it at a risk of bias? [Short form: *Free of other bias*?]** | |
| Criteria for a judgement of ‘Low ROB’ (i.e. low risk of bias). | The study appears to be free of other sources of bias. |
| Criteria for the judgement of ‘High ROB’ (i.e. high risk of bias). | There is at least one important risk of bias. For example, the study:  · Had a potential source of bias related to the specific study design used; or  · Stopped early due to some data-dependent process (including a formal-stopping rule); or  · Had extreme baseline imbalance; or  · Has been claimed to have been fraudulent; or  · Had some other problem. |
| Criteria for the judgement of ‘UNCLEAR’ (uncertain risk of bias). | There may be a risk of bias, but there is either:  · Insufficient information to assess whether an important risk of bias exists; or  · Insufficient rationale or evidence that an identified problem will introduce bias. |
