# Supplementary material for: Fixed vs adjusted-dose benznidazole for adults with chronic Chagas disease without cardiomyopathy: A systematic review and meta-analysis
Source: PLoS Negl Trop Dis. 2020 Aug 17;14(8):e0008529. doi: 10.1371/journal.pntd.0008529 (PMC7451967; doi:10.1371/journal.pntd.0008529)
Supplement: Attachment — Submitted filename: PNTD-D-20-00450_R1_reviewer.pdf [file pntd.0008529.s009.pdf]

PLOS Neglected Tropical Diseases

Fixed vs adjusted-dose benznidazole for adults with chronic Chagas disease without cardiomyopathy: A systematic review and meta-analysis

--Manuscript Draft--

**Manuscript Number:**	PNTD-D-20-00450R1
**Full Title:**	Fixed vs adjusted-dose benznidazole for adults with chronic Chagas disease without cardiomyopathy: A systematic review and meta-analysis
**Short Title:**	Fixed vs adjusted-dose benznidazole for adults with Chagas disease
**Article Type:**	Research Article
**Keywords:**	Chagas disease; Trypanosoma cruzi; benznidazole; adjusted-dose; fixed-dose; systematic review; meta-analysis
**Abstract:**	Background<br>Chagas disease is a neglected disease that remains a public health threat, particularly in Latin America. The most important treatment options are nitroimidazole derivatives, such as nifurtimox and benznidazole (BZN). Some studies suggest that for adults seropositive to T. cruzi but without clinically evident chronic Chagas cardiomyopathy (CCC), a simple fixed-dose scheme of BZN could be equivalent to a weight-adjusted dose.<br>Objectives<br>To determine the comparative efficacy and safety of a fixed dose of BZN with an adjusted dose for T. cruzi seropositive adults without CCC.<br>Methods<br>We performed a systematic review and meta-analysis following Cochrane methods, and reported according to the PRISMA statement. We included randomized controlled trials (RCTs) allocating participants to fixed and/or adjusted doses of BZN for T. cruzi seropositive adults without CCC. We searched (December 2019) Cochrane, MEDLINE, EMBASE, LILACS, Clinicaltrials.gov, and International Clinical Trials Registry Platform (ICTRP), and contacted Chagas experts. Selection, data extraction, and risk of bias assessment, using the Cochrane tool, were performed independently by pairs of reviewers. Discrepancies were solved by consensus within the team. Primary outcomes were parasite-related outcomes and efficacy or patient-related safety outcomes.<br>We conducted a meta-analysis using RevMan 5.3 software and used GRADE summary of finding tables to present the certainty of evidence by outcome.<br>Main results<br>We identified 655 records through our search strategy and 10 studies (four of them ongoing) met our inclusion criteria. We did not find any study directly comparing fixed vs adjusted doses of BZN, however, some outcomes allowed subgroup comparisons between fixed and adjusted doses of BZN against placebo. Moderate-certainty evidence suggests no important subgroup differences for positive PCR at one year and for three safety outcomes (drug discontinuation, peripheral neuropathy, and mild rash). The same effect was observed for any serious adverse events (low-certainty evidence). All subgroups showed similar effects (I^2 0% for all these subgroup comparisons but 32% for peripheral neuropathy), supporting the equivalence of BZN schemes.<br>Conclusions<br>There is no direct evidence comparing fixed and adjusted doses of BZN. Based on low to very low certainty of evidence for critical clinical outcomes and moderate certainty of evidence for important outcomes, fixed and adjusted doses may be equivalent in terms of safety and efficacy. An individual patient data network meta-analysis could better address this issue.<br>Registration<br>The study protocol was registered in PROSPERO (CRD42019120905).
**Additional Information:**	
**Question**	**Response**
**Financial Disclosure**	This study was funded by an independent grant from Drugs for Neglected Diseases

Powered by Editorial Manager® and ProduXion Manager® from Aries Systems Corporation

Enter a financial disclosure statement that describes the sources of funding for the work included in this submission. Review the submission guidelines for detailed requirements. View published research articles from *PLOS NTDs* for specific examples.<br><br>This statement is required for submission and **will appear in the published article** if the submission is accepted. Please make sure it is accurate.<br><br>**Unfunded studies**<br><br>Enter: *The author(s) received no specific funding for this work.*<br><br>**Funded studies**<br><br>Enter a statement with the following details:<br>• Initials of the authors who received each award<br>• Grant numbers awarded to each author<br>• The full name of each funder<br>• URL of each funder website<br>• Did the sponsors or funders play any role in the study design, data collection and analysis, decision to publish, or preparation of the manuscript?<br>• **NO** - Include this sentence at the end of your statement: *The funders had no role in study design, data collection and analysis, decision to publish, or preparation of the manuscript.*<br>• **YES** - Specify the role(s) played.<br><br>* typeset	initiative (DNDi) that had no role in the data collection and analysis, decision to publish, or preparation of the manuscript. Two out of the 13 authors are from DNDi.
**Competing Interests**<br><br>Use the instructions below to enter a competing interest statement for this	The authors have declared that no competing interests exist.

submission. On behalf of all authors, disclose any competing interests that could be perceived to bias this work—acknowledging all financial support and any other relevant financial or non-financial competing interests.<br><br>This statement **will appear in the published article** if the submission is accepted. Please make sure it is accurate. View published research articles from *PLOS NTDs* for specific examples.<br><br>**NO authors have competing interests**<br><br>Enter: *The authors have declared that no competing interests exist.*<br><br>**Authors with competing interests**<br><br>Enter competing interest details beginning with this statement:<br><br>*I have read the journal's policy and the authors of this manuscript have the following competing interests: [insert competing interests here]*<br><br>* typeset	
**Data Availability**<br><br>Authors are required to make all data underlying the findings described fully available, without restriction, and from the time of publication. PLOS allows rare exceptions to address legal and ethical concerns. See the PLOS Data Policy and	Yes - all data are fully available without restriction

FAQ for detailed information.<br><br>A Data Availability Statement describing where the data can be found is required at submission. Your answers to this question constitute the Data Availability Statement and **will be published in the article**, if accepted.<br><br>**Important:** Stating 'data available on request from the author' is not sufficient. If your data are only available upon request, select 'No' for the first question and explain your exceptional situation in the text box.<br><br>Do the authors confirm that all data underlying the findings described in their manuscript are fully available without restriction?	
**Describe where the data may be found in full sentences. If you are copying our sample text, replace any instances of XXX with the appropriate details.**<br><br>• If the data are **held or will be held in a public repository**, include URLs, accession numbers or DOIs. If this information will only be available after acceptance, indicate this by ticking the box below. For example: *All XXX files are available from the XXX database (accession number(s) XXX, XXX.*).<br>• If the data are all contained **within the manuscript and/or Supporting Information files**, enter the following: *All relevant data are within the manuscript and its Supporting Information files.*<br>• If neither of these applies but you are able to provide **details of access elsewhere**, with or without limitations,	All data is presented in the supporting information of the manuscript

Powered by Editorial Manager® and ProduXion Manager® from Aries Systems Corporation

please do so. For example:<br><br>*Data cannot be shared publicly because of [XXX]. Data are available from the XXX Institutional Data Access / Ethics Committee (contact via XXX) for researchers who meet the criteria for access to confidential data.*<br><br>*The data underlying the results presented in the study are available from (include the name of the third party and contact information or URL).*<br>• This text is appropriate if the data are owned by a third party and authors do not have permission to share the data.<br><br>* typeset	
Additional data availability information:	

Manuscript clean no symbols

Click here to access/download;Manuscript;Fixed vs adjusted BZN. SR MA clean.pdf

Fixed vs adjusted-dose benznidazole for adults with chronic Chagas disease without cardiomyopathy: A systematic review and meta-analysis

Ciapponi Agustín[1*], Barreira Fabiana[2,3], Perelli Lucas[1], Bardach Ariel[1], Gascón Joaquim[4], Molina Israel[5], Morillo Carlos[6], Prado Nilda[7], Riarte Adelina[7], Torrico Faustino[8], Ribeiro Isabela[9], Villar Juan Carlos[10], Sosa-Estani Sergio[2, 11]*.

1 Centro Cochrane Argentino-Instituto de Efectividad Clínica y Sanitaria (IECS-CONICET), Buenos Aires, Argentina.

2 Drugs for Neglected Diseases *initiative* (DND*i*), Río de Janeiro, Brazil.

3 Epidemiology and Public Health Research Center, CONICET, Buenos Aires, Argentina.

4 Hospital Clínic de Barcelona, Barcelona, España.

5 Hospital Universitari Vall d'Hebron Research Institute, Barcelona, España.

6 McMaster University, Population Health Research Institute, Hamilton, Canada

7 Instituto Nacional de Parasitología Dr. M Fatala Chaben, Buenos Aires, Argentina.

8 Universidad Mayor de San Simón, Cochabamba, Bolivia.

9 Drugs for Neglected Diseases *initiative* (DND*i*), Geneva, Switzerland.

10 Grupo de Cardiología Preventiva, Facultad de Ciencias de la Salud, Universidad Autónoma de Bucaramanga, Bucaramanga, Colombia

11 Centro de Investigaciones Epidemiológicas y Salud Pública (CIESP-IECS). CONICET, Buenos Aires, Argentina

***** Contact authors

Abstract

Background

Chagas disease is a neglected disease that remains a public health threat, particularly in Latin America. The most important treatment options are nitroimidazole derivatives, such as nifurtimox and benznidazole (BZN). Some studies suggest that for adults seropositive to *T. cruzi* but without clinically evident chronic Chagas cardiomyopathy (CCC), a simple fixed-dose scheme of BZN could be equivalent to a weight-adjusted dose.

Objectives

To determine the comparative efficacy and safety of a fixed dose of BZN with an adjusted dose for *T. cruzi* seropositive adults without CCC.

Methods

We ~~performed a systematic review and meta-analysis following~~ Cochrane methods, and reported according to the PRISMA statement. We included randomized controlled trials (RCTs) allocating participants to fixed and/or adjusted doses of BZN for *T. cruzi* seropositive adults without CCC. We searched (December 2019) Cochrane, MEDLINE, EMBASE, LILACS, Clinicaltrials.gov, and International Clinical Trials Registry Platform (ICTRP), and contacted Chagas experts. Selection, data extraction, and risk of bias assessment, using the Cochrane tool~~,~~ were performed independently by pairs of reviewers. Discrepancies were solved by consensus within the team. Primary outcomes were parasite-related outcomes and efficacy or patient-related safety outcomes. We conducted a meta-analysis using RevMan 5.3 software and used GRADE summary of finding tables to present the certainty of evidence by outcome.

Main results

We identified 655 records through our search strategy and 10 studies (four of them ongoing) met our inclusion criteria. We did not find any study directly comparing fixed vs adjusted doses of BZN, however, some outcomes allowed subgroup comparisons between fixed and adjusted doses of BZN against placebo. Moderate-certainty evidence suggests no important subgroup differences for positive PCR at one year and for three safety outcomes (drug discontinuation, peripheral neuropathy, and mild rash). The same effect was observed for any serious adverse events (low-certainty evidence). All subgroups showed similar effects (I^2 0% for all these subgroup comparisons but 32% for peripheral neuropathy), supporting the equivalence of BZN schemes.

Conclusions

There is no direct evidence comparing fixed and adjusted doses of BZN. Based on low to very low certainty of evidence for critical clinical outcomes and moderate certainty of evidence for important outcomes, fixed and adjusted doses may be equivalent in terms of safety and efficacy. An individual patient data network meta-analysis could better address this issue.

Registration

The study protocol was registered in PROSPERO (CRD42019120905).

Author Summary

Chagas disease is a major public health problem that requires, among other control interventions, an optimal trypanocidal therapy that achieves the best possible compliance to cure active infection, mainly in children and young populations, women before they become pregnant to prevent congenital transmission, and chronic populations with a risk of progression to cardiomyopathy who are currently not being treated.

Some studies suggest that a simple fixed-dose scheme of benznidazole could be equivalent to the dose adjusted by weight for the treatment of adults seropositive to *T. cruzi* without clinically evident chronic Chagas cardiomyopathy. To confirm or reject this potential equivalence of schemes, we conducted a rigorous systematic review and meta-analysis of randomized controlled trials by reviewing and analyzing the totality of available literature on the subject. Although we did not find direct evidence addressing this question, it appears that an adjusted dose is probably equivalent in terms of important safety and efficacy outcomes, while the effect on critical outcomes is uncertain. Since we did not find any ongoing study comparing fixed versus adjusted doses of benznidazole, we are conducting an individual patient data network meta-analysis to address this question.

Introduction

Chagas disease (CD), also known as human American trypanosomiasis, is a condition resulting from infection by the parasite *Trypanosoma cruzi* ~~(*T. cruzi*)~~. Chagas disease remains a major public health problem; between five and 18 million people are currently infected and the disease is estimated to cause more than 10,000 deaths annually~~.~~[1] Globally, the annual burden is $627.5 million in health-care costs and 232.000~~[2]~~ to 806,170 disability-adjusted life years (DALYs).[3] The Latin American region bears most of the burden of Chagas disease, accounting for at least 206,000[2] to 662,000 DALYs lost.[4] A study investigating the economic value of a therapeutic Chagas vaccine found that when administering standard of care benznidazole (BZN) to 1000 indeterminate patients, 148 discontinued treatment and 219 progressed to chronic disease, resulting in 119 Chagas-related deaths and 2293 DALYs, costing $18.9 million in lifetime societal costs.[5] Population migration dynamics combined with the increased risk of mothers infecting their unborn children and the increased risk of infection from blood or solid-organ donations, means that ~~Chagas disease~~ has become a global problem.[6] The number of infected individuals has been estimated at 300,000 in the USA,[7] and 80,000 in Europe.[8] Primary acute *T. cruzi* infection is seldom clinically evident, given its lack of defining features~~; it~~ is often asymptomatic or resembles a common viral illness, although more serious outcomes such as myocarditis or meningoencephalitis are possible. During this period, *T. cruzi* trypomastigotes are directly observable in the bloodstream. After this comes an indeterminate chronic phase, during which *T. cruzi* lodges in organ tissue in amastigote form, inducing a specific immune response. While most remain asymptomatic, 30-40% of patients progress to an advanced disease stage, usually years to decades after the initial infection. The advanced chronic phase frequently involves damage to the conduction system of the heart and the myocardium, which can result in heart failure and sudden

death. In the Americas, myocarditis secondary to CD is the most common form of nonischemic cardiomyopathy.[9] In other cases, CD produces gastrointestinal disorders (especially megaesophagus and megacolon), or disorders of the central or peripheral nervous system, particularly in immunocompromised patients. Serology is used to confirm a diagnosis of chronic *T. cruzi* infection and polymerase chain reaction (PCR) contributes to that diagnosis. For years, host-based control was considered a difficult goal to achieve and consequently, in the 1990s, public health authority efforts were focused on primary prevention, *Triatoma infestans*-based control, and control of blood donors to prevent infection of individuals at risk.[10] In recent years, significant progress has been made in the fight against triatomines, which, added to the controls implemented by blood banks, has drastically reduced *T. cruzi* infections by vectors and transfusions. Interest in host-based control, that is treating chronically infected individuals with trypanocidal therapy, has increased.[11, 12] Additionally, the focus on vector-based control has left the already infected population without interventions that are potentially preventive of CCC.[13] Two nitroimidazolic derivatives, BZN and nifurtimox, are the only approved trypanocidal options currently used, with no important differences in their relative efficacy, adverse effects (AEs) and cost.[14] The usual recommended dose of BZN is 5 to 7 mg/kg/day orally (5–10 mg/kg for children up to 12 years old) divided into two or three times daily, for 60 days for adults. The most frequently reported side effects are skin reactions and neuropathy, which commonly result in interruption of treatment.[15] Recently, several studies[16-18] suggested that the use of a simpler fixed dose of BZN may be equivalent to an adjusted dose in terms of effectiveness, simplifying its administration and enhancing compliance. In order to compare the efficacy and safety of both schemes for *T. cruzi* seropositive adults without CCC, we have systematically searched and extracted data from eligible

studies comparing relevant clinical, parasitological, and biochemical outcomes for seropositive adults exposed to fixed and/or adjusted doses of BZN.

Methods

We conducted a systematic review and meta-analysis following Cochrane methods,[19] and the PRISMA statement for reporting.[20, 21] The study protocol was registered in PROSPERO (CRD42019120905).

Eligibility criteria

Randomized controlled trials (RCTs) allocating adults with asymptomatic chronic Chagas disease to fixed and/or adjusted doses BZN vs placebo or other trypanocidal treatments were included. RCTs had to include people with chronic *T. cruzi* infection, diagnosed with positive serology by at least two of the following techniques: ELISA, indirect hemagglutination (IHA), or indirect immunofluorescence (IIF), mainly without clinically evident (i.e. symptomatic) CCC. Important safety and efficacy outcomes, such as positive serology or PCR, any adverse events (AEs) and serious adverse events (SAEs) and critical patient (clinical) related outcomes, such as all-cause mortality or significant progression of CCC, were analyzed.

Search strategy

We searched (December 2019) the following databases: Cochrane Database of Systematic Reviews (CDSR), Cochrane Central Register of Controlled Trials (CENTRAL), Database of Abstracts of Reviews of Effects (DARE), MEDLINE, EMBASE, LILACS, Clinicaltrials.gov, and the WHO International Clinical Trials Registry Platform (ICTRP).

The basic search strategy included the following terms: (Chagas Disease [Mesh] OR Chagas[tiab] OR Trypanosom*[tiab] OR Cruzi[tiab] OR T.Cruzi[tiab]) AND (Benznidazole[Supplementary

Concept] OR benznidazol*[tiab] OR Radanil[tiab] OR Rochagan[tiab] OR N-bencil-2-acetamide[tiab]).

The search strategy was adapted to each database (See **Appendix 1 Search strategy**).

No language limitations or publication date restrictions were applied. For studies with multiple publications, we decided how to best use the data on a case-by-case basis through discussion with the principal investigators.

Additional searches included a Google search (the first 100 hits, in order of relevance, when typing Chagas benznidazol), handsearching of reference lists of systematic reviews and eligible studies retrieved with the electronic search, and verbal feedback from experts in the field.

Screening and data extraction

Selection, data extraction, and risk of bias assessment were performed independently by pairs of reviewers from the research team. Discrepancies were solved by consensus within the team. All the study selection phases were completed using COVIDENCE, a web-based platform designed for the systematic review process. Authors of articles were contacted when necessary to obtain missing or supplementary information.

A pre-designed general data extraction form was used after pilot testing.

We extracted the source of study report, study location and setting, population, disease definition of chronic *T. cruzi* infection, diagnostic tests used (number and type of laboratory tests used), quality control measures, BZN and other treatment schedules, and follow-up measures.

Risk of bias (quality) assessment

Pairs of independent reviewers assessed the risk of bias (quality) using the Cochrane Collaboration tool.[22] See details in **Appendix 2 Risk of bias assessment**. Discrepancies were solved by consensus within the team.

Data synthesis

We conducted a traditional aggregate meta-analysis by using the Review Manager 5.3 software package.[23] Pooled effect estimates and their 95% confidence intervals (CI), when appropriate, were generated using a random-effects model. We reported risk ratios (RR) for dichotomous outcomes (e.g. positive serology after treatment), the Peto odds ratio (OR) for very infrequent outcomes, and the mean difference (MD) for continuous data such as antibody titers. For dichotomous data, we used the Mantel-Haenszel method, and for continuous data, we used the inverse variance method.

We described statistical heterogeneity of intervention effects by calculating the I^2 statistic and we interpreted 0-30% as not important, 30-60% as moderate heterogeneity, and more than 60% as substantial heterogeneity. Since we assume that clinical heterogeneity is very likely to impact our review results, given the nature of the interventions included, we primarily reported the random-effects model results, however, we also applied the fixed effect model as a sensitivity analysis. We calculated all overall effects using inverse variance methods.

Where necessary, we contacted the corresponding authors of included studies up to three times to supply any unreported data.

We planned subgroup analyses (see protocol) by age of participants at time of treatment (young adults vs. older population), type of serological test (conventional serology vs. non-conventional serology), time of treatment and testing (less than ten years vs. equal or more than ten years), and region where the patient was infected (Central vs. South America). We expected, *ex ante*, to find

an earlier and higher rate of seroconversion in Central America due to the presence of different parasite lineages, i.e., *T. cruzi* type I predominating in Central America and *T. cruzi* type Non-I (II, V and VI) in South America.

We planned to undertake sensitivity analyses to determine the effect of restricting the analysis to only: (a) studies with low risk of selection bias (associated with sequence generation or allocation concealment), (b) studies with low risk of performance bias (associated with issues of blinding), and (c) studies with low risk of attrition bias (associated with completeness of data).

Finally, we used the GRADE profiler software package[24-26] in order to assign a level of evidence around the data extracted and to generate pooled estimates and their CIs, and to produce summary of findings tables.

Results

We identified 803 records through the database search and one additional record by contacting authors. After removing duplicates, 655 records were screened by title and abstract and 22 by full-text. Ultimately, 10 studies (four of them ongoing studies) met our inclusion criteria (**Figure 1. Study flow diagram**).

Figure 1. Study flow diagram

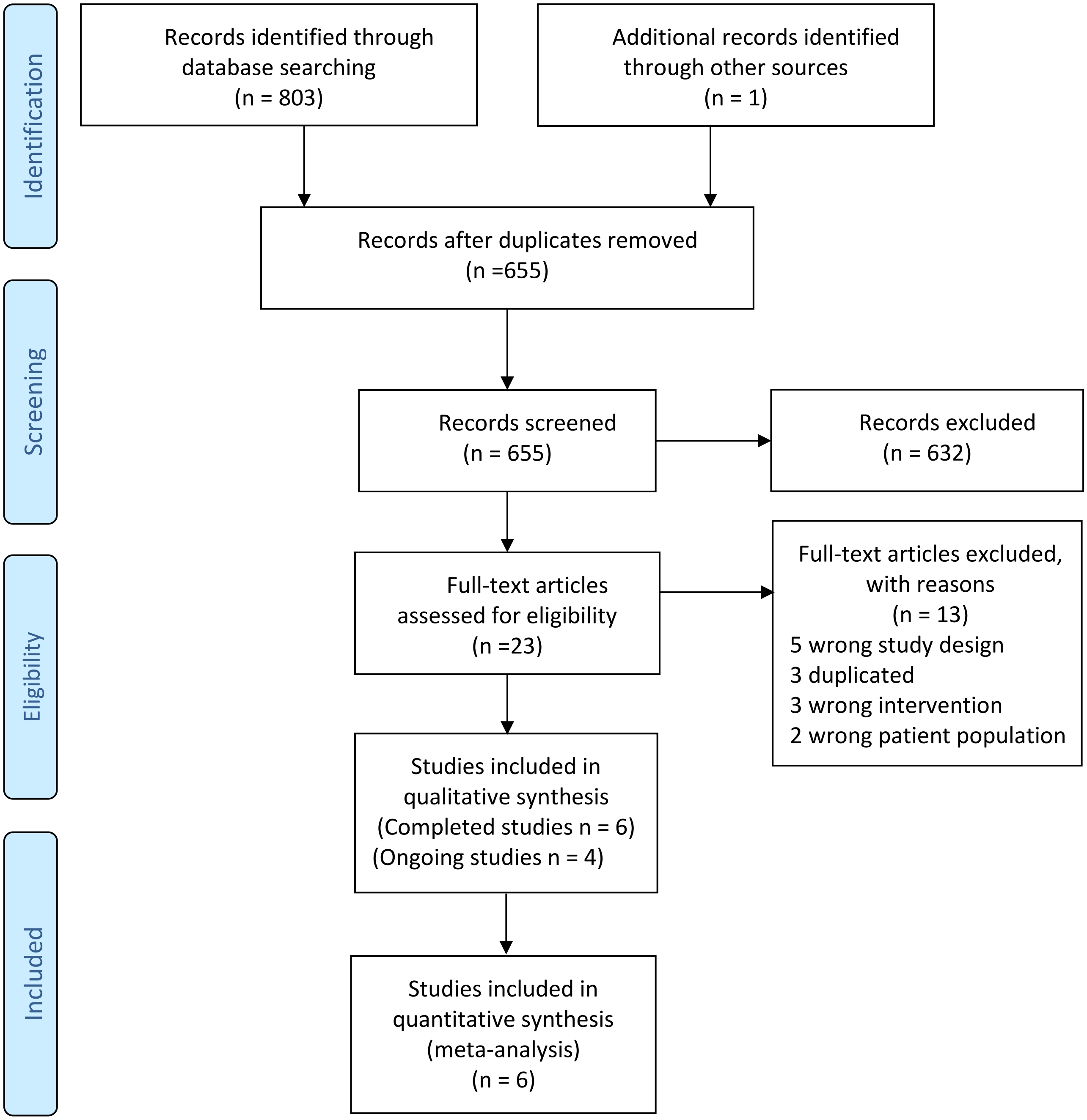

Included and ongoing studies

We included six completed RCTs[16, 17, 27-30] and identified four ongoing studies[18, 31-33] that met our inclusion criteria. See **Table 1 Main characteristics of included and ongoing studies** and **Appendix 3. Detailed description by study**). These studies directly compared fixed vs adjusted doses of BZN. Of the included studies, four were already published[17, 28-30] and details of the remaining two[16, 27] were obtained by personal communication with the authors.

Patients were recruited from Argentina, Bolivia, and Spain (two studies each), and from Brazil, Chile, Mexico, and Guatemala (one study each). Three studies used an adjusted dose[27, 28, 30] and three fixed doses[16, 17, 29]. One study was started before 1997 and used serology and xenodiagnosis[28], those that recruited patients from 1999 to 2018 used PCR for parasitological outcomes. Only one study provided long-term clinical outcomes, including cardiovascular mortality and progression of cardiomyopathy, but for comparison reasons we used a shorter follow-up period as in the majority of studies for non-clinical outcomes.[27] The mean sample size was 330 (minimum 77, maximum 910).

Although the ongoing studies are not included in the evidence synthesis, we have described them in detail (see **Table 1**) in order to explain how they might be able to answer our main question in the near future. Patients in these studies are being recruited from Argentina, Bolivia, Brazil, Colombia, and Spain. Only one of them will use an adjusted dose[31] and all of them will use PCR to detect presence of parasites.

Excluded studies

We excluded 12 studies, three of these were duplicate records. The reasons for excluding the other nine[34-42] are described in **Appendix 3: Excluded studies and reasons for exclusion**; seven of these were excluded due to a wrong study design.

Risk of bias in included studies

Only one study was considered of unclear risk of bias for the randomization domains[17]; two studies were considered of high risk for blinding assessment[17, 29] and one study for blinding of participants and personnel[29]; and two studies presented unclear risk for selective reporting[28, 43](see **Figure 2 risk of bias item for each included study,** and in **Appendix 4. Support for judgement of included studies by risk of bias item** and **Risk of bias graph across all included studies)**.

Figure 2 Risk of bias item for each included study

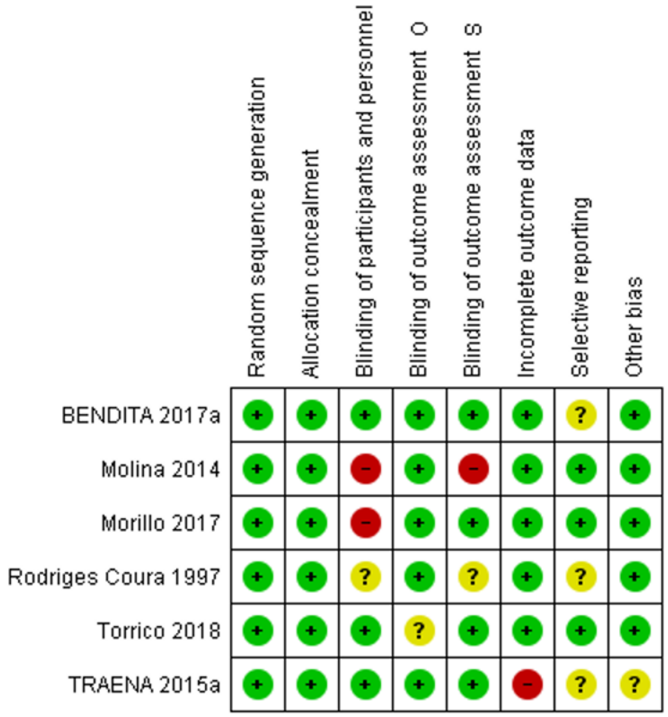

O: Objective outcomes; **S**: Subjective outcomes

Effects of interventions

In considering the main question of this review, we focused the results on comparisons that included both fixed and adjusted doses of BZN, for which we presented GRADE summary of finding tables.

Benznidazole fixed vs adjusted dose

There was no head-to-head study exploring this comparison but, based on inferences from indirect comparisons of BZN-treated patients versus placebo, using PCR at one year and safety outcomes (see BZN versus placebo below), we did not observe important differences between fixed and adjusted doses (see **Table 2 Summary of findings: Benznidazole fixed vs adjusted dose**).The certainty of evidence for these outcomes was downgraded one or two levels because of indirectness.

Benznidazole at different fixed doses

We identified the unpublished study BENDITA[16], which did not find important differences in positive PCR between BZN 150 vs 300 for 8 weeks (RR 1.00; IC95% 0.24-4.18), 150 vs 300 for 4 weeks (RR 1.20; IC95% 0.27-5.25), and 150 vs 300 for 2 weeks (RR 0.86; IC95% 0.21, 3.47). The authors also found no differences in AEs for the same comparisons: RR 0.88 (IC95% 0.56, 1.36), RR 1.05 (IC95% 0.65, 1.69), and RR 0.86 (IC 95% 0.49, 1.50) respectively. There were very few serious adverse events (SAEs) and drug discontinuations due to AEs, and no evidence of differences between groups. However, the study design was unpowered to detect differences between arms.

Benznidazole versus placebo

Efficacy was determined by positive serology, positive PCR, positive xenodiagnosis, mean reduction of antibody titer, mean reduction in PCR load, and clinical outcomes at the end of follow-up.

Positive serology: we identified one published study[28] without differences between groups (RR 1.00; IC95% 0.93-1.08) and one study[27] that favors treatment with BZN (RR 0.88; IC95% 0.84-0.93), both using an adjusted dose. The pooled RR was 0.94 (IC95% 0.82-1.08) and the certainty of evidence was considered low (see **Figure 3. Positive Serology at one year**). The TRAENA study[27] showed a RR of 0.65 (IC95% 0.58-0.63) at six years.

Figure 3. Positive serology at one year

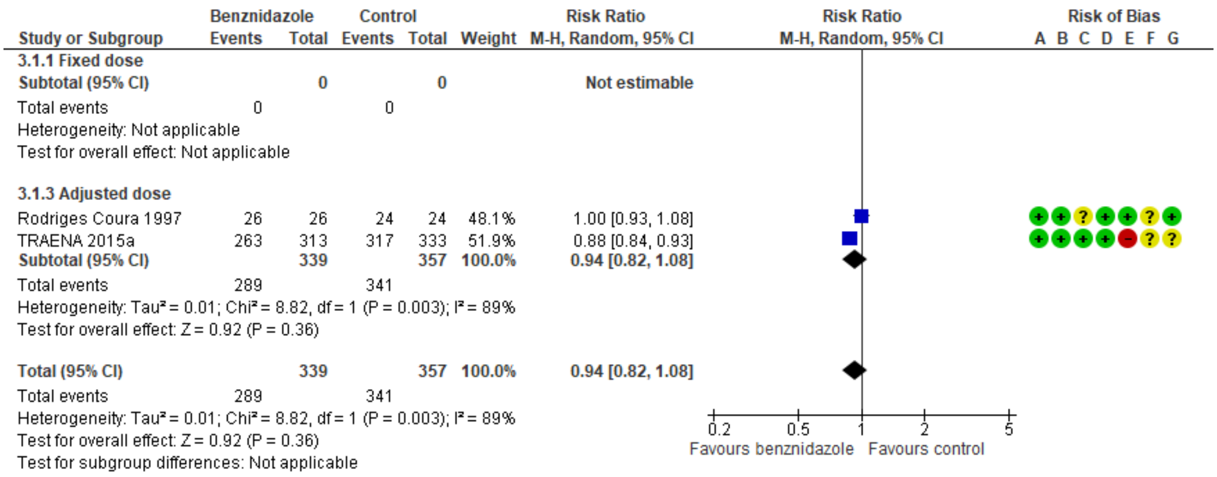

A) Random sequence generation (selection bias), (**B**) allocation concealment (selection bias), (**C**) blinding of participants and personnel (performance bias), (**D**) blinding of outcome assessment (detection bias), (**E**) incomplete outcome data (attrition bias), (**F**) selective reporting (reporting bias), (**G**) other bias

Positive PCR: both the fixed[17] and adjusted doses[16, 30] of BZN were effective at reducing positive PCR at one year against placebo (RR 0.20; IC95% 0.10-0.30) without differences in subgroups between the fixed dose of 300 mg/day for 14 to 56 days and the adjusted dose: RR 0.12

(IC95% 0.04-0.36) and RR 0.19 (IC95% 0.10-0.37), respectively. The significance of the test for subgroup differences was Chi² = 0.56, df = 1 (P = 0.45), I² = 0% (see **Figure 4. Positive PCR** and **Table 3. Summary of findings: Benznidazole versus placebo**).

Figure 4. Positive PCR at one year

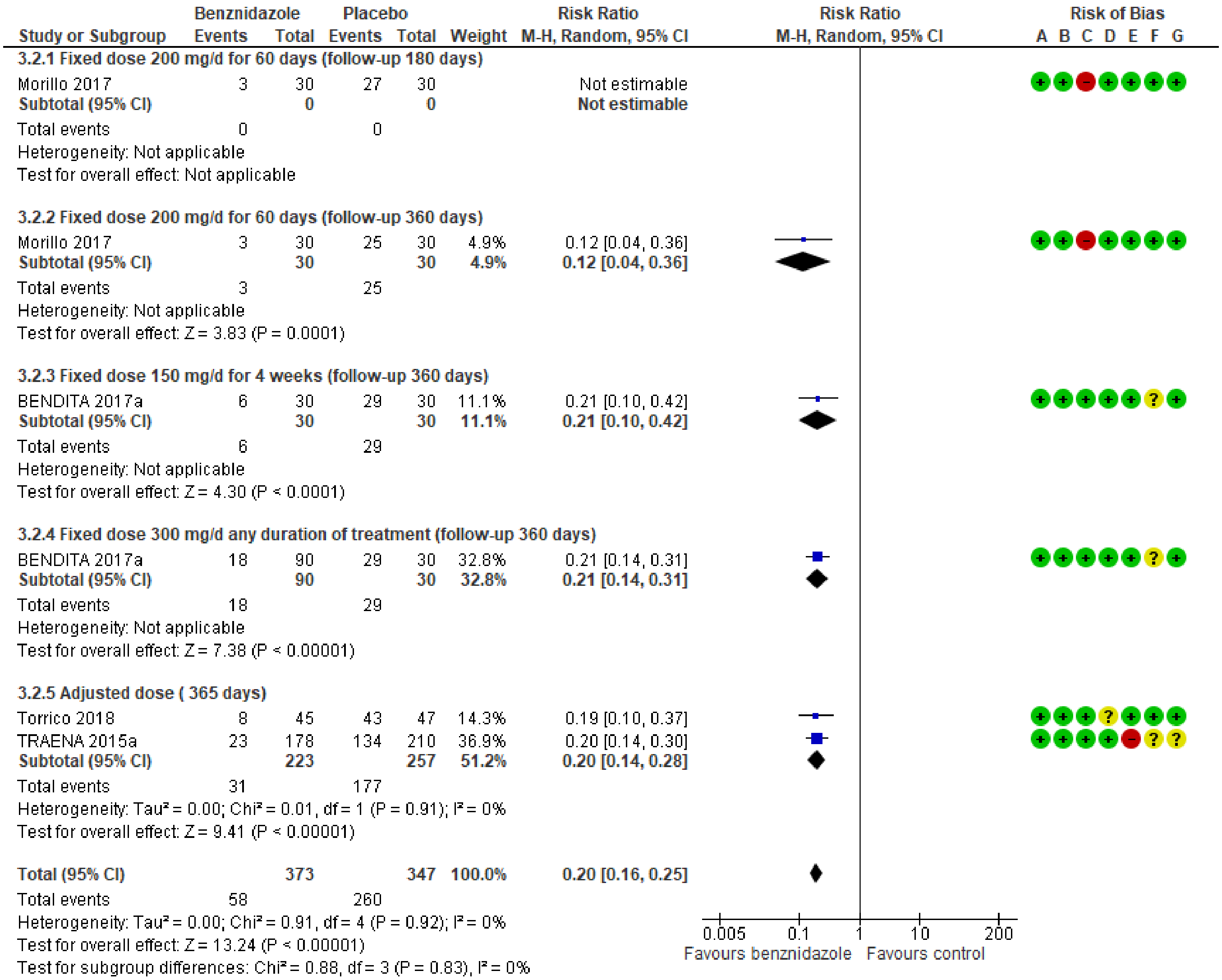

A) Random sequence generation (selection bias), **B**) allocation concealment (selection bias), **C**) blinding of participants and personnel (performance bias), **D)** blinding of outcome assessment objective outcomes (detection bias), **E**) incomplete outcome data (attrition bias), **F**) selective reporting (reporting bias), **H**) other bias

Positive xenodiagnosis: only one study provided data about an adjusted dose[28], favoring BZN (RR 0.12, 95% CI 0.04 to 0.36; participants = 60; studies = 1).

Mean reduction in PCR load (GMT one year): Two studies provided data about an adjusted dose[27, 30] and showed no significant difference: (MD -0.48; 95% CI -1.19, 0.23, participants = 480; studies = 2).

Mean reduction of antibody titer at one year: only one study (92 participants) provided data about an adjusted dose[30] and showed no difference with placebo (Conventional ELISA: MD 0.01, 95% CI -0.17 to 0.19 and AT CL−ELISA: MD 0.01, 95% CI -0.07 to 0.09) except when using the geometric mean measured by AT CL−ELISA (MD -0.57, 95% CI -1.08 to -0.06).

Clinical outcomes at the end of follow-up: one long-term study (n= 713) that used an adjusted dose of BZN vs. placebo provided data about these outcomes,[27] and showed a RR of 0.89 (95% CI 0.62 to 1.26) for progression of cardiomyopathy, RR 1.18, (95% CI 0.40 to 3.49) for cardiovascular mortality and RR 0.38 (95% CI 0.10 to 1.42) for pacemaker implantation, implantable cardioverters or severe arrhythmia with hemodynamic unbalance, and cardiac failure.

As expected, the frequency of AEs is higher with BZN (data not shown). Only three outcomes presented studies that used fixed or adjusted dose against placebo: drug discontinuation, peripheral neuropathy (considered SAEs), and mild rash (considered non-serious AEs).

Drug discontinuation: both the fixed and adjusted doses showed more drug discontinuation than placebo and we found no subgroup difference: Test for subgroup differences: $Chi^2 = 0.12$, df = 1

(P = 0.73), I² = 0% 9 (see **Figure 5 Drug discontinuation** and **Table 3. Summary of findings: Benznidazole versus placebo**.

Figure 5. Drug discontinuation

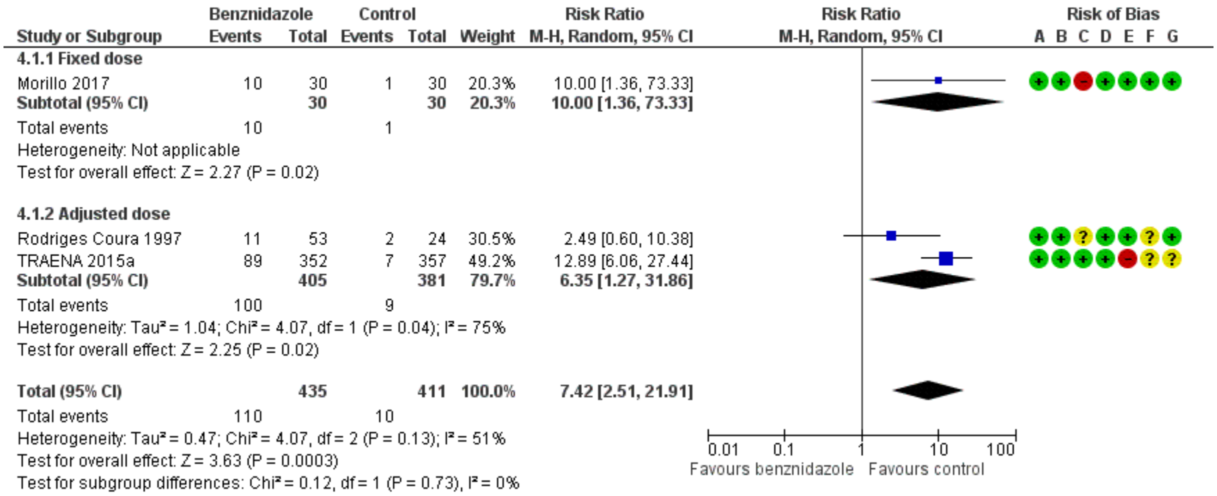

A) Random sequence generation (selection bias), **B**) allocation concealment (selection bias), **C**) blinding of participants and personnel (performance bias), **D)** blinding of outcome assessment objective outcomes (detection bias), **E**) incomplete outcome data (attrition bias), **F**) selective reporting (reporting bias), **H**) other bias

Peripheral neuropathy: we found no subgroup differences: Test for subgroup differences: Chi² = 1.50, df = 1 (P = 0.22), I² = 33.2%. See **Figure 6 Peripheral neuropathy and Table 3. Summary of findings: Benznidazole versus placebo**.

Figure 6. Peripheral neuropathy

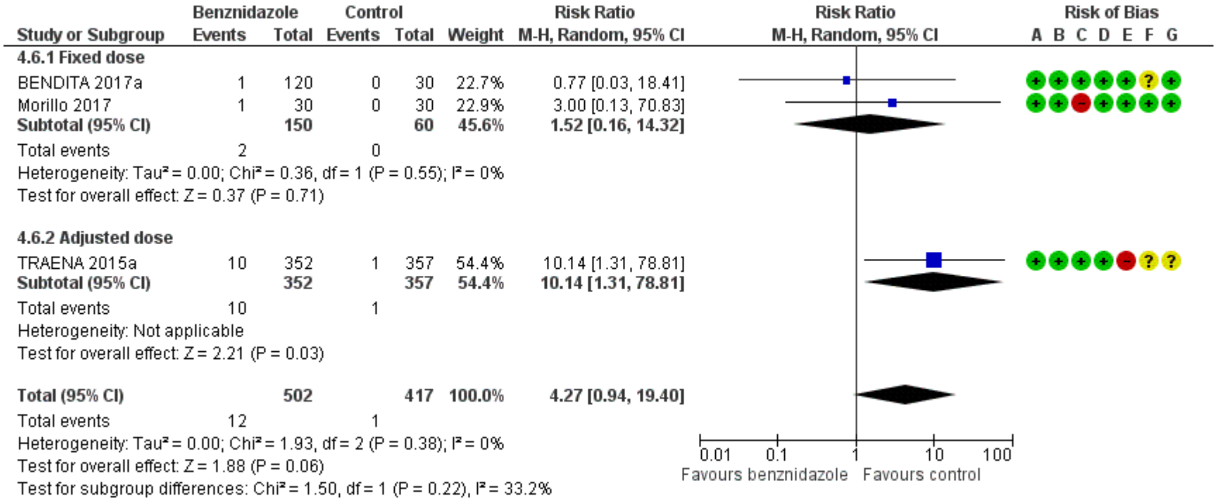

A) Random sequence generation (selection bias), **B**) allocation concealment (selection bias), **C**) blinding of participants and personnel (performance bias), **D)** blinding of outcome assessment objective outcomes (detection bias), **E**) incomplete outcome data (attrition bias), **F**) selective reporting (reporting bias), **H**) other bias

Mild rash: we found no subgroup difference: Test for subgroup differences: Chi² = 0.14, df = 1 (P = 0.93), I² = 0%) (see **Figure 7 Mild rash** and **Table 3. Summary of findings: Benznidazole versus placebo**).

Figure 7. Mild rash

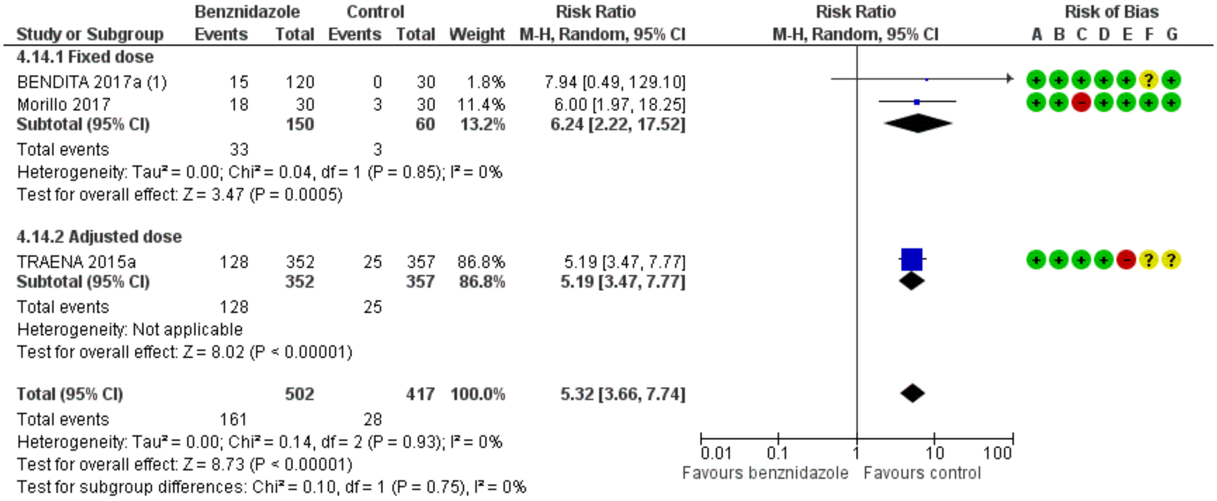

A) Random sequence generation (selection bias), **B**) allocation concealment (selection bias), **C**) blinding of participants and personnel (performance bias), **D)** blinding of outcome assessment objective outcomes (detection bias), **E**) incomplete outcome data (attrition bias), **F**) selective reporting (reporting bias), **H**) other bias

Benznidazole versus posaconazole

We identified two studies that used fixed-dose BZN versus posaconazole[17, 29] and considering the longest follow-up period, both favored BZN (RR 0.24, 95% CI 0.06 to 0.93; participants = 112; studies = 2; I^2 = 81%). Test for subgroup differences by follow-up: Chi^2 = 4.17, df = 1 (P = 0.04), I^2 = 76.0% due to higher effect in Morillo 2017 (follow-up 360 days)[17] than in Molina 2014 (follow-up 280 days)[29].

Subgroup analysis and sensitivity analysis

Due to the low number of included studies it was not possible to conduct the planned subgroup analyses, except for the fixed and adjusted doses related to the question of our review.

For the same reason, it was not possible to conduct the planned sensitivity analyses, restricting the analysis to only studies with low risk of selection bias (associated with sequence generation or allocation concealment). Morillo et al.[17] (unclear risk of bias for this domain) was the only fixed-dose study included in the subgroup comparison between fixed and adjusted doses of BZN.

All studies included in the subgroup comparison between fixed and adjusted doses of BZN were of low risk of performance bias (associated with issues of blinding) and low risk of attrition bias (associated with completeness of data) except for TRAENA[27]. We found consistency of results applying both fixed-effect and random-effects models and also using OR and RD.

Discussion

The only drugs with proven efficacy against Chagas disease are BZN and nifurtimox. BZN is used in children and adults and is registered for use in adjusted-dose schemes. However, some investigators have proposed the more flexible use of fixed dosing regardless of body weight. After searching all completed and ongoing RCTs involving BZN at any dose, we found no direct comparison between fixed and adjusted doses of BZN.

We only found one efficacy outcome (positive PCR) and three safety outcomes (drug discontinuation, peripheral neuropathy, and mild rash) that allowed the subgroup comparisons between fixed and adjusted doses of BZN. The low or null I^2 for all these subgroup comparisons suggest no important clinical, methodological, or statistical differences in the observed effects by type of dosing. Since these are inferences from indirect comparisons of BZN-treated patients versus placebo, the certainty of evidence for these outcomes was consequently downgraded one or two levels because of indirectness.

We found a high certainty of evidence for the direct comparisons between BZN versus placebo for the efficacy outcome (positive PCR) and for the three safety outcomes (drug discontinuation, peripheral neuropathy and mild rash). However, these four outcomes are considered as moderate certainty of the evidence for the comparison fixed vs adjusted dose of benznidazole after downgrading one level due to indirectness (indirect comparisons). The certainty of the evidence was considered to be very low for cardiovascular mortality and progression of cardiomyopathy due to significant uncertainty between ~~the surrogate outcome positive PCR~~ and these critical outcomes. The certainty of the evidence was considered low for any SAE due to uncertainty between this outcome and the surrogate outcome drug discontinuation. See **Table 2 Summary of**

findings: Benznidazole fixed vs adjusted dose (based on indirect comparisons) and **Table 3. Summary of findings: Benznidazole versus placebo** (for direct comparisons between BZN and placebo by type of dose).

We found six related systematic reviews that showed similar results in terms of the effect of interventions against placebo, however, none of them addressed our question concerning the comparison of fixed and adjusted doses of BZN [38, 39, 42, 44-46]Observational studies suggest that treatment could be better that no treatment even in the early phases of CCC[46]. Unfortunately, non-RCT studies used adjusted doses of BZN, not allowing the assessment of subgroup analysis by BZN scheme. Unlike other reviews, we only included RCTs to reduce the risk of bias, but, as was the case for the previous studies, we had to deal with differences in the populations studied, follow-up periods, diagnostic techniques, and sample size.

Demonstration that a fixed dose of BZN has a similar profile in relation to safety and efficacy would allow a review of the current guidelines and the recommendation of fixed doses, eliminating one barrier to treatment management, and improving compliance by patients and health workers.

The strengths of this systematic review of RCTs include the registration of its protocol, the complete literature search, the rigorous Cochrane methods used, the participation of most principal investigators of the RCTs included, and the inclusion of valuable unpublished data. All these factors make our study the most complete evidence synthesis currently available that addresses the comparative efficacy and safety of adjusted-dose BZN for *T. cruzi* seropositive adults mainly without CCC.

The population included in the trials is representative of the population of adults with chronic Chagas disease without cardiomyopathy, however important limitations need to be mentioned. First of all, the absence of direct comparisons between fixed and adjusted doses of BNZ, and the

assessments of critical outcomes should be noticed. Additionally, the paucity of studies prevented us from performing our planned subgroup and sensitivity analyses. The unpublished TRAENA study[27] was the only one that succeeded in assessing long-term clinical outcomes.

Individual patient data (IPD) meta-analysis could address these issues, for example by including cumulative dose assessments. Moreover, network IPD meta-analysis could formally enhance indirect comparisons.

Conclusion

Based on a low to very low certainty of evidence for critical clinical outcomes and a moderate certainty of evidence for important outcomes, fixed and adjusted doses of BZN might be considered equivalent in terms of efficacy and safety.

An IPD meta-analysis would allow us to conduct the planned subgroup analysis and meta-regressions, but given the absence of a direct comparison between fixed and adjusted doses of BZN, the only approach to gain in certainty of evidence to address the objective of the review would be an IPD network-meta-analysis - an approach which our research group is currently following.

Financial support

Independent grant from Drugs for Neglected Diseases *initiative* (DND*i*).

Acknowledgments

We want to acknowledge Daniel Comandé for his contribution to the search strategy.

Table 1. Main characteristics of included and ongoing studies

Short title	PI	Start/end years	N	Countries	Study status	Benznidazole dose	Comparison	Outcomes
Rodrigues C. 1997[28]	Rodrigues Coura, J.	<1997	77	Brazil	Completed	Adjusted	Nifurtimox/ Placebo	Serology / Xenodiagnoses
E1224[30]	Torrico, F.	2011/2	231	Bolivia	Completed	Adjusted	E1224 / Placebo	PCR
CHAGASAZOL [29]	Molina, C.	2010/1	79	Spain	Completed	Fixed	Posaconazole	PCR
STOP-CHAGAS[17]	Morillo, I.	2011/3	120	Argentina, Chile, México, Guatemala, Spain	Completed	Fixed	Posaconazole / Placebo	PCR
BENDITA[16]	Torrico , F.	2017/8	210	Bolivia	Completed Unpublished	Fixed	E1224 / Placebo	PCR
TRAENA[27]	Riarte, A.	1999/ 2015	910	Argentina	Completed Unpublished	Adjusted	Placebo	PCR Serology Cardiovascular Mortality, Progression
BETTY[32]	Buekens, P.	2019/	600	Argentina	Ongoing	Fixed	Benznidazole 300 mg	PCR
CHICAMOCHA 3[31, 47, 48]	Villar, JC.	2015/	500	Colombia	Ongoing	Adjusted	Nifurtimox/ Placebo	PCR
MULTIBENZ [18]	Molina, I.	2017/	240	Spain, Brazil Argentina, Colombia	Ongoing	Fixed	Benznidazole 150/400 mg	PCR
TESEO[33]	Almeida, IC.	2019	450	Bolivia	Ongoing	Fixed	Benznidazole 150/300 mg Nifurtimox 240/480 mg	RT-PCR

Table 2. Summary of findings: Benznidazole fixed vs adjusted dose

Outcome	Impact	№ of participants (studies)	Certainty
**Efficacy***			
**Positive PCR**	No important difference between fixed and adjusted dose (subgroup differences I² = 0%)	152 (2 RCTs)	⊕⊕⊕◯[1] MODERATE
**Cardiovascular mortality**	Based only in the surrogate outcome + PCR, differences between groups on critical outcomes are uncertain	152 (2 RCTs)	⊕◯◯◯[2] VERY LOW
**Progression of cardiomyopathy**		152 (2 RCTs)	⊕◯◯◯[2] VERY LOW
**Safety#**			
**Drug discontinuation**	No important difference between fixed and adjusted dose (Subgroup differences: I² = 0%)	846 (3 RCTs)	⊕⊕⊕◯[1] MODERATE
**Peripheral neuropathy**		769 (2 studies)	⊕⊕⊕◯[1] MODERATE
**Mild rash**		769 (2 studies)	⊕⊕⊕◯[1] MODERATE
**Any serious adverse events**	Based only in the surrogate outcome drug discontinuation, differences between groups on this critical outcome is uncertain	846 (3 RCTs)	⊕⊕◯◯[3] LOW

Refer to text for benznidazole versus placebo: Efficacy (PCR at one year*) and Safety#

[1]Downgraded one level due to serious indirectness, since these are inferences from subgroup analysis of comparisons between BZN and placebo.

[2]Downgraded two levels due to due to very serious indirectness (important uncertainty between the surrogate outcome + PCR and the critical outcomes cardiovascular mortality and progression of cardiomyopathy) and methodological limitations to answering this question.

[3]Downgraded one level due to serious indirectness, since there is uncertainty between surrogate outcome drug discontinuation and SAEs.

Table 3. Summary of findings: Benznidazole versus placebo

Outcome	Absolute effects (95% CI)		Relative effect (95% CI)	№ of participants (studies)	Certainty
	Risk with placebo: efficacy	Risk with benznidazole			
**Positive PCR**	883 per 1.000	**150 per 1.000 (88 to 265)**	**RR 0.20 (0.16 to 0.25)**	152 (2 RCTs)	⊕⊕⊕⊕ HIGH
- Fixed dose 300 mg/day for 14 to 56 days	833 per 1.000	**100 per 1.000 (33 to 300)**	**RR 0.12 (0.04 to 0.36)**	60 (1 RCT)	⊕⊕⊕⊕ HIGH
- Adjusted dose	915 per 1.000	**174 per 1.000 (91 to 339)**	**RR 0.19 (0.10 to 0.37)**	92 (1 RCT)	⊕⊕⊕⊕ HIGH
**Drug discontinuation**	24 per 1.000	**181 per 1.000 (61 to 533)**	**RR 7.42 (2.51 to 21.91)**	846 (3 RCTs)	⊕⊕⊕⊕ HIGH
- Fixed dose	33 per 1.000	**333 per 1.000 (45 to 1.000)**	**RR 10.00 (1.36 to 73.33)**	60 (1 RCT)	⊕⊕⊕⊕ HIGH
- Adjusted dose	24 per 1.000	**150 per 1.000 (30 to 753)**	**RR 6.35 (1.27 to 31.86)**	786 (2 RCTs)	⊕⊕⊕⊕ HIGH
**Peripheral neuropathy**	2 per 1000	**10 per 1000 (2 to 47)**	**RR 4.27 (0.94 to 19.40)**	919 (3 RCTs)	⊕⊕⊕⊕ HIGH
- Fixed dose	0 per 1000	**0 per 1000 (0 to 0)**	**RR 1.52 (0.16 to 14.32)**	210 (2 RCTs)	⊕⊕⊕⊕ HIGH
- Adjusted dose	3 per 1000	**28 per 1000 (4 to 221)**	**RR 10.14 (1.31 to 78.81)**	709 (1 RCT)	⊕⊕⊕⊕ HIGH
**Mild rash**	67 per 1000	**357 per 1000 (246 to 520)**	**RR 5.32 (3.66 to 7.74)**	919 (3 RCTs)	⊕⊕⊕⊕ HIGH
- Fixed dose	50 per 1000	**312 per 1000 (111 to 876)**	**RR 6.24 (2.22 to 17.52)**	210 (2 RCTs)	⊕⊕⊕⊕ HIGH
- Adjusted dose	70 per 1000	**363 per 1000 (243 to 544)**	**RR 5.19 (3.47 to 7.77)**	709 (1 RCT)	⊕⊕⊕⊕ HIGH

References

1. Stanaway JD, Roth G. The burden of Chagas disease: estimates and challenges. Glob Heart. 2015;10(3):139-44. Epub 2015/09/27. doi: 10.1016/j.gheart.2015.06.001. PubMed PMID: 26407508.
2. Global Burden of Disease (GBD) for Chagas disease 2017 [December 28th 2019]. Available from: https://vizhub.healthdata.org/gbd-compare/.
3. Lee BY, Bacon Km Fau - Bottazzi ME, Bottazzi Me Fau - Hotez PJ, Hotez PJ. Global economic burden of Chagas disease: a computational simulation model. (1474-4457 (Electronic)).
4. Hotez PJ, Bottazzi ME, Franco-Paredes C, Ault SK, Periago MR. The neglected tropical diseases of Latin America and the Caribbean: a review of disease burden and distribution and a roadmap for control and elimination. PLoS Neglected Tropical Diseases 2008;2(9):e300.
5. Bartsch SM, Bottazzi ME, Asti L, Strych U, Meymandi S, Falcon-Lezama JA, et al. Economic value of a therapeutic Chagas vaccine for indeterminate and Chagasic cardiomyopathy patients. Vaccine. 2019;37(28):3704-14. Epub 2019/05/21. doi: 10.1016/j.vaccine.2019.05.028. PubMed PMID: 31104883.
6. Coura JR, Vinas PA. Chagas disease: a new worldwide challenge. Nature 2010;465:S6-7.
7. Bern C, Montgomery SP. An estimate of the burden of Chagas disease in the United States. Clinical Infectious Diseases 2009;49(5):e52-4.
8. WHO. Control and prevention of Chagas disease in Europe, 2010. www.fac.org.ar/1/comites/chagas/Chagas_WHO_Technical%20Report_16_06_10.pdf (accessed 2 Nov2018).
9. Bocchi EA. Heart failure in South America. Curr Cardiol Rev. 2013;9(2):147-56. Epub 2013/04/20. doi: 10.2174/1573403x11309020007. PubMed PMID: 23597301; PubMed Central PMCID: PMCPMC3682398.
10. Moncayo A. Chagas disease: current epidemiological trends after the interruption of vectorial and transfusional transmission in the Southern Cone countries. Memorios do Instituto Oswaldo Cruz 2003;98(5):577-91.
11. Sosa-Estani S, Segura EL. Integrated control of Chagas disease for its elimination as public health problem--a review. Mem Inst Oswaldo Cruz. 2015;110(3):289-98. Epub 2015/05/21. doi: 10.1590/0074-02760140408. PubMed PMID: 25993503; PubMed Central PMCID: PMCPMC4489466.
12. Viotti R, Alarcon de Noya B, Araujo-Jorge T, Grijalva MJ, Guhl F, Lopez MC, et al. Towards a paradigm shift in the treatment of chronic Chagas disease. Antimicrob Agents Chemother. 2014;58(2):635-9. Epub 2013/11/20. doi: 10.1128/AAC.01662-13. PubMed PMID: 24247135; PubMed Central PMCID: PMCPMC3910900.
13. Villar JC. Commentary: control of Chagas' disease: let's put people before vectors. International Journal of Epidemiology 2001;30(4):894-5.
14. Pautas para la atención al paciente infectado con Trypanosoma cruzi (Enfermedad de Chagas). Buenos Aires: Ministerio de Salud, Argentina; 2015.
15. Pinazo MJ, Guerrero L Fau - Posada E, Posada E Fau - Rodriguez E, Rodriguez E Fau - Soy D, Soy D Fau - Gascon J, Gascon J. Benznidazole-related adverse drug reactions and their relationship to serum drug concentrations in patients with chronic chagas disease. (1098-6596 (Electronic)).
16. (03378661) B. BENDITA BEnznidazole New Doses Improved Treatment and Associations. 2017.
17. Morillo CA, Waskin H, Sosa-Estani S, Del Carmen Bangher M, Cuneo C, Milesi R, et al. Benznidazole and Posaconazole in Eliminating Parasites in Asymptomatic T. Cruzi Carriers: The STOP-CHAGAS Trial. J Am Coll Cardiol. 2017;69(8):939-47. doi: 10.1016/j.jacc.2016.12.023. PubMed PMID: 28231946.
18. (03191162) M. Evaluation of Different Benznidazole Regimens for the Treatment of Chronic Chagas Disease. https://clinicaltrialsgov/ct2/show/NCT03191162. 2018.
19. Higgins J, Thomas Je. Cochrane Handbook for Systematic Reviews of Interventions Version 6 [updated August 2019]. 2019. Available from: Available from https://training.cochrane.org/handbook/current.
20. Liberati A, Altman DG, Tetzlaff J, Mulrow C, Gotzsche PC, Ioannidis JP, et al. The PRISMA statement for reporting systematic reviews and meta-analyses of studies that evaluate health care interventions: explanation and elaboration. PLoS Med. 2009;6(7):e1000100. Epub 2009/07/22. doi: 10.1371/journal.pmed.1000100. PubMed PMID: 19621070.
21. Moher D, Liberati A, Tetzlaff J, Altman DG. Preferred reporting items for systematic reviews and meta-analyses: the PRISMA statement. PLoS Med. 2009;6(7):e1000097. Epub 2009/07/22. doi: 10.1371/journal.pmed.1000097. PubMed PMID: 19621072.
22. Higgins J, Savović J, Page M, Elbers R, Sterne J. Chapter 8: Assessing risk of bias in a randomized trial. 2018. In: Cochrane Handbook for Systematic Reviews of Interventions Version 6 [updated August 2019]
[Internet]. Cochrane. Available from: Available from www.cochrane-handbook.org.
23. Review Manager (RevMan). [Computer program] Version 53 Copenhagen: The Nordic Cochrane Centre, The Cochrane Collaboration; 2014.
24. Guyatt G, Oxman AD, Akl EA, Kunz R, Vist G, Brozek J, et al. GRADE guidelines: 1. Introduction-GRADE evidence profiles and summary of findings tables. J Clin Epidemiol. 2011;64(4):383-94. Epub 2011/01/05. doi: S0895-4356(10)00330-6 [pii]
10.1016/j.jclinepi.2010.04.026. PubMed PMID: 21195583.
25. Schünemann H, Vist G, Higgins J, Santesso N, Deeks J, Glasziou P, et al. Chapter 15: Interpreting results and drawing conclusions. 2018. In: Cochrane Handbook for Systematic Reviews of Interventions Version 6 [updated September 2018]
[Internet]. Cochrane. Available from: Available from www.cochrane-handbook.org.
26. Schünemann H, Brożek J, Guyatt G, Oxman A, (editors). Handbook for grading the quality of evidence and the strength of recommendations using the GRADE approach [updated October 2013]. 2013. Available from: Available from http://gdt.guidelinedevelopment.org/app/index.html.
27. (02386358) T. Etiologic Treatment With Benznidazole in Adult Patients With Chronic Chagas Disease. A Randomized Clinical Trial. 2015.
28. Coura JR, de Abreu LL, Willcox HP, Petana W. [Comparative controlled study on the use of benznidazole, nifurtimox and placebo, in the chronic form of Chagas' disease, in a field area with interrupted transmission. I. Preliminary evaluation]. Revista da Sociedade Brasileira de Medicina Tropical. 1997;30(2):139-44.
29. Molina I, Gomez i Prat J, Salvador F, Trevino B, Sulleiro E, Serre N, et al. Randomized trial of posaconazole and benznidazole for chronic Chagas' disease. N Engl J Med. 2014;370(20):1899-908. doi: 10.1056/NEJMoa1313122. PubMed PMID: 24827034.
30. Torrico F, Gascon J, Ortiz L, Alonso-Vega C, Pinazo MJ, Schijman A, et al. Treatment of adult chronic indeterminate Chagas disease with benznidazole and three E1224 dosing regimens: a proof-of-concept, randomised, placebo-controlled trial. Lancet Infect Dis. 2018;18(4):419-30. doi: 10.1016/S1473-3099(17)30538-8. PubMed PMID: 29352704.
31. (02369978) C. CHICAMOCHA 3 - Equivalence of Usual Interventions for Trypanosomiasis (EQUITY). https://clinicaltrialsgov/ct2/show/NCT02369978. 2015.

32. (03672487) B. Short-course Benznidazole Treatment to Reduce Trypanosoma Cruzi Parasitic Load in Women of Reproductive Age. https://clinicaltrialsgov/ct2/show/NCT03672487. 2018.
33. (03981523) T. New Therapies and Biomarkers for Chagas Infection. https://clinicaltrialsgov/show/NCT03981523. 2019. PubMed PMID: CN-01945435.
34. Álvarez MG, Hernández Y, Bertocchi G, Fernández M, Lococo B, Ramírez JC, et al. New scheme of intermittent benznidazole administration in patients chronically infected with Trypanosoma cruzi: A pilot short-term follow-up study with adult patients. Antimicrobial Agents and Chemotherapy. 2016;60(2):833-7. doi: 10.1128/AAC.00745-15.
35. Andrade AL, Martelli CM, Oliveira RM, Silva SA, Aires AI, Soussumi LM, et al. Short report: benznidazole efficacy among Trypanosoma cruzi-infected adolescents after a six-year follow-up. American journal of tropical medicine and hygiene. 2004;71(5):594-7. PubMed PMID: CN-00560425.
36. Apt W, Arribada A, Arab F, Ugarte JM, Luksic I, Solé C. Clinical trial of benznidazole and an immunopotentiator against Chagas disease in Chile. Transactions of the Royal Society of Tropical Medicine and Hygiene. 1986;80(6):1010.
37. Molina I, Prat JGI, Salvador F, Treviño B, Serre N, Sulleiro E, et al. Phase IIb clinical trial with posaconazol and benznidazol for chronic Chagas disease. Tropical Medicine and International Health. 2013;18:60. doi: 10.1111/tmi.12162.
38. Müller Kratz J, Garcia Bournissen F, Forsyth CJ, Sosa-Estani S. Clinical and pharmacological profile of benznidazole for treatment of Chagas disease. Expert Review of Clinical Pharmacology. 2018;11(10):943-57. doi: 10.1080/17512433.2018.1509704.
39. Pérez-Molina JA, Pérez-Ayala A, Moreno S, Fernández-González MCMC, Zamora J, López-Velez R. Use of benznidazole to treat chronic Chagas' disease: A systematic review with a meta-analysis. Journal of Antimicrobial Chemotherapy. 2009;64(6):1139-47. doi: 10.1093/jac/dkp357.
40. Sguassero Y, Cuesta CB, Roberts KN, Hicks E, Comandé D, Ciapponi A, et al. Course of chronic Trypanosoma cruzi infection after treatment based on parasitological and serological tests: A systematic review of follow-up studies. PLoS ONE. 2015;10(10):e0139363. doi: 10.1371/journal.pone.0139363.
41. Sosa-Estani S, Armenti A, Araujo G, Viotti R, Lococo B, Ruiz Vera B, et al. Treatment of Chagas disease with benznidazole and thioctic acid. Medicina. 2004;64(1):1-6. PubMed PMID: CN-00480924.
42. Villar JC, Perez JG, Cortes OL, Riarte A, Pepper M, Marin-Neto JA, et al. Trypanocidal drugs for chronic asymptomatic Trypanosoma cruzi infection. Cochrane Database of Systematic Reviews. 2014;(5). doi: 10.1002/14651858.CD003463.pub2. PubMed PMID: CD003463.
43. Prado N, Hernández Y, De Rissio AM, Esteve M, Riarte A. La seguridad en un ensayo clínico aleatorizado (ECA) - Estudio TRAENA - en pacientes adultos con Enfermedad de Chagas. 2008 2008. Report No.
44. Bern C, Montgomery SP, Herwaldt BL, Rassi A, Marin-Neto JA, Dantas RO, et al. Evaluation and treatment of chagas disease in the United States: a systematic review. JAMA. 2007;298(18):2171-81. doi: 10.1001/jama.298.18.2171.
45. Echeverria LE, Morillo CA. American Trypanosomiasis (Chagas Disease). Infect Dis Clin North Am. 2019;33(1):119-34. Epub 2019/02/05. doi: 10.1016/j.idc.2018.10.015. PubMed PMID: 30712757.
46. Reyes PPA, Vallejo M. Trypanocidal drugs for late stage, symptomatic Chagas disease (Trypanosoma cruzi infection). Cochrane Database of Systematic Reviews. 2005;(4):CD004102. doi: 10.1002/14651858.CD004102.pub2.
47. Villar JC, Herrera VM, Perez Carreno JG, Vaquiro Herrera E, Castellanos Dominguez YZ, Vasquez SM, et al. Nifurtimox versus benznidazole or placebo for asymptomatic Trypanosoma cruzi infection (Equivalence of Usual Interventions for Trypanosomiasis - EQUITY): study protocol for a randomised controlled trial. Trials. 2019;20(1):431. Epub 2019/07/17. doi: 10.1186/s13063-019-3423-3. PubMed PMID: 31307503; PubMed Central PMCID: PMCPMC6631895.
48. Villar JC, Herrera VM, Carreno JGP, Herrera EV, Dominguez YZC, Vasquez SM, et al. Correction to: Nifurtimox versus benznidazole or placebo for asymptomatic Trypanosoma cruzi infection (Equivalence of Usual Interventions for Trypanosomiasis - EQUITY): study protocol for a randomised controlled trial. Trials. 2019;20(1):516. Epub 2019/08/21. doi: 10.1186/s13063-019-3630-y. PubMed PMID: 31429793; PubMed Central PMCID: PMCPMC6701118.

APPENDICES

- **Appendix 1: Search strategy**
- **Appendix 2: Risk of bias assessment tool**
- **Appendix 3: Detailed description of included and ongoing studies** and **Excluded studies and reasons for exclusion**
- **Appendix 4: Support for judgement of included studies by risk of bias item** and **Risk of bias graph across all included studies**
- **Appendix 5: PRISMA Checklist**

Manuscript tracked changes

Click here to access/download;Manuscript;Fixed vs adjusted BZN. SR MA Tracked changes 2.pdf

Fixed vs adjusted-dose benznidazole for adults with chronic Chagas disease without cardiomyopathy: A systematic review and meta-analysis

Ciapponi Agustín[1*], Barreira Fabiana[2,3], Perelli Lucas[1], Bardach Ariel[1], Gascón Joaquim[4], Molina Israel[5], Morillo Carlos[6], Prado Nilda[7], Riarte Adelina[7], Torrico Faustino[8], Ribeiro Isabela[9], Villar Juan Carlos[10], Sosa-Estani Sergio[2, 11]*.

1 Centro Cochrane Argentino-Instituto de Efectividad Clínica y Sanitaria (IECS-CONICET), Buenos Aires, Argentina.

2 Drugs for Neglected Diseases *initiative* (DND*i*), Río de Janeiro, Brazil.

3 Epidemiology and Public Health Research Center, CONICET, Buenos Aires, Argentina.

4 Hospital Clínic de Barcelona, Barcelona, España.

5 Hospital Universitari Vall d'Hebron Research Institute, Barcelona, España.

6 McMaster University, Population Health Research Institute, Hamilton, Canada

7 Instituto Nacional de Parasitología Dr. M Fatala Chaben, Buenos Aires, Argentina.

8 Universidad Mayor de San Simón, Cochabamba, Bolivia.

9 Drugs for Neglected Diseases *initiative* (DND*i*), Geneva, Switzerland.

10 Grupo de Cardiología Preventiva, Facultad de Ciencias de la Salud, Universidad Autónoma de Bucaramanga, Bucaramanga, Colombia

11 Centro de Investigaciones Epidemiológicas y Salud Pública (CIESP-IECS). CONICET, Buenos Aires, Argentina

***** Contact authors

Abstract

Background

Chagas disease is a neglected disease that remains a public health threat, particularly in Latin America. The most important treatment options are nitroimidazole derivatives, such as nifurtimox and benznidazole (BZN). Some studies suggest that for adults seropositive to *T. cruzi* but without clinically evident chronic Chagas cardiomyopathy (CCC), a simple fixed-dose scheme of BZN could be equivalent to a weight-adjusted dose.

Objectives

To determine the comparative efficacy and safety of a fixed dose of BZN with an adjusted dose for *T. cruzi* seropositive adults without CCC.

Methods

We performed a systematic review and meta-analysis following Cochrane methods, and reported according to the PRISMA statement. We included randomized controlled trials (RCTs) allocating participants to fixed and/or adjusted doses of BZN for *T. cruzi* seropositive adults without CCC. We searched (December 2019) Cochrane, MEDLINE, EMBASE, LILACS, Clinicaltrials.gov, and International Clinical Trials Registry Platform (ICTRP), and contacted Chagas experts. Selection, data extraction, and risk of bias assessment, using the Cochrane tool, were performed independently by pairs of reviewers. Discrepancies were solved by consensus within the team. Primary outcomes were parasite-related outcomes and efficacy or patient-related safety outcomes. We conducted a meta-analysis using RevMan 5.3 software and used GRADE summary of finding tables to present the certainty of evidence by outcome.

Main results

We identified 655 records through our search strategy and 10 studies (four of them ongoing) met our inclusion criteria. We did not find any study directly comparing fixed vs adjusted doses of BZN, however, some outcomes allowed subgroup comparisons between fixed and adjusted doses of BZN against placebo. Moderate-certainty evidence suggests no important subgroup differences for positive PCR at one year and for three safety outcomes (drug discontinuation, peripheral neuropathy, and mild rash). The same effect was observed for any serious adverse events (low-certainty evidence). All subgroups showed similar effects (I^2 0% for all these subgroup comparisons but 32% for peripheral neuropathy), supporting the equivalence of BZN schemes.

Conclusions

There is no direct evidence comparing fixed and adjusted doses of BZN. Based on low to very low certainty of evidence for critical clinical outcomes and moderate certainty of evidence for important outcomes, fixed and adjusted doses may be equivalent in terms of safety and efficacy. An individual patient data network meta-analysis could better address this issue.

Registration

The study protocol was registered in PROSPERO (CRD42019120905).

Author Summary

Chagas disease is a major public health problem that requires, among other control interventions, an optimal trypanocidal therapy that achieves the best possible compliance to cure active infection, mainly in children and young populations, women before they become pregnant to prevent congenital transmission, and chronic populations with a risk of progression to cardiomyopathy who are currently not being treated.

Some studies suggest that a simple fixed-dose scheme of benznidazole could be equivalent to the dose adjusted by weight for the treatment of adults seropositive to *T. cruzi* without clinically evident chronic Chagas cardiomyopathy. To confirm or reject this potential equivalence of schemes, we conducted a rigorous systematic review and meta-analysis of randomized controlled trials by reviewing and analyzing the totality of available literature on the subject. Although we did not find direct evidence addressing this question, it appears that an adjusted dose is probably equivalent in terms of important safety and efficacy outcomes, while the effect on critical outcomes is uncertain. Since we did not find any ongoing study comparing fixed versus adjusted doses of benznidazole, we are conducting an individual patient data network meta-analysis to address this question.

Introduction

Chagas disease (CD), also known as human American trypanosomiasis, is a condition resulting from infection by the parasite *Trypanosoma cruzi* (*T. cruzi*). Chagas disease remains a major public health problem; between five and 18 million people are currently infected and the disease is estimated to cause more than 10,000 deaths annually.[1] Globally, the annual burden is $627.5 million in health-care costs and 232.000[2][2] to 806,170 disability-adjusted life years (DALYs).[3] The Latin American region bears most of the burden of Chagas disease, accounting for at least 206,000[2][2] to 662,000 DALYs lost.[4] A study investigating the economic value of a therapeutic Chagas vaccine found that when administering standard of care benznidazole (BZN) to 1000 indeterminate patients, 148 discontinued treatment and 219 progressed to chronic disease, resulting in 119 Chagas-related deaths and 2293 DALYs, costing $18.9 million in lifetime societal costs.[5] Population migration dynamics combined with the increased risk of mothers infecting their unborn children and the increased risk of infection from blood or solid-organ donations, means that Chagas disease has become a global problem.[6][6] The number of infected individuals has been estimated at 300,000 in the USA,[7][7] and 80,000 in Europe.[8] Primary acute *T.*[8] Primary acute *T.* *cruzi* infection is seldom clinically evident, given its lack of defining features; it is often asymptomatic or resembles a common viral illness, although more serious outcomes such as myocarditis or meningoencephalitis are possible. During this period, *T. cruzi* trypomastigotes are directly observable in the bloodstream. After this comes an indeterminate chronic phase, during which *T. cruzi* lodges in organ tissue in amastigote form, inducing a specific immune response. While most remain asymptomatic, 30-40% of patients progress to an advanced disease stage, usually years to decades after the initial infection. The advanced chronic phase frequently involves damage to the conduction system of the heart and the myocardium, which can result in heart failure

and sudden death. In the Americas, myocarditis secondary to CD is the most common form of nonischemic cardiomyopathy.[9] In other cases, CD produces gastrointestinal disorders (especially megaesophagus and megacolon), or disorders of the central or peripheral nervous system, particularly in immunocompromised patients. Serology is used to confirm a diagnosis of chronic *T. cruzi* infection and polymerase chain reaction (PCR) contributes to that diagnosis. For years, host-based control was considered a difficult goal to achieve and consequently, in the 1990s, public health authority efforts were focused on primary prevention, *Triatoma infestans*-based control, and control of blood donors to prevent infection of individuals at risk.[10][10] In recent years, significant progress has been made in the fight against triatomines, which, added to the controls implemented by blood banks, has drastically reduced *T. cruzi* infections by vectors and transfusions. Interest in host-based control, that is treating chronically infected individuals with trypanocidal therapy, has increased.[11, 12] Additionally, the focus on vector-based control has left the already infected population without interventions that are potentially preventive of CCC.[13][13] Two nitroimidazolic derivatives, BZN and nifurtimox, are the only approved trypanocidal options currently used, with no important differences in their relative efficacy, adverse effects (AEs) and cost.[14] The usual recommended dose of BZN is 5 to 7 mg/kg/day orally (5–10 mg/kg for children up to 12 years old) divided into two or three times daily, for 60 days for adults. The most frequently reported side effects are skin reactions and neuropathy, which commonly result in interruption of treatment.[15]

Recently, several studies[16-18] suggested that the use of a simpler fixed dose of BZN may be equivalent to an adjusted dose in terms of effectiveness, simplifying its administration and enhancing compliance. In order to compare the efficacy and safety of both schemes for *T. cruzi* seropositive adults without CCC, we have systematically searched and extracted data from eligible

studies comparing relevant clinical, parasitological, and biochemical outcomes for seropositive adults exposed to fixed and/or adjusted doses of BZN.

Methods

We conducted a systematic review and meta-analysis following Cochrane methods,[19][19] and the PRISMA statement for reporting.[20, 21] The study protocol was registered in PROSPERO (CRD42019120905).

Eligibility criteria

Randomized controlled trials (RCTs) allocating adults with asymptomatic chronic Chagas disease to fixed and/or adjusted doses BZN vs placebo or other trypanocidal treatments were included. RCTs had to include people with chronic *T. cruzi* infection, diagnosed with positive serology by at least two of the following techniques: ELISA, indirect hemagglutination (IHA), or indirect immunofluorescence (IIF), mainly without clinically evident (i.e. symptomatic) CCC. Important safety and efficacy outcomes, such as positive serology or PCR, any adverse events (AEs) and serious adverse events (SAEs) and critical patient (clinical) related outcomes, such as all-cause mortality or significant progression of CCC, were analyzed.

Search strategy

We searched (December 2019) the following databases: Cochrane Database of Systematic Reviews (CDSR), Cochrane Central Register of Controlled Trials (CENTRAL), Database of Abstracts of Reviews of Effects (DARE), MEDLINE, EMBASE, LILACS, Clinicaltrials.gov, and the WHO International Clinical Trials Registry Platform (ICTRP).

The basic search strategy included the following terms: (Chagas Disease [Mesh] OR Chagas[tiab] OR Trypanosom*[tiab] OR Cruzi[tiab] OR T.Cruzi[tiab]) AND (Benznidazole[Supplementary Concept] OR benznidazol*[tiab] OR Radanil[tiab] OR Rochagan[tiab] OR N-bencil-2-acetamide[tiab]).

The search strategy was adapted to each database (See **Appendix 1 Search strategy**).

No language limitations or publication date restrictions were applied. For studies with multiple publications, we decided how to best use the data on a case-by-case basis through discussion with the principal investigators.

Additional searches included a Google search (the first 100 hits, in order of relevance, when typing Chagas benznidazol), handsearching of reference lists of systematic reviews and eligible studies retrieved with the electronic search, and verbal feedback from experts in the field.

Screening and data extraction

Selection, data extraction, and risk of bias assessment were performed independently by pairs of reviewers from the research team. Discrepancies were solved by consensus within the team. All the study selection phases were completed using COVIDENCE®, a web-based platform designed for the systematic review process. Authors of articles were contacted when necessary to obtain missing or supplementary information.

A pre-designed general data extraction form was used after pilot testing.

We extracted the source of study report, study location and setting, population, disease definition of chronic *T. cruzi* infection, diagnostic tests used (number and type of laboratory tests used), quality control measures, BZN and other treatment schedules, and follow-up measures.

Risk of bias (quality) assessment

Pairs of independent reviewers assessed the risk of bias (quality) using the Cochrane Collaboration tool.[22] See details in **Appendix 2 Risk of bias assessment**. Discrepancies were solved by consensus within the team.

Data synthesis

We conducted a traditional aggregate meta-analysis by using the Review Manager 5.3 software package.[23] Pooled effect estimates and their 95% confidence intervals (CI), when appropriate, were generated using a random-effects model. We reported risk ratios (RR) for dichotomous outcomes (e.g. positive serology after treatment), the Peto odds ratio (OR) for very infrequent outcomes, and the mean difference (MD) for continuous data such as antibody titers. For dichotomous data, we used the Mantel-Haenszel method, and for continuous data, we used the inverse variance method.

We described statistical heterogeneity of intervention effects by calculating the I^2 statistic and we interpreted 0-30% as not important, 30-60% as moderate heterogeneity, and more than 60% as substantial heterogeneity. Since we assume that clinical heterogeneity is very likely to impact our review results, given the nature of the interventions included, we primarily reported the random-effects model results, however, we also applied the fixed effect model as a sensitivity analysis. We calculated all overall effects using inverse variance methods.

Where necessary, we contacted the corresponding authors of included studies up to three times to supply any unreported data.

We planned subgroup analyses (see protocol) by age of participants at time of treatment (young adults vs. older population), type of serological test (conventional serology vs. non-conventional serology), time of treatment and testing (less than ten years vs. equal or more than ten years), and region where the patient was infected (Central vs. South America). We expected, *ex ante*, to find an earlier and higher rate of seroconversion in Central America due to the presence of different parasite lineages, i.e., *T. cruzi* type I predominating in Central America and *T. cruzi* type Non-I (II, V and VI) in South America.

We planned to undertake sensitivity analyses to determine the effect of restricting the analysis to only: (a) studies with low risk of selection bias (associated with sequence generation or allocation concealment), (b) studies with low risk of performance bias (associated with issues of blinding), and (c) studies with low risk of attrition bias (associated with completeness of data).

Finally, we used the GRADE profiler software package[24-26] in order to assign a level of evidence around the data extracted and to generate pooled estimates and their CIs, and to produce summary of findings tables.

Results

We identified 803 records through the database search and one additional record by contacting authors. After removing duplicates, 655 records were screened by title and abstract and 22 by full-text. Ultimately, 10 studies (four of them ongoing studies) met our inclusion criteria (**Figure 1. Study flow diagram**).

Con formato: Izquierda, Derecha: 0 cm, Interlineado: sencillo

Figure 1. Study flow diagram

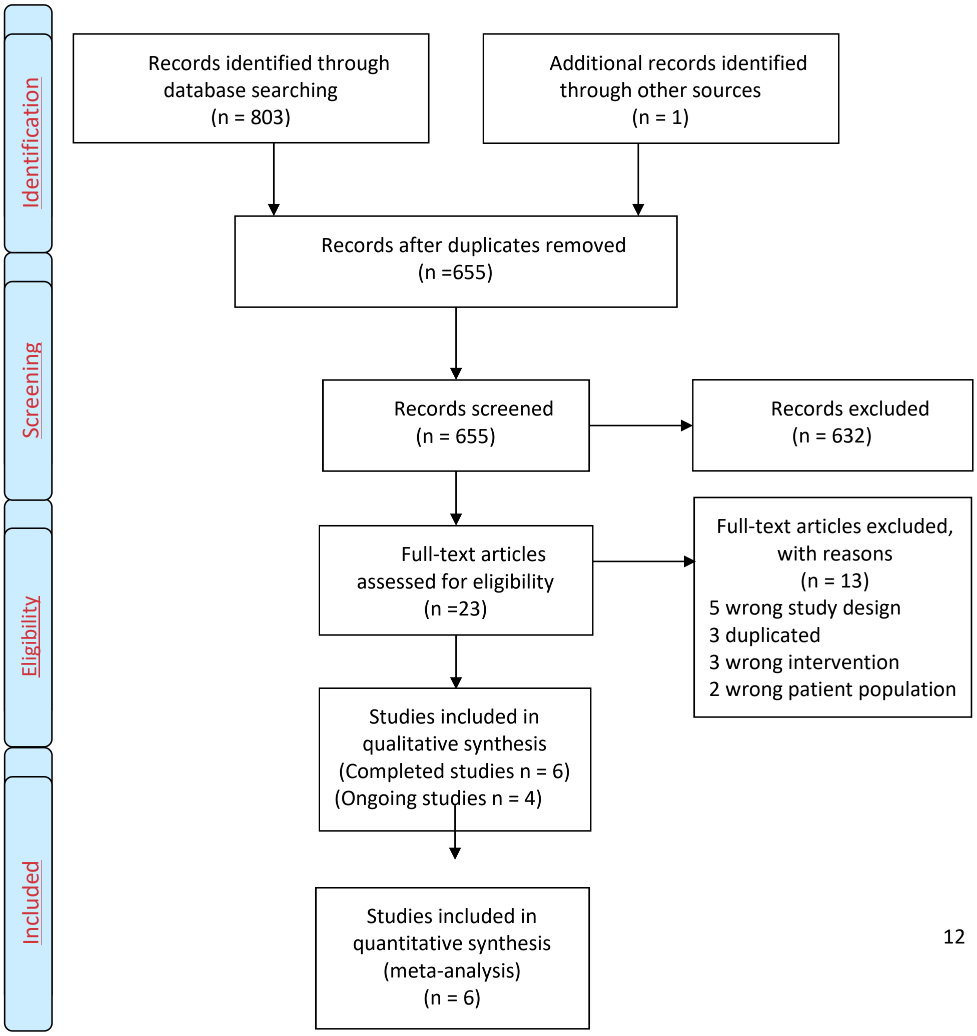

Included and ongoing studies

We included six completed RCTs[16, 17, , 17, 27-30] and identified four ongoing studies[18, 31-33] that met our inclusion criteria. See **Table 1 Main characteristics of included and ongoing studies** and **Appendix 3. Detailed description by study**). These studies directly compared fixed vs adjusted doses of BZN. Of the included studies, four were already published[17, 29, 30, 34] and details of the remaining two[16, 27] were obtained by personal communication with the authors.

Patients were recruited from Argentina, Bolivia, and Spain (two studies each), and from Brazil, Chile, Mexico, and Guatemala (one study each). Three studies used an adjusted dose[27, 28, 30], 30, 34] and three fixed doses[16, 17, , 17, 29]. One study was started before 1997 and used serology and xenodiagnosis[28], those that recruited patients from 1999 to 2018 used PCR for parasitological outcomes.[34], those that recruited patients from 1999 to 2018 used PCR for parasitological outcomes. Only one study provided long-term clinical outcomes, including cardiovascular mortality and progression of cardiomyopathy, but for comparison reasons we used a shorter follow-up period as in the majority of studies for non-clinical outcomes.[27] The mean sample size was 330 (minimum 77, maximum 910).

Although the ongoing studies are not included in the evidence synthesis, we have described them in detail (see **Table 1**) in order to explain how they might be able to answer our main question in the near future. Patients in these studies are being recruited from Argentina, Bolivia, Brazil, Colombia, and Spain. Only one of them will use an adjusted dose[31] and all of them will use PCR to detect presence of parasites.

Excluded studies

We excluded 12 studies, three of these were duplicate records. The reasons for excluding the other nine[35-43] are described in **Appendix 3: Excluded studies and reasons for exclusion**; seven of these were excluded due to a wrong study design.

Risk of bias in included studies

Only one study was considered of unclear risk of bias for the randomization domains[17]; two studies were considered of high risk for blinding assessment[17, [17, 29] and one study for blinding of participants and personnel[29]; and two studies presented unclear risk for selective reporting[34, 44]((see **Figure 2 risk of bias item for each included study,** and in **Appendix 4. Support for judgement of included studies by risk of bias item** and **Risk of bias graph across all included studies)**.

Con formato: Izquierda, Interlineado: sencillo

Figure 2 Risk of bias item for each included study

	Random sequence generation	Allocation concealment	Blinding of participants and personnel	Blinding of outcome assessment O	Blinding of outcome assessment S	Incomplete outcome data	Selective reporting	Other bias
BENDITA 2017a	+	+	+	+	+	+	?	+
Molina 2014	+	+	−	+	−	+	+	+
Morillo 2017	+	+	−	+	+	+	+	+
Rodriges Coura 1997	+	+	?	+	?	+	?	+
Torrico 2018	+	+	+	?	+	+	+	+
TRAENA 2015a	+	+	+	+	+	−	?	?

O: Objective outcomes; **S**: Subjective outcomes

Effects of interventions

In considering the main question of this review, we focused the results on comparisons that included both fixed and adjusted doses of BZN, for which we presented GRADE summary of finding tables.

Benznidazole fixed vs adjusted dose

There was no head-to-head study exploring this comparison but, based on inferences from indirect comparisons of BZN-treated patients versus placebo, using PCR at one year and safety outcomes (see BZN versus placebo below), we did not observe important differences between fixed and adjusted doses (see **Table 2 Summary of findings: Benznidazole fixed vs adjusted dose**).The certainty of evidence for these outcomes was downgraded one or two levels because of indirectness.

Benznidazole at different fixed doses

We identified the unpublished study BENDITA[16], which did not find important differences in positive PCR between BZN 150 vs 300 for 8 weeks (RR 1.00; IC95% 0.24-4.18), 150 vs 300 for 4 weeks (RR 1.20; IC95% 0.27-5.25), and 150 vs 300 for 2 weeks (RR 0.86; IC95% 0.21, 3.47). The authors also found no differences in -AEs for the same comparisons: RR 0.88 (IC95% 0.56, 1.36), RR 1.05 (IC95% 0.65, 1.69), and RR 0.86 (IC 95% 0.49, 1.50) respectively. There were very few serious adverse events (SAEs) and drug discontinuations due to AEs, and no evidence of differences between groups. However, the study design was unpowered to detect differences between arms.

Benznidazole versus placebo

Efficacy was determined by positive serology, positive PCR, positive xenodiagnosis, mean reduction of antibody titer, mean reduction in PCR load, and clinical outcomes at the end of follow-up.

Positive serology: we identified one published study[28]~~[34]~~ without differences between groups (RR 1.00; IC95% 0.93-1.08) and one study[27] that favors treatment with BZN (RR 0.88; IC95% 0.84-0.93), both using an adjusted dose. The pooled RR was 0.94 (IC95% 0.82-1.08) and the certainty of evidence was considered low (see **Figure 3. Positive Serology at one year).**~~**Figure 3. Positive Serology at one year).**~~ The TRAENA study[27] showed a RR of 0.65 (IC95% 0.58-0.63) at six years.

Figure 3. Positive serology~~Positive serology~~ at one year

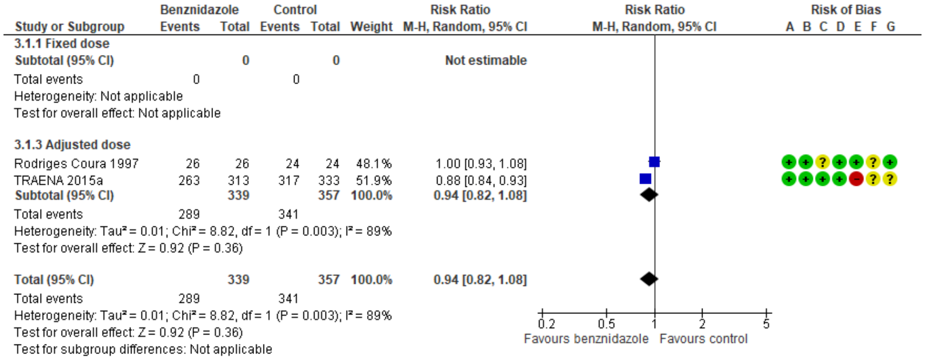

A) Random sequence generation (selection bias), (**B**) allocation concealment (selection bias), (**C**) blinding of participants and personnel (performance bias), (**D**) blinding of outcome assessment (detection bias), (**E**) incomplete outcome data (attrition bias), (**F**) selective reporting (reporting bias), (**G**) other bias

Positive PCR: both the fixed[17] and adjusted doses[16, 30] of BZN were effective at reducing positive PCR at one year against placebo (RR 0.20; IC95% 0.10-0.30) without differences in subgroups between the fixed dose of 300 mg/day for 14 to 56 days and the adjusted dose: RR 0.12 (IC95% 0.04-0.36) and RR 0.19 (IC95% 0.10-0.37), respectively. The significance of the test for

subgroup differences was Chi² = 0.56, df = 1 ($P = 0.45$), $I^2 = 0\%$ (see **Figure 4. Positive PCR**~~**Figure 4. Positive PCR**~~ and **Table 3. Summary of findings: Benznidazole versus placebo**).

Figure 4. Positive PCR at one year

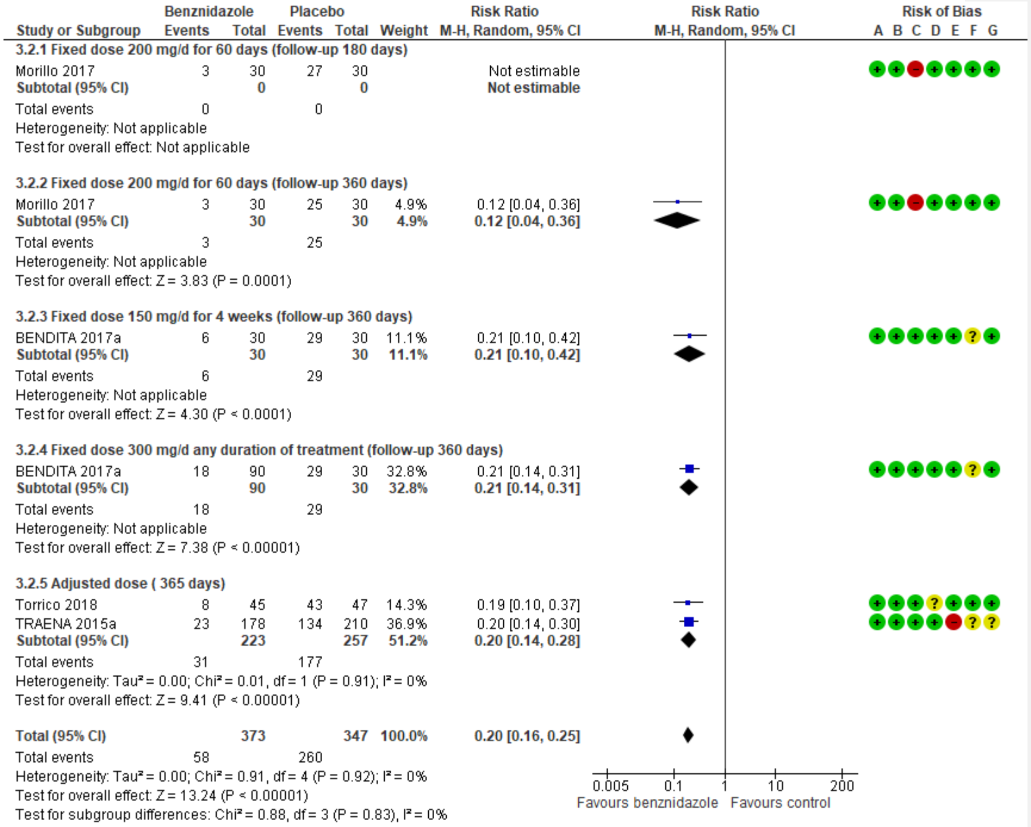

A) Random sequence generation (selection bias), **B**) allocation concealment (selection bias), **C**) blinding of participants and personnel (performance bias), **D)** blinding of outcome assessment objective outcomes (detection bias), **E**) incomplete outcome data (attrition bias), **F**) selective reporting (reporting bias), **H**) other bias

Positive xenodiagnosis: only one study provided data about an adjusted dose[28], favoring BZN (RR 0.12, 95% CI 0.04 to 0.36; participants = 60; studies = 1).~~Positive xenodiagnosis: only one study provided data about an adjusted dose[34], favoring BZN (RR 0.12, 95% CI 0.04 to 0.36; participants = 60; studies = 1).~~

Mean reduction in PCR load (GMT one year): Two studies provided data about an adjusted dose[27, 30] and showed no significant difference: (MD -0.48; 95% CI -1.19, 0.23, participants = 480; studies = 2).

Mean reduction of antibody titer at one year: only one study (92 participants) provided data about an adjusted dose[30] and showed no difference with placebo (Conventional ELISA: MD 0.01, 95% CI -0.17 to 0.19 and AT CL−ELISA: MD 0.01, 95% CI -0.07 to 0.09) except when using the geometric mean measured by AT CL−ELISA (MD -0.57, 95% CI -1.08 to -0.06).

Clinical outcomes at the end of follow-up: one long-term study (n= 713) that used an adjusted dose of BZN vs. placebo provided data about these outcomes,[27] and showed a RR of 0.89 (95% CI 0.62 to 1.26) for progression of cardiomyopathy, RR 1.18, (95% CI 0.40 to 3.49) for cardiovascular mortality and RR 0.38 (95% CI 0.10 to 1.42) for pacemaker implantation, implantable cardioverters or severe arrhythmia with hemodynamic unbalance, and cardiac failure.

As expected, the frequency of AEs is higher with BZN (data not shown). Only three outcomes presented studies that used fixed or adjusted dose against placebo: drug discontinuation, peripheral neuropathy (considered SAEs), and mild rash (considered non-serious AEs).

Drug discontinuation: both the fixed and adjusted doses showed more drug discontinuation than placebo and we found no subgroup difference: Test for subgroup differences: $Chi^2 = 0.12$, df = 1 (P = 0.73), $I^2 = 0\%$ 9 (see **Figure 5 Drug discontinuation** and **Table 3. Summary of findings: Benznidazole versus placebo**.

Figure 5. Drug discontinuation

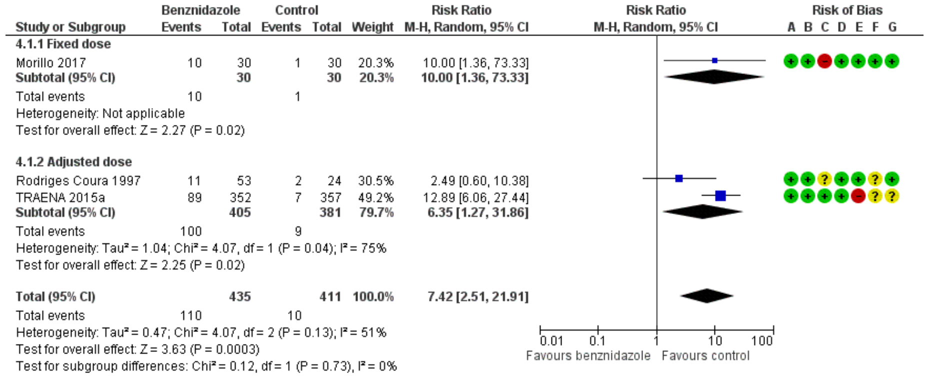

A) Random sequence generation (selection bias), **B**) allocation concealment (selection bias), **C**) blinding of participants and personnel (performance bias), **D)** blinding of outcome assessment objective outcomes (detection bias), **E**) incomplete outcome data (attrition bias), **F**) selective reporting (reporting bias), **H**) other bias

Peripheral neuropathy: we found no subgroup differences: Test for subgroup differences: Chi² = 1.50, df = 1 (P = 0.22), I² = 33.2%. See **Figure 6 Peripheral neuropathy and Table 3. Summary of findings: Benznidazole versus placebo**.

Figure 6. Peripheral neuropathy

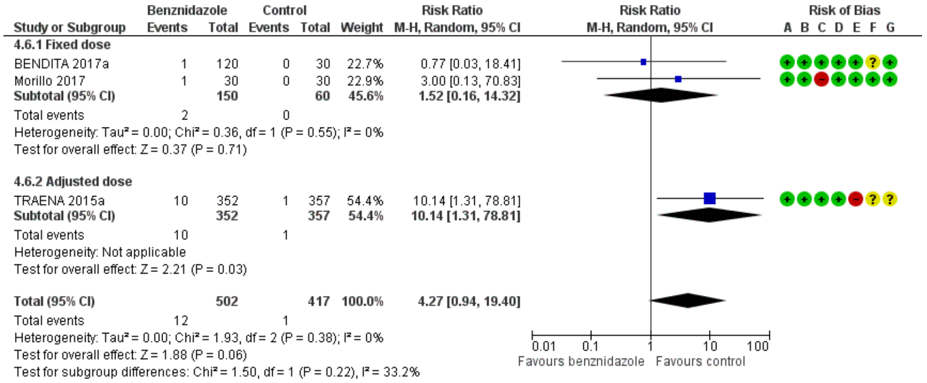

A) Random sequence generation (selection bias), **B**) allocation concealment (selection bias), **C**) blinding of participants and personnel (performance bias), **D)** blinding of outcome assessment objective outcomes (detection bias), **E**) incomplete outcome data (attrition bias), **F**) selective reporting (reporting bias), **H**) other bias

Mild rash: we found no subgroup difference: Test for subgroup differences: Chi² = 0.14, df = 1 (P = 0.93), I² = 0%) (see **Figure 7 Mild rash** and **Table 3. Summary of findings: Benznidazole versus placebo**).

Figure 7. Mild rash

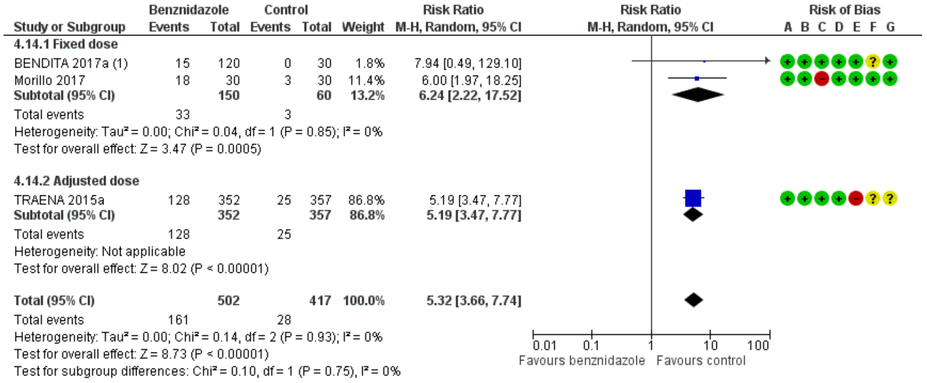

A) Random sequence generation (selection bias), **B**) allocation concealment (selection bias), **C**) blinding of participants and personnel (performance bias), **D)** blinding of outcome assessment objective outcomes (detection bias), **E**) incomplete outcome data (attrition bias), **F**) selective reporting (reporting bias), **H**) other bias

Benznidazole versus posaconazole

We identified two studies that used fixed-dose BZN versus posaconazole[17, [17, 29] and considering the longest follow-up period, both favored BZN (RR 0.24, 95% CI 0.06 to 0.93; participants = 112; studies = 2; I^2 = 81%). Test for subgroup differences by follow-up: Chi^2 = 4.17, df = 1 (P = 0.04), I^2 = 76.0% due to higher effect in Morillo 2017 (follow-up 360 days)[17] than in Molina 2014 (follow-up 280 days)[29].

Subgroup analysis and sensitivity analysis

Due to the low number of included studies it was not possible to conduct the planned subgroup analyses, except for the fixed and adjusted doses related to the question of our review.

For the same reason, it was not possible to conduct the planned sensitivity analyses, restricting the analysis to only studies with low risk of selection bias (associated with sequence generation or allocation concealment). Morillo et al.[17] (unclear risk of bias for this domain) was the only fixed-dose study included in the subgroup comparison between fixed and adjusted doses of BZN.

All studies included in the subgroup comparison between fixed and adjusted doses of BZN were of low risk of performance bias (associated with issues of blinding) and low risk of attrition bias (associated with completeness of data) except for TRAENA[27]. We found consistency of results applying both fixed-effect and random-effects models and also using OR and RD.

Discussion

The only drugs with proven efficacy against Chagas disease are BZN and nifurtimox. BZN is used in children and adults and is registered for use in adjusted-dose schemes. However, some investigators have proposed the more flexible use of fixed dosing regardless of body weight. After searching all completed and ongoing RCTs involving BZN at any dose, we found no direct comparison between fixed and adjusted doses of BZN.

We only found one efficacy outcome (positive PCR) and three safety outcomes (drug discontinuation, peripheral neuropathy, and mild rash) that allowed the subgroup comparisons between fixed and adjusted doses of BZN. The low or null I^2 for all these subgroup comparisons suggest no important clinical, methodological, or statistical differences in the observed effects by type of dosing. Since these are inferences from indirect comparisons of BZN-treated patients versus placebo, the certainty of evidence for these outcomes was consequently downgraded one or two levels because of indirectness.

We found a high certainty of evidence for the direct comparisons between BZN versus placebo for the efficacy outcome (positive PCR) and for the three safety outcomes (drug discontinuation, peripheral neuropathy and mild rash). However, these four outcomes are considered as moderate certainty of the evidence for the comparison fixed vs adjusted dose of benznidazole after downgrading one level due to indirectness (indirect comparisons). The certainty of the evidence was considered to be very low for cardiovascular mortality and progression of cardiomyopathy due to significant uncertainty between the surrogate outcome positive PCR and these critical outcomes. The certainty of the evidence was considered low for any SAE due to uncertainty between this outcome and the surrogate outcome drug discontinuation. See **Table 2 Summary of**

findings: Benznidazole fixed vs adjusted dose (based on indirect comparisons)See Summary of findings table 1 (based on indirect comparisons) and Table 3. **Summary of findings**: Benznidazole versus placebo table 2, (for direct comparisons between BZN and placebo by type of dose).

Con formato: Fuente: Negrita, Revisar la ortografía y la gramática

We found six related systematic reviews that showed similar results in terms of the effect of interventions against placebo, however, none of them addressed our question concerning the comparison of fixed and adjusted doses of BZN [39, 43, 45-48]. Observational studies suggest that treatment could be better that no treatment even in the early phases of CCC[46].[45]. Unfortunately, non-RCT studies used adjusted doses of BZN, not allowing the assessment of subgroup analysis by BZN scheme. Unlike other reviews, we only included RCTs to reduce the risk of bias, but, as was the case for the previous studies, we had to deal with differences in the populations studied, follow-up periods, diagnostic techniques, and sample size.

Demonstration that a fixed dose of BZN has a similar profile in relation to safety and efficacy would allow a review of the current guidelines and the recommendation of fixed doses, eliminating one barrier to treatment management, and improving compliance by patients and health workers.

The strengths of this systematic review of RCTs include the registration of its protocol, the complete literature search, the rigorous Cochrane methods used, the participation of most principal investigators of the RCTs included, and the inclusion of valuable unpublished data. All these factors make our study the most complete evidence synthesis currently available that addresses the comparative efficacy and safety of adjusted-dose BZN for *T. cruzi* seropositive adults mainly without CCC.

The population included in the trials is representative of the population of adults with chronic Chagas disease without cardiomyopathy, however important limitations need to be mentioned.

First of all, the absence of direct comparisons between fixed and adjusted doses of BNZ, and the assessments of critical outcomes should be noticed. Additionally, the paucity of studies prevented us from performing our planned subgroup and sensitivity analyses. The unpublished TRAENA study[27] was the only one that succeeded in assessing long-term clinical outcomes.

Individual patient data (IPD) meta-analysis could address these issues, for example by including cumulative dose assessments. Moreover, network IPD meta-analysis could formally enhance indirect comparisons.

Conclusion

Based on a low to very low certainty of evidence for critical clinical outcomes and a moderate certainty of evidence for important outcomes, fixed and adjusted doses of BZN might be considered equivalent in terms of efficacy and safety.

An IPD meta-analysis would allow us to conduct the planned subgroup analysis and meta-regressions, but given the absence of a direct comparison between fixed and adjusted doses of BZN, the only approach to gain in certainty of evidence to address the objective of the review would be an IPD network-meta-analysis - an approach which our research group is currently following.

Financial support

Independent grant from Drugs for Neglected Diseases *initiative* (DND*i*).

Acknowledgments

We want to acknowledge Daniel Comandé for his contribution to the search strategy.

Table 1. Main characteristics of included and ongoing studies

Short title	PI	Start/end years	N	Countries	Study status	Benznidazole dose	Comparison	Outcomes
Rodrigues C. 1997[28]Rodrigues C. 1997[34]	Rodrigues Coura, J.	<1997	77	Brazil	Completed	Adjusted	Nifurtimox/ Placebo	Serology / Xenodiagnoses
E1224[30]	Torrico, F.	2011/2	231	Bolivia	Completed	Adjusted	E1224 / Placebo	PCR
CHAGASAZOL [29]	Molina, C.	2010/1	79	Spain	Completed	Fixed	Posaconazole	PCR
STOP-CHAGAS[17]	Morillo, I.	2011/3	120	Argentina, Chile, México, Guatemala, Spain	Completed	Fixed	Posaconazole / Placebo	PCR
BENDITA[16]	Torrico , F.	2017/8	210	Bolivia	Completed Unpublished	Fixed	E1224 / Placebo	PCR
TRAENA[27]	Riarte, A.	1999/ 2015	910	Argentina	Completed Unpublished	Adjusted	Placebo	PCR Serology Cardiovascular Mortality, Progression
BETTY[32]	Buekens, P.	2019/	600	Argentina	Ongoing	Fixed	Benznidazole 300 mg	PCR
CHICAMOCHA 3[31, 47, 48], 49, 50]	Villar, JC.	2015/	500	Colombia	Ongoing	Adjusted	Nifurtimox/ Placebo	PCR
MULTIBENZ [18]	Molina, I.	2017/	240	Spain, Brazil Argentina, Colombia	Ongoing	Fixed	Benznidazole 150/400 mg	PCR
TESEO[33]	Almeida, IC.	2019	450	Bolivia	Ongoing	Fixed	Benznidazole 150/300 mg Nifurtimox 240/480 mg	RT-PCR

Table 2. Summary of findings: Benznidazole fixed vs adjusted dose

Outcome	Impact	№ of participants (studies)	Certainty
**Efficacy***			
**Positive PCR**	No important difference between fixed and adjusted dose (subgroup differences $I^2 = 0\%$)	152 (2 RCTs)	⊕⊕⊕◯[1] MODERATE
**Cardiovascular mortality**	Based only in the surrogate outcome + PCR, differences between groups on critical outcomes are uncertain	152 (2 RCTs)	⊕◯◯◯[2] VERY LOW
**Progression of cardiomyopathy**		152 (2 RCTs)	⊕◯◯◯[2] VERY LOW
**Safety#**			
**Drug discontinuation**	No important difference between fixed and adjusted dose (Subgroup differences: $I^2 = 0\%$)	846 (3 RCTs)	⊕⊕⊕◯[1] MODERATE
**Peripheral neuropathy**		769 (2 studies)	⊕⊕⊕◯[1] MODERATE
**Mild rash**		769 (2 studies)	⊕⊕⊕◯[1] MODERATE
**Any serious adverse events**	Based only in the surrogate outcome drug discontinuation, differences between groups on this critical outcome is uncertain	846 (3 RCTs)	⊕⊕◯◯[3] LOW

Refer to text for benznidazole versus placebo: Efficacy (PCR at one year*) and Safety#

[1]Downgraded one level due to serious indirectness, since these are inferences from subgroup analysis of comparisons between BZN and placebo.

[2]Downgraded two levels due to due to very serious indirectness (important uncertainty between the surrogate outcome + PCR and the critical outcomes cardiovascular mortality and progression of cardiomyopathy) and methodological limitations to answering this question.

[3]Downgraded one level due to serious indirectness, since there is uncertainty between surrogate outcome drug discontinuation and SAEs.

Table 3. Summary of findings: Benznidazole versus placebo

Outcome	Absolute effects (95% CI)		Relative effect (95% CI)	№ of participants (studies)	Certainty
	Risk with placebo: efficacy	Risk with benznidazole			
**Positive PCR**	883 per 1.000	**150 per 1.000 (88 to 265)**	**RR 0.20 (0.16 to 0.25)**	152 (2 RCTs)	⨁⨁⨁⨁ HIGH
- Fixed dose 300 mg/day for 14 to 56 days	833 per 1.000	**100 per 1.000 (33 to 300)**	**RR 0.12 (0.04 to 0.36)**	60 (1 RCT)	⨁⨁⨁⨁ HIGH
- Adjusted dose	915 per 1.000	**174 per 1.000 (91 to 339)**	**RR 0.19 (0.10 to 0.37)**	92 (1 RCT)	⨁⨁⨁⨁ HIGH
**Drug discontinuation**	24 per 1.000	**181 per 1.000 (61 to 533)**	**RR 7.42 (2.51 to 21.91)**	846 (3 RCTs)	⨁⨁⨁⨁ HIGH
- Fixed dose	33 per 1.000	**333 per 1.000 (45 to 1.000)**	**RR 10.00 (1.36 to 73.33)**	60 (1 RCT)	⨁⨁⨁⨁ HIGH
- Adjusted dose	24 per 1.000	**150 per 1.000 (30 to 753)**	**RR 6.35 (1.27 to 31.86)**	786 (2 RCTs)	⨁⨁⨁⨁ HIGH
**Peripheral neuropathy**	2 per 1000	**10 per 1000 (2 to 47)**	**RR 4.27 (0.94 to 19.40)**	919 (3 RCTs)	⨁⨁⨁⨁ HIGH
- Fixed dose	0 per 1000	**0 per 1000 (0 to 0)**	**RR 1.52 (0.16 to 14.32)**	210 (2 RCTs)	⨁⨁⨁⨁ HIGH
- Adjusted dose	3 per 1000	**28 per 1000 (4 to 221)**	**RR 10.14 (1.31 to 78.81)**	709 (1 RCT)	⨁⨁⨁⨁ HIGH
**Mild rash**	67 per 1000	**357 per 1000 (246 to 520)**	**RR 5.32 (3.66 to 7.74)**	919 (3 RCTs)	⨁⨁⨁⨁ HIGH
- Fixed dose	50 per 1000	**312 per 1000 (111 to 876)**	**RR 6.24 (2.22 to 17.52)**	210 (2 RCTs)	⨁⨁⨁⨁ HIGH
- Adjusted dose	70 per 1000	**363 per 1000 (243 to 544)**	**RR 5.19 (3.47 to 7.77)**	709 (1 RCT)	⨁⨁⨁⨁ HIGH

References

1. Stanaway JD, Roth G. The burden of Chagas disease: estimates and challenges. Glob Heart. 2015;10(3):139-44. Epub 2015/09/27. doi: 10.1016/j.gheart.2015.06.001. PubMed PMID: 26407508.
2. Global Burden of Disease (GBD) for Chagas disease 2017 [December 28th 2019]. Available from: https://vizhub.healthdata.org/gbd-compare/.
3. Lee BY, Bacon Km Fau - Bottazzi ME, Bottazzi Me Fau - Hotez PJ, Hotez PJ. Global economic burden of Chagas disease: a computational simulation model. (1474-4457 (Electronic)).
4. Hotez PJ, Bottazzi ME, Franco-Paredes C, Ault SK, Periago MR. The neglected tropical diseases of Latin America and the Caribbean: a review of disease burden and distribution and a roadmap for control and elimination. PLoS Neglected Tropical Diseases 2008;2(9):e300.
5. Bartsch SM, Bottazzi ME, Asti L, Strych U, Meymandi S, Falcon-Lezama JA, et al. Economic value of a therapeutic Chagas vaccine for indeterminate and Chagasic cardiomyopathy patients. Vaccine. 2019;37(28):3704-14. Epub 2019/05/21. doi: 10.1016/j.vaccine.2019.05.028. PubMed PMID: 31104883.
6. Coura JR, Vinas PA. Chagas disease: a new worldwide challenge. Nature 2010;465:S6-7.
7. Bern C, Montgomery SP. An estimate of the burden of Chagas disease in the United States. Clinical Infectious Diseases 2009;49(5):e52-4.
8. WHO. Control and prevention of Chagas disease in Europe, 2010. www.fac.org.ar/1/comites/chagas/Chagas_WHO_Technical%20Report_16_06_10.pdf (accessed 2 Nov2018).
9. Bocchi EA. Heart failure in South America. Curr Cardiol Rev. 2013;9(2):147-56. Epub 2013/04/20. doi: 10.2174/1573403x11309020007. PubMed PMID: 23597301; PubMed Central PMCID: PMCPMC3682398.
10. Moncayo A. Chagas disease: current epidemiological trends after the interruption of vectorial and transfusional transmission in the Southern Cone countries. Memorios do Instituto Oswaldo Cruz 2003;98(5):577-91.
11. Sosa-Estani S, Segura EL. Integrated control of Chagas disease for its elimination as public health problem--a review. Mem Inst Oswaldo Cruz. 2015;110(3):289-98. Epub 2015/05/21. doi: 10.1590/0074-02760140408. PubMed PMID: 25993503; PubMed Central PMCID: PMCPMC4489466.
12. Viotti R, Alarcon de Noya B, Araujo-Jorge T, Grijalva MJ, Guhl F, Lopez MC, et al. Towards a paradigm shift in the treatment of chronic Chagas disease. Antimicrob Agents Chemother. 2014;58(2):635-9. Epub 2013/11/20. doi: 10.1128/AAC.01662-13. PubMed PMID: 24247135; PubMed Central PMCID: PMCPMC3910900.
13. Villar JC. Commentary: control of Chagas' disease: let's put people before vectors. International Journal of Epidemiology 2001;30(4):894-5.
14. Pautas para la atención al paciente infectado con Trypanosoma cruzi (Enfermedad de Chagas). Buenos Aires: Ministerio de Salud, Argentina; 2015.
15. Pinazo MJ, Guerrero L Fau - Posada E, Posada E Fau - Rodriguez E, Rodriguez E Fau - Soy D, Soy D Fau - Gascon J, Gascon J. Benznidazole-related adverse drug reactions and their relationship to serum drug concentrations in patients with chronic chagas disease. (1098-6596 (Electronic)).
16. (03378661) B. BENDITA BEnznidazole New Doses Improved Treatment and Associations. 2017.
17. Morillo CA, Waskin H, Sosa-Estani S, Del Carmen Bangher M, Cuneo C, Milesi R, et al. Benznidazole and Posaconazole in Eliminating Parasites in Asymptomatic T. Cruzi Carriers: The STOP-CHAGAS Trial. J Am Coll Cardiol. 2017;69(8):939-47. doi: 10.1016/j.jacc.2016.12.023. PubMed PMID: 28231946.
18. (03191162) M. Evaluation of Different Benznidazole Regimens for the Treatment of Chronic Chagas Disease. https://clinicaltrialsgov/ct2/show/NCT03191162. 2018.
19. Higgins J, Thomas Je. Cochrane Handbook for Systematic Reviews of Interventions Version 6 [updated August 2019]. 2019. Available from: Available from https://training.cochrane.org/handbook/current.
20. Liberati A, Altman DG, Tetzlaff J, Mulrow C, Gotzsche PC, Ioannidis JP, et al. The PRISMA statement for reporting systematic reviews and meta-analyses of studies that evaluate health care interventions: explanation and elaboration. PLoS Med. 2009;6(7):e1000100. Epub 2009/07/22. doi: 10.1371/journal.pmed.1000100. PubMed PMID: 19621070.
21. Moher D, Liberati A, Tetzlaff J, Altman DG. Preferred reporting items for systematic reviews and meta-analyses: the PRISMA statement. PLoS Med. 2009;6(7):e1000097. Epub 2009/07/22. doi: 10.1371/journal.pmed.1000097. PubMed PMID: 19621072.
22. Higgins J, Savović J, Page M, Elbers R, Sterne J. Chapter 8: Assessing risk of bias in a randomized trial. 2018. In: Cochrane Handbook for Systematic Reviews of Interventions Version 6 [updated August 2019] [Internet]. Cochrane. Available from: Available from www.cochrane-handbook.org.
23. Review Manager (RevMan). [Computer program] Version 53 Copenhagen: The Nordic Cochrane Centre, The Cochrane Collaboration; 2014.
24. Guyatt G, Oxman AD, Akl EA, Kunz R, Vist G, Brozek J, et al. GRADE guidelines: 1. Introduction-GRADE evidence profiles and summary of findings tables. J Clin Epidemiol. 2011;64(4):383-94. Epub 2011/01/05. doi: S0895-4356(10)00330-6 [pii] 10.1016/j.jclinepi.2010.04.026. PubMed PMID: 21195583.
25. Schünemann H, Vist G, Higgins J, Santesso N, Deeks J, Glasziou P, et al. Chapter 15: Interpreting results and drawing conclusions. 2018. In: Cochrane Handbook for Systematic Reviews of Interventions Version 6 [updated September 2018] [Internet]. Cochrane. Available from: Available from www.cochrane-handbook.org.
26. Schünemann H, Brożek J, Guyatt G, Oxman A, (editors). Handbook for grading the quality of evidence and the strength of recommendations using the GRADE approach [updated October 2013]. 2013. Available from: Available from http://gdt.guidelinedevelopment.org/app/index.html.
27. (02386358) T. Etiologic Treatment With Benznidazole in Adult Patients With Chronic Chagas Disease. A Randomized Clinical Trial. 2015.
28. Coura JR, de Abreu LL, Willcox HP, Petana W. [Comparative controlled study on the use of benznidazole, nifurtimox and placebo, in the chronic form of Chagas' disease, in a field area with interrupted transmission. I. Preliminary evaluation]. Revista da Sociedade Brasileira de Medicina Tropical. 1997;30(2):139-44.
29. Molina I, Gomez i Prat J, Salvador F, Trevino B, Sulleiro E, Serre N, et al. Randomized trial of posaconazole and benznidazole for chronic Chagas' disease. N Engl J Med. 2014;370(20):1899-908. doi: 10.1056/NEJMoa1313122. PubMed PMID: 24827034.

30. Torrico F, Gascon J, Ortiz L, Alonso-Vega C, Pinazo MJ, Schijman A, et al. Treatment of adult chronic indeterminate Chagas disease with benznidazole and three E1224 dosing regimens: a proof-of-concept, randomised, placebo-controlled trial. Lancet Infect Dis. 2018;18(4):419-30. doi: 10.1016/S1473-3099(17)30538-8. PubMed PMID: 29352704.
31. (02369978) C. CHICAMOCHA 3 - Equivalence of Usual Interventions for Trypanosomiasis (EQUITY). https://clinicaltrialsgov/ct2/show/NCT02369978. 2015.
32. (03672487) B. Short-course Benznidazole Treatment to Reduce Trypanosoma Cruzi Parasitic Load in Women of Reproductive Age. https://clinicaltrialsgov/ct2/show/NCT03672487. 2018.
33. (03981523) T. New Therapies and Biomarkers for Chagas Infection. https://clinicaltrialsgov/show/NCT03981523. 2019. PubMed PMID: CN-01945435.
34. Álvarez MG, Hernández Y, Bertocchi G, Fernández M, Lococo B, Ramírez JC, et al. New scheme of intermittent benznidazole administration in patients chronically infected with Trypanosoma cruzi: A pilot short-term follow-up study with adult patients. Antimicrobial Agents and Chemotherapy. 2016;60(2):833-7. doi: 10.1128/AAC.00745-15.
35. Andrade AL, Martelli CM, Oliveira RM, Silva SA, Aires AI, Soussumi LM, et al. Short report: benznidazole efficacy among Trypanosoma cruzi-infected adolescents after a six-year follow-up. American journal of tropical medicine and hygiene. 2004;71(5):594-7. PubMed PMID: CN-00560425.
36. Apt W, Arribada A, Arab F, Ugarte JM, Luksic I, Solé C. Clinical trial of benznidazole and an immunopotentiator against Chagas disease in Chile. Transactions of the Royal Society of Tropical Medicine and Hygiene. 1986;80(6):1010.
37. Molina I, Prat JGI, Salvador F, Treviño B, Serre N, Sulleiro E, et al. Phase IIb clinical trial with posaconazol and benznidazol for chronic Chagas disease. Tropical Medicine and International Health. 2013;18:60. doi: 10.1111/tmi.12162.
38. Müller Kratz J, Garcia Bournissen F, Forsyth CJ, Sosa-Estani S. Clinical and pharmacological profile of benznidazole for treatment of Chagas disease. Expert Review of Clinical Pharmacology. 2018;11(10):943-57. doi: 10.1080/17512433.2018.1509704.
39. Pérez-Molina JA, Pérez-Ayala A, Moreno S, Fernández-González MCMC, Zamora J, López-Velez R. Use of benznidazole to treat chronic Chagas' disease: A systematic review with a meta-analysis. Journal of Antimicrobial Chemotherapy. 2009;64(6):1139-47. doi: 10.1093/jac/dkp357.
40. Sguassero Y, Cuesta CB, Roberts KN, Hicks E, Comandé D, Ciapponi A, et al. Course of chronic Trypanosoma cruzi infection after treatment based on parasitological and serological tests: A systematic review of follow-up studies. PLoS ONE. 2015;10(10):e0139363. doi: 10.1371/journal.pone.0139363.
41. Sosa-Estani S, Armenti A, Araujo G, Viotti R, Lococo B, Ruiz Vera B, et al. Treatment of Chagas disease with benznidazole and thioctic acid. Medicina. 2004;64(1):1-6. PubMed PMID: CN-00480924.
42. Villar JC, Perez JG, Cortes OL, Riarte A, Pepper M, Marin-Neto JA, et al. Trypanocidal drugs for chronic asymptomatic Trypanosoma cruzi infection. Cochrane Database of Systematic Reviews. 2014;(5). doi: 10.1002/14651858.CD003463.pub2. PubMed PMID: CD003463.
43. Prado N, Hernández Y, De Rissio AM, Esteve M, Riarte A. La seguridad en un ensayo clínico aleatorizado (ECA) - Estudio TRAENA - en pacientes adultos con Enfermedad de Chagas. 2008 2008. Report No.
44. Bern C, Montgomery SP, Herwaldt BL, Rassi A, Marin-Neto JA, Dantas RO, et al. Evaluation and treatment of chagas disease in the United States: a systematic review. JAMA. 2007;298(18):2171-81. doi: 10.1001/jama.298.18.2171.
45. Echeverria LE, Morillo CA. American Trypanosomiasis (Chagas Disease). Infect Dis Clin North Am. 2019;33(1):119-34. Epub 2019/02/05. doi: 10.1016/j.idc.2018.10.015. PubMed PMID: 30712757.
46. Reyes PPA, Vallejo M. Trypanocidal drugs for late stage, symptomatic Chagas disease (Trypanosoma cruzi infection). Cochrane Database of Systematic Reviews. 2005;(4):CD004102. doi: 10.1002/14651858.CD004102.pub2.
47. Villar JC, Herrera VM, Perez Carreno JG, Vaquiro Herrera E, Castellanos Dominguez YZ, Vasquez SM, et al. Nifurtimox versus benznidazole or placebo for asymptomatic Trypanosoma cruzi infection (Equivalence of Usual Interventions for Trypanosomiasis - EQUITY): study protocol for a randomised controlled trial. Trials. 2019;20(1):431. Epub 2019/07/17. doi: 10.1186/s13063-019-3423-3. PubMed PMID: 31307503; PubMed Central PMCID: PMCPMC6631895.
48. Villar JC, Herrera VM, Carreno JGP, Herrera EV, Dominguez YZC, Vasquez SM, et al. Correction to: Nifurtimox versus benznidazole or placebo for asymptomatic Trypanosoma cruzi infection (Equivalence of Usual Interventions for Trypanosomiasis - EQUITY): study protocol for a randomised controlled trial. Trials. 2019;20(1):516. Epub 2019/08/21. doi: 10.1186/s13063-019-3630-y. PubMed PMID: 31429793; PubMed Central PMCID: PMCPMC6701118.

1. ~~Stanaway JD, Roth G. The burden of Chagas disease: estimates and challenges. Glob Heart. 2015;10(3):139-44. Epub 2015/09/27. doi: 10.1016/j.gheart.2015.06.001. PubMed PMID: 26407508.~~
2. ~~Global Burden of Disease (GBD) for Chagas disease 2017 [December 28th 2019]. Available from: https://vizhub.healthdata.org/gbd-compare/.~~
3. ~~Lee BY, Bacon Km Fau - Bottazzi ME, Bottazzi Me Fau - Hotez PJ, Hotez PJ. Global economic burden of Chagas disease: a computational simulation model. (1474-4457 (Electronic)).~~
4. ~~Hotez PJ, Bottazzi ME, Franco-Paredes C, Ault SK, Periago MR. The neglected tropical diseases of Latin America and the Caribbean: a review of disease burden and distribution and a roadmap for control and elimination. PLoS Neglected Tropical Diseases 2008;2(9):e300.~~
5. ~~Bartsch SM, Bottazzi ME, Asti L, Strych U, Meymandi S, Falcon-Lezama JA, et al. Economic value of a therapeutic Chagas vaccine for indeterminate and Chagasic cardiomyopathy patients. Vaccine. 2019;37(28):3704-14. Epub 2019/05/21. doi: 10.1016/j.vaccine.2019.05.028. PubMed PMID: 31104883.~~
6. ~~Coura JR, Vinas PA. Chagas disease: a new worldwide challenge. Nature 2010;465:S6-7.~~
7. ~~Bern C, Montgomery SP. An estimate of the burden of Chagas disease in the United States. Clinical Infectious Diseases 2009;49(5):e52-4.~~
8. ~~WHO. Control and prevention of Chagas disease in Europe, 2010. www.fac.org.ar/1/comites/chagas/Chagas_WHO_Technical%20Report_16_06_10.pdf (accessed 2 Nov2018).~~

9. Bocchi EA. Heart failure in South America. Curr Cardiol Rev. 2013;9(2):147-56. Epub 2013/04/20. doi: 10.2174/1573403x11309020007. PubMed PMID: 23597301; PubMed Central PMCID: PMCPMC3682398.
10. Moncayo A. Chagas disease: current epidemiological trends after the interruption of vectorial and transfusional transmission in the Southern Cone countries. Memorios do Instituto Oswaldo Cruz 2003;98(5):577-91.
11. Sosa-Estani S, Segura EL. Integrated control of Chagas disease for its elimination as public health problem--a review. Mem Inst Oswaldo Cruz. 2015;110(3):289-98. Epub 2015/05/21. doi: 10.1590/0074-02760140408. PubMed PMID: 25993503; PubMed Central PMCID: PMCPMC4489466.
12. Viotti R, Alarcon de Noya B, Araujo-Jorge T, Grijalva MJ, Guhl F, Lopez MC, et al. Towards a paradigm shift in the treatment of chronic Chagas disease. Antimicrob Agents Chemother. 2014;58(2):635-9. Epub 2013/11/20. doi: 10.1128/AAC.01662-13. PubMed PMID: 24247135; PubMed Central PMCID: PMCPMC3910900.
13. Villar JC. Commentary: control of Chagas' disease: let's put people before vectors. International Journal of Epidemiology 2001;30(4):894-5.
14. Pautas para la atención al paciente infectado con Trypanosoma cruzi (Enfermedad de Chagas). Buenos Aires: Ministerio de Salud, Argentina; 2015.
15. Pinazo MJ, Guerrero L Fau - Posada E, Posada E Fau - Rodriguez E, Rodriguez E Fau - Soy D, Soy D Fau - Gascon J, Gascon J. Benznidazole-related adverse drug reactions and their relationship to serum drug concentrations in patients with chronic chagas disease. (1098-6596 (Electronic)).
16. (03378661) B. BENDITA BEnznidazole New Doses Improved Treatment and Associations. 2017.
17. Morillo CA, Waskin H, Sosa-Estani S, Del Carmen Bangher M, Cuneo C, Milesi R, et al. Benznidazole and Posaconazole in Eliminating Parasites in Asymptomatic T. Cruzi Carriers: The STOP-CHAGAS Trial. J Am Coll Cardiol. 2017;69(8):939-47. doi: 10.1016/j.jacc.2016.12.023. PubMed PMID: 28231946.
18. (03191162) M. Evaluation of Different Benznidazole Regimens for the Treatment of Chronic Chagas Disease. https://clinicaltrialsgov/ct2/show/NCT03191162. 2018.
19. Higgins J, Thomas Je. Cochrane Handbook for Systematic Reviews of Interventions Version 6 [updated August 2019]. 2019. Available from: Available from https://training.cochrane.org/handbook/current.
20. Liberati A, Altman DG, Tetzlaff J, Mulrow C, Gotzsche PC, Ioannidis JP, et al. The PRISMA statement for reporting systematic reviews and meta-analyses of studies that evaluate health care interventions: explanation and elaboration. PLoS Med. 2009;6(7):e1000100. Epub 2009/07/22. doi: 10.1371/journal.pmed.1000100. PubMed PMID: 19621070.
21. Moher D, Liberati A, Tetzlaff J, Altman DG. Preferred reporting items for systematic reviews and meta-analyses: the PRISMA statement. PLoS Med. 2009;6(7):e1000097. Epub 2009/07/22. doi: 10.1371/journal.pmed.1000097. PubMed PMID: 19621072.
22. Higgins J, Savović J, Page M, Elbers R, Sterne J. Chapter 8: Assessing risk of bias in a randomized trial. 2018. In: Cochrane Handbook for Systematic Reviews of Interventions Version 6 [updated August 2019]
[Internet]. Cochrane. Available from: Available from www.cochrane-handbook.org.
23. Review Manager (RevMan). [Computer program] Version 53 Copenhagen: The Nordic Cochrane Centre, The Cochrane Collaboration; 2014.
24. Guyatt G, Oxman AD, Akl EA, Kunz R, Vist G, Brozek J, et al. GRADE guidelines: 1. Introduction-GRADE evidence profiles and summary of findings tables. J Clin Epidemiol. 2011;64(4):383-94. Epub 2011/01/05. doi: S0895-4356(10)00330-6 [pii]
10.1016/j.jclinepi.2010.04.026. PubMed PMID: 21195583.
25. Schünemann H, Vist G, Higgins J, Santesso N, Deeks J, Glasziou P, et al. Chapter 15: Interpreting results and drawing conclusions. 2018. In: Cochrane Handbook for Systematic Reviews of Interventions Version 6 [updated September 2018]
[Internet]. Cochrane. Available from: Available from www.cochrane-handbook.org.
26. Schünemann H, Brożek J, Guyatt G, Oxman A, (editors). Handbook for grading the quality of evidence and the strength of recommendations using the GRADE approach [updated October 2013]. 2013. Available from: Available from http://gdt.guidelinedevelopment.org/app/index.html.
27. (02386358) T. Etiologic Treatment With Benznidazole in Adult Patients With Chronic Chagas Disease. A Randomized Clinical Trial. 2015.
28. Coura JR, de Abreu LL, Willcox HP, Petana W. [Comparative controlled study on the use of benznidazole, nifurtimox and placebo, in the chronic form of Chagas' disease, in a field area with interrupted transmission. I. Preliminary evaluation]. Revista da Sociedade Brasileira de Medicina Tropical. 1997;30(2):139-44.
29. Molina I, Gomez i Prat J, Salvador F, Trevino B, Sulleiro E, Serre N, et al. Randomized trial of posaconazole and benznidazole for chronic Chagas' disease. N Engl J Med. 2014;370(20):1899-908. doi: 10.1056/NEJMoa1313122. PubMed PMID: 24827034.

30. Torrico F, Gascon J, Ortiz L, Alonso-Vega C, Pinazo MJ, Schijman A, et al. Treatment of adult chronic indeterminate Chagas disease with benznidazole and three E1224 dosing regimens: a proof-of-concept, randomised, placebo-controlled trial. Lancet Infect Dis. 2018;18(4):419-30. doi: 10.1016/S1473-3099(17)30538-8. PubMed PMID: 29352704.
31. (02369978) C. CHICAMOCHA 3 - Equivalence of Usual Interventions for Trypanosomiasis (EQUITY). https://clinicaltrialsgov/ct2/show/NCT02369978. 2015.
32. (03672487) B. Short-course Benznidazole Treatment to Reduce Trypanosoma Cruzi Parasitic Load in Women of Reproductive Age. https://clinicaltrialsgov/ct2/show/NCT03672487. 2018.
33. (03981523) T. New Therapies and Biomarkers for Chagas Infection. https://clinicaltrialsgov/show/NCT03981523. 2019. PubMed PMID: CN-01945435.
34. Rodrigues Coura J, de Abreu LL, Willcox HP, Petana W. [Comparative controlled study on the use of benznidazole, nifurtimox and placebo, in the chronic form of Chagas' disease, in a field area with interrupted transmission. I. Preliminary evaluation]. Rev Soc Bras Med Trop. 1997;30(2):139-44. PubMed PMID: 9148337.
35. Álvarez MG, Hernández Y, Bertocchi G, Fernández M, Lococo B, Ramírez JC, et al. New scheme of intermittent benznidazole administration in patients chronically infected with Trypanosoma cruzi: A pilot short-term follow-up study with adult patients. Antimicrobial Agents and Chemotherapy. 2016;60(2):833-7. doi: 10.1128/AAC.00745-15.
36. Andrade AL, Martelli CM, Oliveira RM, Silva SA, Aires AI, Soussumi LM, et al. Short report: benznidazole efficacy among Trypanosoma cruzi-infected adolescents after a six-year follow-up. American journal of tropical medicine and hygiene. 2004;71(5):594-7. PubMed PMID: CN-00560425.
37. Apt W, Arribada A, Arab F, Ugarte JM, Luksic I, Solé C. Clinical trial of benznidazole and an immunopotentiator against Chagas disease in Chile. Transactions of the Royal Society of Tropical Medicine and Hygiene. 1986;80(6):1010.
38. Molina I, Prat JGI, Salvador F, Treviño B, Serre N, Sulleiro E, et al. Phase IIb clinical trial with posaconazol and benznidazol for chronic Chagas disease. Tropical Medicine and International Health. 2013;18:60. doi: 10.1111/tmi.12162.
39. Müller Kratz J, Garcia Bournissen F, Forsyth CJ, Sosa-Estani S. Clinical and pharmacological profile of benznidazole for treatment of Chagas disease. Expert Review of Clinical Pharmacology. 2018;11(10):943-57. doi: 10.1080/17512433.2018.1509704.
40. Pérez-Molina JA, Pérez-Ayala A, Moreno S, Fernández-González MCMC, Zamora J, López-Velez R. Use of benznidazole to treat chronic Chagas' disease: A systematic review with a meta-analysis. Journal of Antimicrobial Chemotherapy. 2009;64(6):1139-47. doi: 10.1093/jac/dkp357.
41. Sguassero Y, Cuesta CB, Roberts KN, Hicks E, Comandé D, Ciapponi A, et al. Course of chronic Trypanosoma cruzi infection after treatment based on parasitological and serological tests: A systematic review of follow-up studies. PLoS ONE. 2015;10(10):e0139363. doi: 10.1371/journal.pone.0139363.
42. Sosa-Estani S, Armenti A, Araujo G, Viotti R, Lococo B, Ruiz Vera B, et al. Treatment of Chagas disease with benznidazole and thioctic acid. Medicina. 2004;64(1):1-6. PubMed PMID: CN-00480924.
43. Villar JC, Perez JG, Cortes OL, Riarte A, Pepper M, Marin-Neto JA, et al. Trypanocidal drugs for chronic asymptomatic Trypanosoma cruzi infection. Cochrane Database of Systematic Reviews. 2014;(5). doi: 10.1002/14651858.CD003463.pub2. PubMed PMID: CD003463.
44. Prado N, Hernández Y, De Rissio AM, Esteve M, Riarte A. La seguridad en un ensayo clínico aleatorizado (ECA) - Estudio TRAENA - en pacientes adultos con Enfermedad de Chagas. 2008 2008. Report No.
45. Reyes PPA, Vallejo M. Trypanocidal drugs for late stage, symptomatic Chagas disease (Trypanosoma cruzi infection). Cochrane Database of Systematic Reviews. 2005;(4):CD004102. doi: 10.1002/14651858.CD004102.pub2.
46. Bern C, Montgomery SP, Herwaldt BL, Rassi A, Marin-Neto JA, Dantas RO, et al. Evaluation and treatment of chagas disease in the United States: a systematic review. JAMA. 2007;298(18):2171-81. doi: 10.1001/jama.298.18.2171.
47. Pérez-Molina JA, Pérez-Ayala A, Moreno S, Fernández-González MC, Zamora J, López-Velez R. Use of benznidazole to treat chronic Chagas' disease: a systematic review with a meta-analysis. The Journal of antimicrobial chemotherapy. 2009;64(6):1139-47. doi: 10.1093/jac/dkp357.
48. Echeverria LE, Morillo CA. American Trypanosomiasis (Chagas Disease). Infect Dis Clin North Am. 2019;33(1):119-34. Epub 2019/02/05. doi: 10.1016/j.idc.2018.10.015. PubMed PMID: 30712757.
49. Villar JC, Herrera VM, Perez Carreno JG, Vaquiro Herrera E, Castellanos Dominguez YZ, Vasquez SM, et al. Nifurtimox versus benznidazole or placebo for asymptomatic Trypanosoma cruzi infection (Equivalence of Usual Interventions for Trypanosomiasis - EQUITY): study protocol for a randomised controlled trial. Trials.

2019;20(1):431. Epub 2019/07/17. doi: 10.1186/s13063-019-3423-3. PubMed PMID: 31307503; PubMed Central PMCID: PMCPMC6631895.
50. Villar JC, Herrera VM, Carreno JGP, Herrera EV, Dominguez YZC, Vasquez SM, et al. Correction to: Nifurtimox versus benznidazole or placebo for asymptomatic Trypanosoma cruzi infection (Equivalence of Usual Interventions for Trypanosomiasis - EQUITY): study protocol for a randomised controlled trial. Trials. 2019;20(1):516. Epub 2019/08/21. doi: 10.1186/s13063-019-3630-y. PubMed PMID: 31429793; PubMed Central PMCID: PMCPMC6701118.

APPENDICES

- **Appendix 1: Search strategy**
- **Appendix 2: Risk of bias assessment tool**
- **Appendix 3: Detailed description of included and ongoing studies** and **Excluded studies and reasons for exclusion**
- **Appendix 4: Support for judgement of included studies by risk of bias item** and **Risk of bias graph across all included studies**
- **Appendix 5: PRISMA Checklist**

Figure 1. Study flow diagram

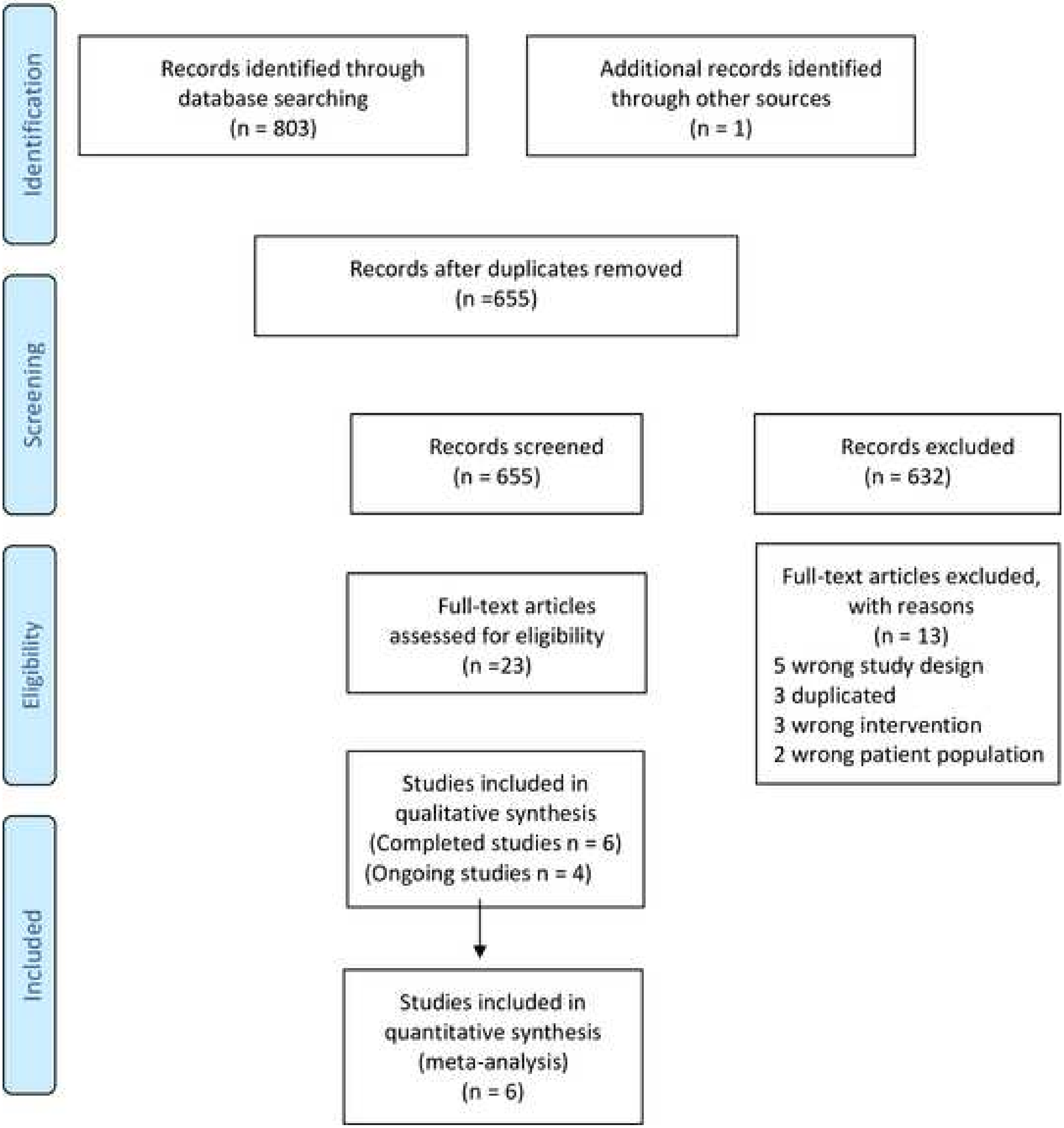

Figure 2 Click here to access/download;Figure;figure 2.tif

Figure 2 Risk of bias item for each included study

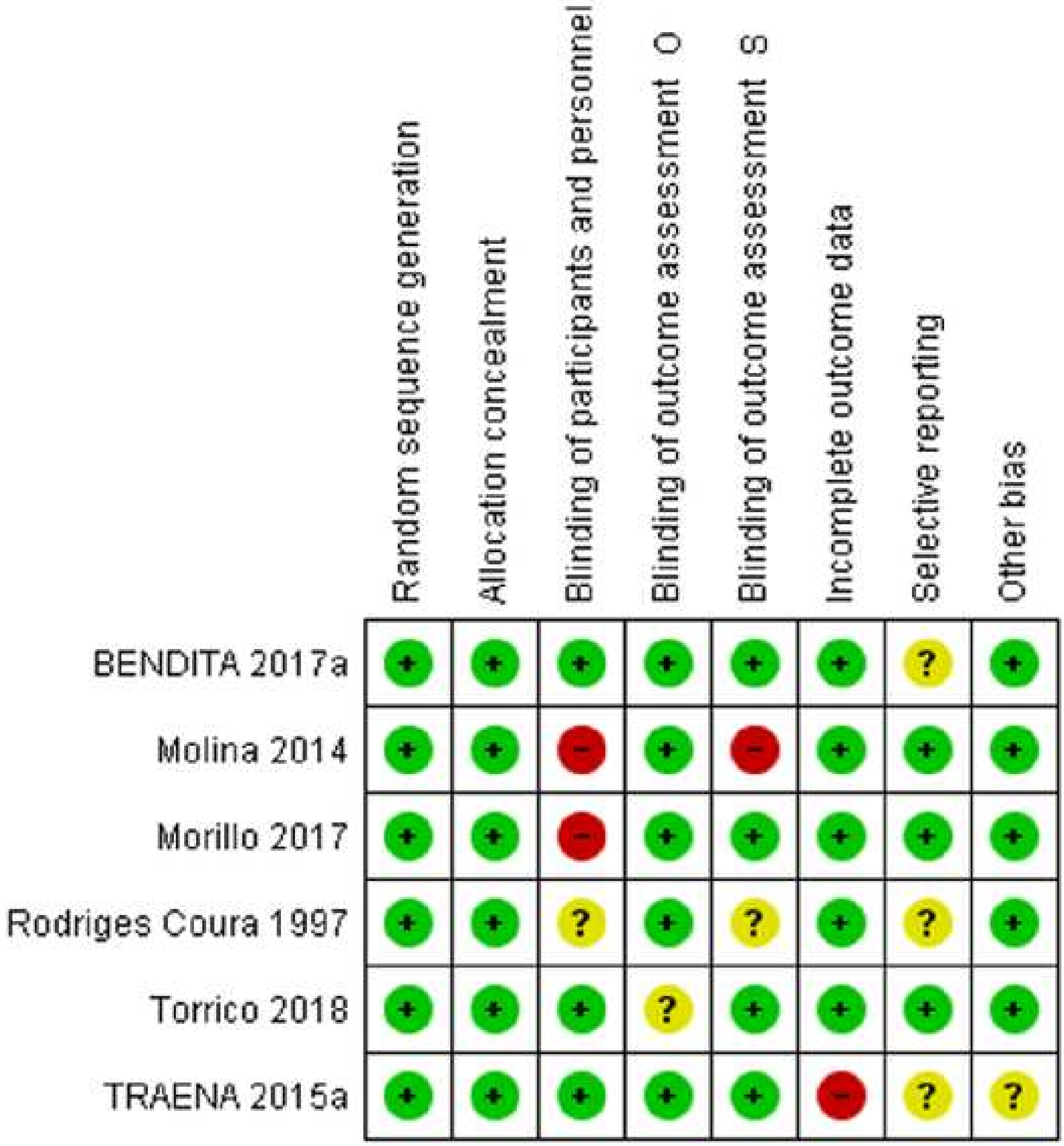

 Click here to access/download;Figure;Figure 3.tif

Figure 3. Error! Reference source not found. **at one year**

Study or Subgroup	Benznidazole Events	Benznidazole Total	Control Events	Control Total	Weight	Risk Ratio M-H, Random, 95% CI	Risk Ratio M-H, Random, 95% CI	Risk of Bias A B C D E F G
**3.1.1 Fixed dose**								
**Subtotal (95% CI)**		**0**		**0**		**Not estimable**		
Total events	0		0					
Heterogeneity: Not applicable								
Test for overall effect: Not applicable								
**3.1.3 Adjusted dose**								
Rodriges Coura 1997	26	26	24	24	48.1%	1.00 [0.93, 1.08]		
TRAENA 2015a	263	313	317	333	51.9%	0.88 [0.84, 0.93]		
**Subtotal (95% CI)**		**339**		**357**	**100.0%**	**0.94 [0.82, 1.08]**		
Total events	289		341					
Heterogeneity: Tau² = 0.01; Chi² = 8.82, df = 1 (P = 0.003); I² = 89%								
Test for overall effect: Z = 0.92 (P = 0.36)								
**Total (95% CI)**		**339**		**357**	**100.0%**	**0.94 [0.82, 1.08]**		
Total events	289		341					
Heterogeneity: Tau² = 0.01; Chi² = 8.82, df = 1 (P = 0.003); I² = 89%								
Test for overall effect: Z = 0.92 (P = 0.36)								
Test for subgroup differences: Not applicable								

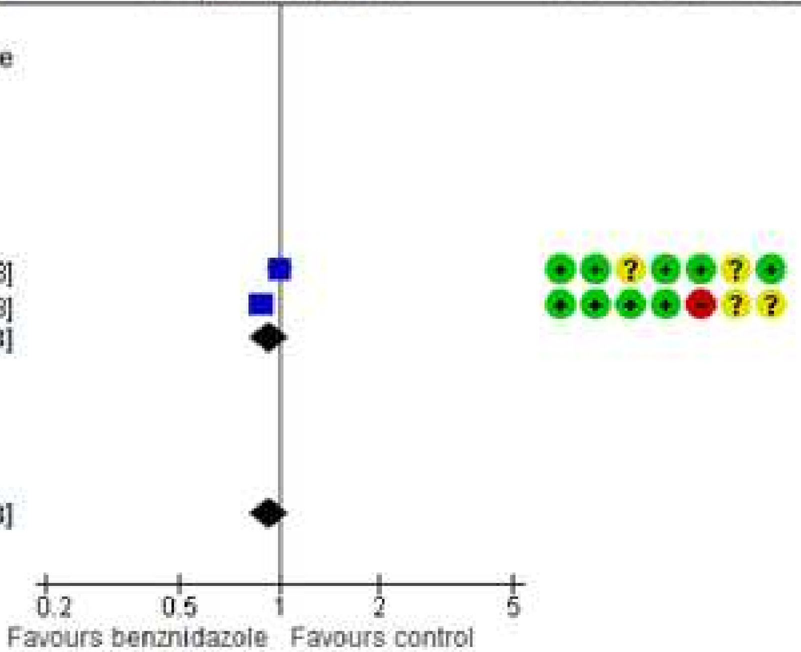

A) Random sequence generation (selection bias), **B)** allocation concealment (selection bias), **C)** blinding of participants and personnel (performance bias), **D)** blinding of outcome assessment objective outcomes (detection bias), **E)** incomplete outcome data (attrition bias), **F)** selective reporting (reporting bias), **H)** other bias

Figure 4 Click here to access/download;Figure;figure 4.tif

Figure 4. Positive PCR at one year

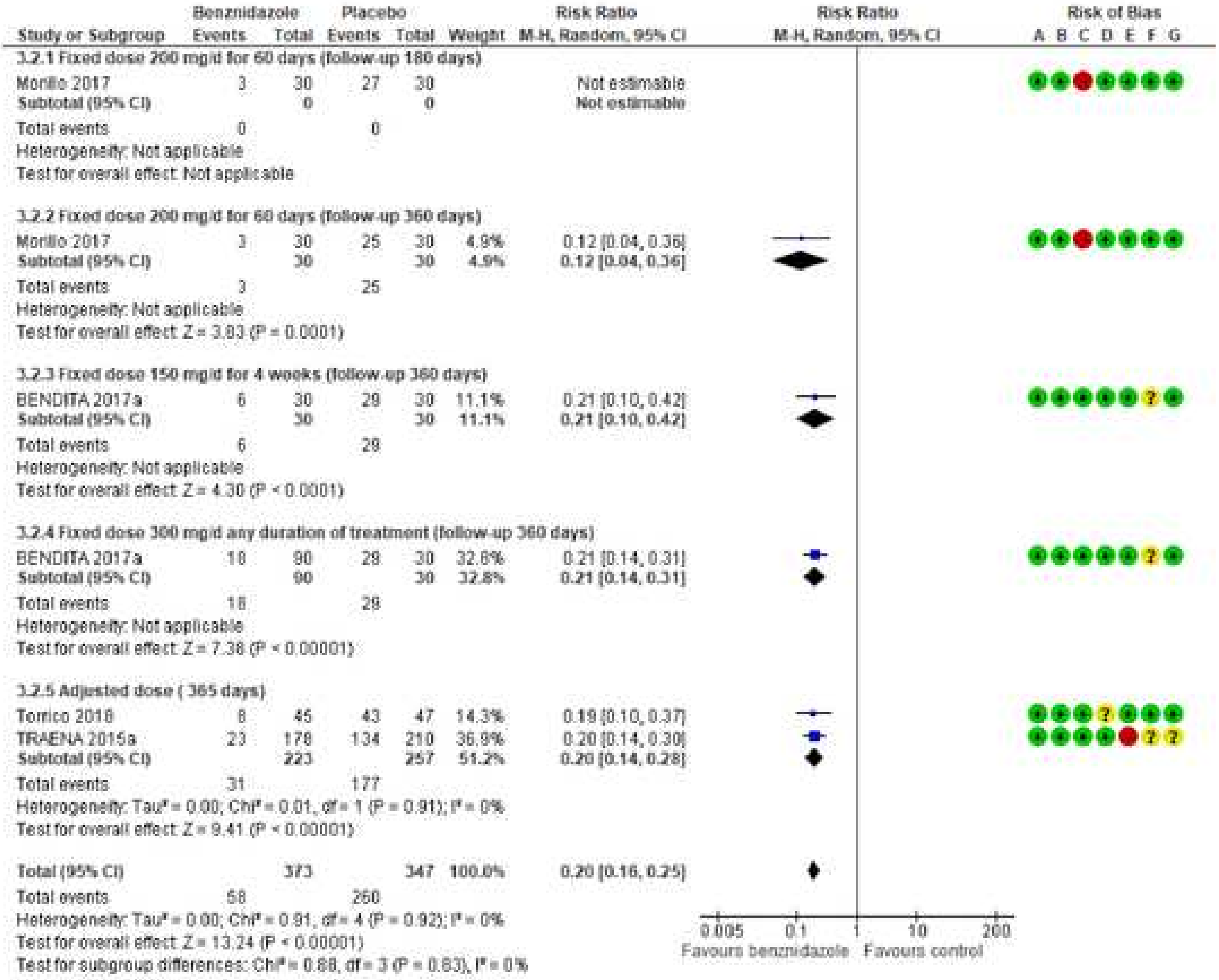

A) Random sequence generation (selection bias), **B)** allocation concealment (selection bias), **C)** blinding of participants and personnel (performance bias), **D)** blinding of outcome assessment objective outcomes (detection bias), **E)** incomplete outcome data (attrition bias), **F)** selective reporting (reporting bias), **H)** other bias

Figure 5

Click here to access/download;Figure;figure 5 .tif

Figure 5. Drug discontinuation

Study or Subgroup	Benznidazole Events	Benznidazole Total	Control Events	Control Total	Weight	Risk Ratio M-H, Random, 95% CI
**4.1.1 Fixed dose**						
Morillo 2017	10	30	1	30	20.3%	10.00 [1.36, 73.33]
**Subtotal (95% CI)**		**30**		**30**	**20.3%**	**10.00 [1.36, 73.33]**
Total events	10		1			
Heterogeneity: Not applicable						
Test for overall effect: Z = 2.27 (P = 0.02)						
**4.1.2 Adjusted dose**						
Rodriges Coura 1997	11	53	2	24	30.5%	2.49 [0.60, 10.38]
TRAENA 2015a	89	352	7	357	49.2%	12.89 [6.06, 27.44]
**Subtotal (95% CI)**		**405**		**381**	**79.7%**	**6.35 [1.27, 31.86]**
Total events	100		9			
Heterogeneity: $Tau^2 = 1.04$; $Chi^2 = 4.07$, df = 1 (P = 0.04); $I^2 = 75\%$						
Test for overall effect: Z = 2.25 (P = 0.02)						
**Total (95% CI)**		**435**		**411**	**100.0%**	**7.42 [2.51, 21.91]**
Total events	110		10			
Heterogeneity: $Tau^2 = 0.47$; $Chi^2 = 4.07$, df = 2 (P = 0.13); $I^2 = 51\%$						
Test for overall effect: Z = 3.63 (P = 0.0003)						
Test for subgroup differences: $Chi^2 = 0.12$, df = 1 (P = 0.73), $I^2 = 0\%$						

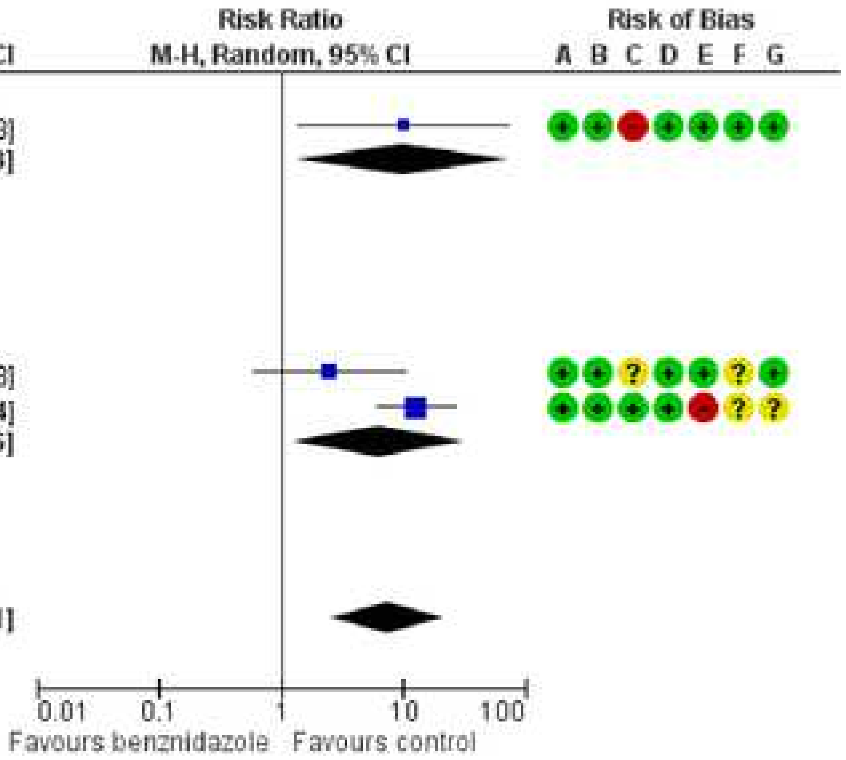

A) Random sequence generation (selection bias), B) allocation concealment (selection bias), C) blinding of participants and personnel (performance bias), D) blinding of outcome assessment objective outcomes (detection bias), E) incomplete outcome data (attrition bias), F) selective reporting (reporting bias), H) other bias

Figure 6. Peripheral neuropathy

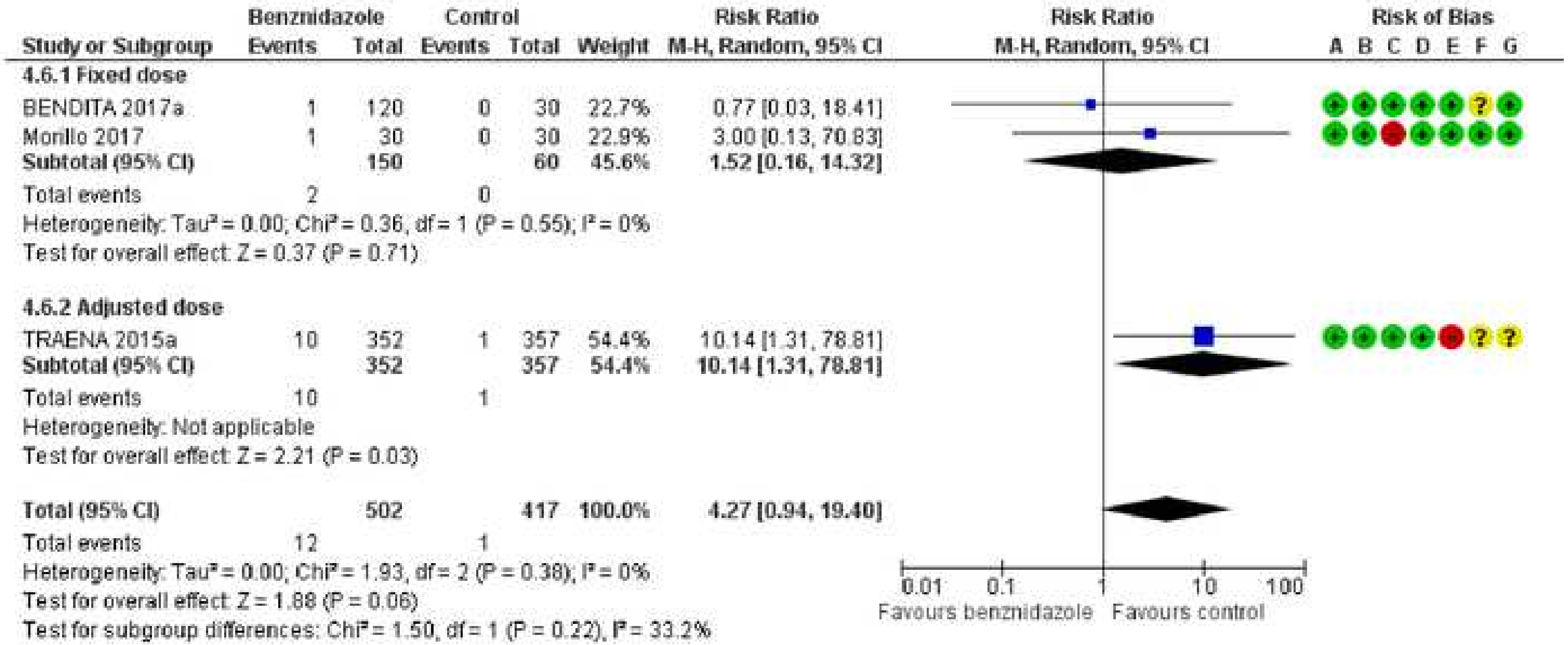

A) Random sequence generation (selection bias), **B)** allocation concealment (selection bias), **C)** blinding of participants and personnel (performance bias), **D)** blinding of outcome assessment objective outcomes (detection bias), **E)** incomplete outcome data (attrition bias), **F)** selective reporting (reporting bias), **H)** other bias

Figure 7 Click here to access/download;Figure;figure 7.tif

Figure 7. Mild rash

Study or Subgroup	Benznidazole Events	Benznidazole Total	Control Events	Control Total	Weight	Risk Ratio M-H, Random, 95% CI
**4.14.1 Fixed dose**						
BENDITA 2017a (1)	15	120	0	30	1.8%	7.94 [0.49, 129.10]
Morillo 2017	18	30	3	30	11.4%	6.00 [1.97, 18.25]
**Subtotal (95% CI)**		**150**		**60**	**13.2%**	**6.24 [2.22, 17.52]**
Total events	33		3			
Heterogeneity: Tau² = 0.00; Chi² = 0.04, df = 1 (P = 0.85); I² = 0%						
Test for overall effect: Z = 3.47 (P = 0.0005)						
**4.14.2 Adjusted dose**						
TRAENA 2015a	128	352	25	357	86.8%	5.19 [3.47, 7.77]
**Subtotal (95% CI)**		**352**		**357**	**86.8%**	**5.19 [3.47, 7.77]**
Total events	128		25			
Heterogeneity: Not applicable						
Test for overall effect: Z = 8.02 (P < 0.00001)						
**Total (95% CI)**		**502**		**417**	**100.0%**	**5.32 [3.66, 7.74]**
Total events	161		28			
Heterogeneity: Tau² = 0.00; Chi² = 0.14, df = 2 (P = 0.93); I² = 0%						
Test for overall effect: Z = 8.73 (P < 0.00001)						
Test for subgroup differences: Chi² = 0.10, df = 1 (P = 0.75), I² = 0%						

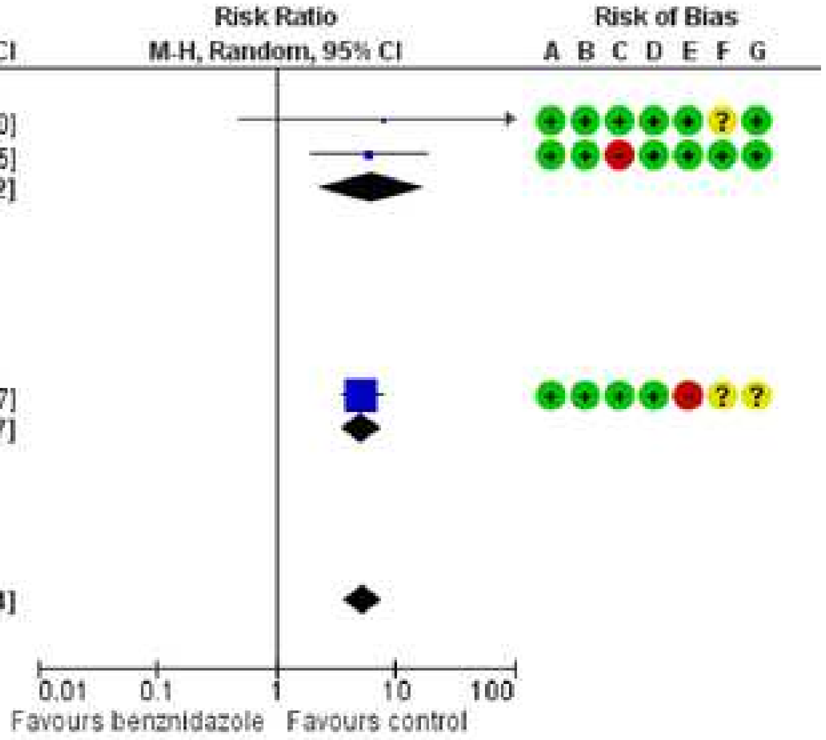

A) Random sequence generation (selection bias), **B)** allocation concealment (selection bias), **C)** blinding of participants and personnel (performance bias), **D)** blinding of outcome assessment objective outcomes (detection bias), **E)** incomplete outcome data (attrition bias), **F)** selective reporting (reporting bias), **H)** other bias

Click here to access/download
Supporting Information
Supporting information Fixed vs adjusted BZN.
SR&MA.docx

Click here to access/download
Supporting Information
Raw and analysis data.csv

Click here to access/download
Supporting Information
Revman file.htm

Dears

Helton da Costa Santiago, M.D., Ph.D

Associate Editor

PLOS Neglected Tropical Diseases

Alain Debrabant, Ph.D

Deputy Editor

PLOS Neglected Tropical Diseases

We fixed the texts, format and graphs and return as you suggested.
We answered point by point each issue to be solved.

- The text in lines 447- refers to serology outcome evaluation, but refers to figure 4, which show PCR data whereas supposedly it should refer to figure 3, which is very confusing. The title of what seems to be figure 3 is the same as figure 4, but the data apparently refers to a different data set, as it is supposed to be. However, it is not clear by looking at the figure what is the data being displayed.

We are sorry. It is confusing because the submission in pdf does present this problem but the file in the word file that was already uploaded does not. We checked now that the submission in pdf reflected the corrected word file version.

In addition, we would like to request the authors to follow authors guidelines in formatting the manuscript.

- Although, Plos NTDs might be very flexible in a first submission regarding to formatting, there are several problems with the manuscript that makes the reading annoying:

  1- References are repeated in between methods sessions and in the end of the manuscript

    We cited 48 unique references, instead of 50, all at the end of the manuscript now. We found two duplications:
    #28 and #34 Rodrigues Coura J, de Abreu LL, Willcox HP, Petana W. [Comparative controlled study on the use of benznidazole, nifurtimox and placebo, in the chronic form of Chagas' disease, in a field area with interrupted transmission. I. Preliminary evaluation]. Rev Soc Bras Med Trop. 1997;30(2):139-144.

#40 and #47 Pérez-Molina JA, Pérez-Ayala A, Moreno S, Fernández-González MCMC, Zamora J, López-Velez R. Use of benznidazole to treat chronic Chagas' disease: A systematic review with a meta-analysis. Journal of Antimicrobial Chemotherapy. 2009;64(6):1139-47. doi: 10.1093/jac/dkp357.

2- All tables are repeated in the methods session and in the end of the manuscript
Corrected now.

3- Legend of table 2 is misplaced between the discussion session.
Corrected now

Please, proceed to fix the texts, formatting and graphs and return the manuscript for proper editorial flow as soon as possible.

Figure Files:
While revising your submission, please upload your figure files to the Preflight Analysis and Conversion Engine (PACE) digital diagnostic tool, https://pacev2.apexcovantage.com. PACE helps ensure that figures meet PLOS requirements. To use PACE, you must first register as a user. Then, login and navigate to the UPLOAD tab, where you will find detailed instructions on how to use the tool. If you encounter any issues or have any questions when using PACE, please email us at figures@plos.org.
We present now all the figures separately after Conversion Engine (PACE)

Data Requirements:
Please note that, as a condition of publication, PLOS' data policy requires that you make available all data used to draw the conclusions outlined in your manuscript. Data must be deposited in an appropriate repository, included within the body of the manuscript, or uploaded as supporting information. This includes all numerical values that were used to generate graphs, histograms etc.. For an example see here: http://www.plosbiology.org/article/info%3Adoi%2F10.1371%2Fjournal.pbio.1001908#s5.
We uploaded the RevMan 5-derived htlm file and excel file with analysis data as supporting information

Reproducibility:
To enhance the reproducibility of your results, PLOS recommends that you deposit laboratory protocols in protocols.io, where a protocol can be assigned its own identifier (DOI) such that it can be cited independently in the future. For instructions see http://journals.plos.org/plosntds/s/submission-guidelines#loc-materials-and-methods

In compliance with data protection regulations, you may request that we remove your personal registration details at any time. (Use the following URL: https://www.editorialmanager.com/pntd/login.asp?a=r). Please contact the publication office if you have any questions.

Not applicable (not a laboratory protocol)
