# Supplementary material for: Fixed vs adjusted-dose benznidazole for adults with chronic Chagas disease without cardiomyopathy: A systematic review and meta-analysis
Source: PLoS Negl Trop Dis. 2020 Aug 17;14(8):e0008529. doi: 10.1371/journal.pntd.0008529 (PMC7451967; doi:10.1371/journal.pntd.0008529)
Supplement: Attachment — Submitted filename: Response to reviwers.docx [file pntd.0008529.s008.docx]

Dears

Helton da Costa Santiago, M.D., Ph.D
Associate Editor
PLOS Neglected Tropical Diseases

Alain Debrabant, Ph.D
Deputy Editor
PLOS Neglected Tropical Diseases

We fixed the texts, format and graphs and return as you suggested.

We answered point by point each issue to be solved.

- The text in lines 447- refers to serology outcome evaluation, but refers to figure 4, which show PCR data whereas supposedly it should refer to figure 3, which is very confusing. The title of what seems to be figure 3 is the same as figure 4, but the data apparently refers to a different data set, as it is supposed to be. However, it is not clear by looking at the figure what is the data being displayed.

We are sorry. It is confusing because the submission in pdf does present this problem but the file in the word file that was already uploaded does not. We checked now that the submission in pdf reflected the corrected word file version.

In addition, we would like to request the authors to follow authors guidelines in formatting the manuscript.

- Although, Plos NTDs might be very flexible in a first submission regarding to formatting, there are several problems with the manuscript that makes the reading annoying:

  1- References are repeated in between methods sessions and in the end of the manuscript

We cited 48 unique references, instead of 50, all at the end of the manuscript now. We found two duplications:

**#28 and #34** Rodrigues Coura J, de Abreu LL, Willcox HP, Petana W. [Comparative controlled study on the use of benznidazole, nifurtimox and placebo, in the chronic form of Chagas' disease, in a field area with interrupted transmission. I. Preliminary evaluation]. Rev Soc Bras Med Trop. 1997;30(2):139-144.

**#40 and #47** Pérez-Molina JA, Pérez-Ayala A, Moreno S, Fernández-González MCMC, Zamora J, López-Velez R. Use of benznidazole to treat chronic Chagas' disease: A systematic review with a meta-analysis. Journal of Antimicrobial Chemotherapy. 2009;64(6):1139-47. doi: 10.1093/jac/dkp357.

2- All tables are repeated in the methods session and in the end of the manuscript

Corrected now.

3- Legend of table 2 is misplaced between the discussion session.

Corrected now

Please, proceed to fix the texts, formatting and graphs and return the manuscript for proper editorial flow as soon as possible.


**Figure Files:**
While revising your submission, please upload your figure files to the Preflight Analysis and Conversion Engine (PACE) digital diagnostic tool, [https://pacev2.apexcovantage.com](https://pacev2.apexcovantage.com/). PACE helps ensure that figures meet PLOS requirements. To use PACE, you must first register as a user. Then, login and navigate to the UPLOAD tab, where you will find detailed instructions on how to use the tool. If you encounter any issues or have any questions when using PACE, please email us at [figures@plos.org](mailto:figures@plos.org).

We present now all the figures separately after Conversion Engine (PACE)
 
**Data Requirements:**
Please note that, as a condition of publication, PLOS' data policy requires that you make available all data used to draw the conclusions outlined in your manuscript. Data must be deposited in an appropriate repository, included within the body of the manuscript, or uploaded as supporting information. This includes all numerical values that were used to generate graphs, histograms etc.. For an example see here: <http://www.plosbiology.org/article/info%3Adoi%2F10.1371%2Fjournal.pbio.1001908#s5>.

We uploaded the RevMan 5-derived htlm file and excel file with analysis data as supporting information
 
Reproducibility:
To enhance the reproducibility of your results, PLOS recommends that you deposit laboratory protocols in [protocols.io](http://protocols.io/), where a protocol can be assigned its own identifier (DOI) such that it can be cited independently in the future. For instructions see <http://journals.plos.org/plosntds/s/submission-guidelines#loc-materials-and-methods>

__________________________________________________
In compliance with data protection regulations, you may request that we remove your personal registration details at any time.  (Use the following URL: <https://www.editorialmanager.com/pntd/login.asp?a=r>). Please contact the publication office if you have any questions.

Not applicable (not a laboratory protocol)
