# Supplementary material for: Fixed vs adjusted-dose benznidazole for adults with chronic Chagas disease without cardiomyopathy: A systematic review and meta-analysis
Source: PLoS Negl Trop Dis. 2020 Aug 17;14(8):e0008529. doi: 10.1371/journal.pntd.0008529 (PMC7451967; doi:10.1371/journal.pntd.0008529)
Supplement: Attachment — Submitted filename: Answer to PLOS NTD reviewers comments.docx [file pntd.0008529.s010.docx]

**PLOS Neglected Tropical Diseases**

 June 19, 2020

Dear Dr. Ciapponi,
 
Thank you very much for submitting your manuscript *"Fixed vs adjusted-dose benznidazole for adults with chronic Chagas disease without cardiomyopathy: A systematic review and meta-analysis"* for consideration at PLOS Neglected Tropical Diseases. As with all papers reviewed by the journal, your manuscript was reviewed by members of the editorial board and by several independent reviewers. The reviewers appreciated the attention to an important topic. Based on the reviews, we are likely to accept this manuscript for publication, providing that you modify the manuscript according to the review recommendations.
 
The manuscript was evaluated by three experts in field. One reviewer rejected the manuscript based on the limited number papers found to be included in the meta-analysis. The editors agree that the limited number of manuscript imposes a limitation, but we acknowledge that the authors are aware of the limitations, which are disclosed in the discussion. We are requesting the authors to perform **minor revision** as they find appropriate on the suggestions offered by reviewer #3.
 
Please prepare and submit your revised manuscript within 30 days. If you anticipate any delay, please let us know the expected resubmission date by replying to this email.  
When you are ready to resubmit, please upload the following:

[1] **A letter containing a detailed list of your responses to all review comments**, and a **description of the changes you have made in the manuscript**. 
Please note while forming your response, if your article is accepted, you may have the opportunity to make the peer review history publicly available. The record will include editor decision letters (with reviews) and your responses to reviewer comments. If eligible, we will contact you to opt in or out

[2] **Two versions of the revised manuscript**: **one** with either highlights or **tracked changes** denoting where the text has been changed; the other a **clean version** (uploaded as the manuscript file).

Important additional instructions are given below your reviewer comments.

Thank you again for your submission to our journal. We hope that our editorial process has been constructive so far, and we welcome your feedback at any time. Please don't hesitate to contact us if you have any questions or comments.
 
Sincerely,

Helton da Costa Santiago, M.D., Ph.D
Associate Editor
PLOS Neglected Tropical Diseases

Alain Debrabant, Ph.D.
Deputy Editor
PLOS Neglected Tropical Diseases
***********************
The manuscript was evaluated by three experts in field. **One reviewer rejected the manuscript based on the limited number papers** found to be included in the meta-analysis. The editors agree that the limited number of manuscript imposes a limitation, but we acknowledge that the authors are aware of the limitations, which are **disclosed in the discussion**. **We are requesting the authors to perform minor revision as they find appropriate on the suggestions offered by reviewer #3.**

We have implemented all minor revisions suggested in the pdf.

Concerning about using “tests' positivity” as the measure of efficacy we prefer keep this approach because it was stated in our protocol (PROSPERO CRD42019120905). Besides it is the outcome used by many authors, including a Cochrane review (Villar JC, Perez JG, Cortes OL, Riarte A, Pepper M, Marin-Neto JA, Guyatt GH. Trypanocidal drugs for chronic asymptomatic Trypanosoma cruzi infection.
Cochrane Database of Systematic Reviews 2014, Issue 5. Art. No.: CD003463. DOI: 10.1002/14651858.CD003463.pub2.). This facilitate readers to compare results

**Reviewer's Responses to Questions**
Key Review Criteria Required for Acceptance?
As you describe the new analyses required for acceptance, please consider the following:

**Methods**
-Are the objectives of the study clearly articulated with a clear testable hypothesis stated?
-Is the study design appropriate to address the stated objectives?
-Is the population clearly described and appropriate for the hypothesis being tested?
-Is the sample size sufficient to ensure adequate power to address the hypothesis being tested?
-Were correct statistical analysis used to support conclusions?
-Are there concerns about ethical or regulatory requirements being met?

**Reviewer #1**: While the authors present an interesting premise this study has some issues.
They propose to determine the comparative safety and efficacy of a fixed dose of benznidazole (BZN) with an adjusted dose for the treatment of T. cruzi seropositive adults without cardiomyopathy through a systematic review and a meta-analysis.
After the search they found ten studies that meet the inclusion criteria. However, four of them are ongoing and two are unpublished. So, these studies should not be included in the analysis. They did not find any study comparing a fixed dose X an adjusted dose of BZN. All these together make difficult to perform a meta-analysis study and address this important question.
So, this manuscript should be rejected

We agree with the editor regarding the limited number of included studies in the meta-analysis, but disclosed this situation in the discussion section.

**Reviewer #2**: In my opinion, after authors revision, the study is clearly articulated with hypothesis, the design is appropriate  to the stated objectives. And, despite of sample size is very low the conclusion is suitable from found results.

Thank you

**Reviewer #3**: The study objective was properly stated. Method and adequate analysis were done.
Thank you

--------------------

**Results**
-Does the analysis presented match the analysis plan?
-Are the results clearly and completely presented?
-Are the figures (Tables, Images) of sufficient quality for clarity?

**Reviewer #1**: Figure 3 is missing

Figure 3 is in the PNTD pdf.

**Reviewer #2**: The results are presented clearly and they are completely matched to analysis plan.
Thank you

**Reviewer #3**: The results met planned analysis and were presented clearly. Study importance as well as limitations were adequately described.
Thank you

--------------------

**Conclusions**
-Are the conclusions supported by the data presented?
-Are the limitations of analysis clearly described?
-Do the authors discuss how these data can be helpful to advance our understanding of the topic under study?
-Is public health relevance addressed?

**Reviewer #1**: Conclusions can not be supported by the meta-analysis performed. Among the ten studies selected two of them  have not yet  been published and four  were ongoing studies and should no be included in the analysis.

We present the ongoing studies to alert the readers about further research that could change results in the future.

Systematic Review methodology strongly recommends to include non-published studies to prevent publication bias. Nevertheless, every study was assessed about risk of bias with the Cochrane tool.

**Reviewer #2**: The conclusion is supported by the data presented, the limitations are presented and clearly described.

Thank you

**Reviewer #3**: The conclusions provided are in accord with results presented and data analysed in the study.

Thank you

--------------------

Editorial and Data Presentation Modifications?
Use  this section for editorial suggestions as well as relatively minor modifications of existing data that would enhance clarity. If the only modifications needed are minor and/or editorial, you may wish to recommend “Minor Revision” or “Accept”.

**Reviewer #1**: NO

**Reviewer #2**: There is no necessity of data modification, after authors revision.

**Reviewer #3**: (No Response)

--------------------

**Summary and General Comments**
Use  this section to provide overall comments, discuss strengths/weaknesses of the study, novelty, significance, general execution and scholarship. You may also include additional comments for the author, including concerns about dual publication, research ethics, or publication ethics. If requesting major revision, please articulate the new experiments that are needed.

**Reviewer #1**: (No Response)

**Reviewer #2**: Despite of small number of eligible studies the paper is important because direction of specific Chagas disease treatment is not is not clear yet.

We agree with the reviewer

**Reviewer #3**: The study evaluated and reviewed available literature to understand differential in treatment dosage for chagas disease. The result of the meta-analysis provide useful and contribute to existing knowledge about the disease and more importantly, serve as a background on which a more significant experimental study can be conducted.
We agree with the reviewer

--------------------

PLOS authors have the option to publish the peer review history of their article "<https://journals.plos.org/plosntds/s/editorial-and-peer-review-process#loc-peer-review-history>" what does this mean?). If published, this will include your full peer review and any attached files.
We agree to publish the peer review history of our article

If you choose “no”, your identity will remain anonymous but your review may still be made public.

Do you want your identity to be public for this peer review? For information about this choice, including consent withdrawal, please see our "<https://www.plos.org/privacy-policy>" Privacy Policy.

**Reviewer** #1: No

**Reviewer** #2: No

**Reviewer** #3: Yes: Adebiyi Adeniran


**Figure Files:**
While revising your submission, please upload your figure files to the Preflight Analysis and Conversion Engine (PACE) digital diagnostic tool, [https://pacev2.apexcovantage.com](https://pacev2.apexcovantage.com/). PACE helps ensure that figures meet PLOS requirements. To use PACE, you must first register as a user. Then, login and navigate to the UPLOAD tab, where you will find detailed instructions on how to use the tool. If you encounter any issues or have any questions when using PACE, please email us at [figures@plos.org](mailto:figures@plos.org).
 
**Data Requirements:**
Please note that, as a condition of publication, PLOS' data policy requires that you make available all data used to draw the conclusions outlined in your manuscript. Data must be deposited in an appropriate repository, included within the body of the manuscript, or uploaded as supporting information. This includes all numerical values that were used to generate graphs, histograms etc.. For an example see here: <http://www.plosbiology.org/article/info%3Adoi%2F10.1371%2Fjournal.pbio.1001908#s5>.
 
**Reproducibility:**
To enhance the reproducibility of your results, PLOS recommends that you deposit laboratory protocols in [protocols.io](http://protocols.io/), where a protocol can be assigned its own identifier (DOI) such that it can be cited independently in the future. For instructions see <http://journals.plos.org/plosntds/s/submission-guidelines#loc-materials-and-methods>
